# Increasing CO_2_ levels fertilize C_4_ grass production

**DOI:** 10.1038/s41586-026-10935-4

**Published:** 2026-08-19

**Authors:** Kimberley J. Simpson, A. Carla Staver, James A. King, William J. Bond, Corli Coetsee, Nita C. M. Pallett, Adam F. A. Pellegrini, Sarah L. Raubenheimer, Brad S. Ripley, Maria Val Martin, Colin P. Osborne

**Affiliations:** 1 Plants, Photosynthesis and Soil, School of Biosciences, University of Sheffield, Sheffield, UK; 2 Botany Department, Rhodes University, Makhanda, South Africa; 3 Department of Ecology and Evolutionary Biology, Princeton University, Princeton, NJ USA; 4 Department of Biological Sciences, University of Cape Town, Cape Town, South Africa; 5 Scientific Services, South African National Parks, Skukuza, South Africa; 6 School of Natural Resource Management, Nelson Mandela University, George, South Africa; 7 Earth System Science Department, Stanford University, Stanford, CA USA; 8 Institute for Global Change Biology, School for Environment and Sustainability, University of Michigan, Ann Arbor, MI USA

**Keywords:** Climate-change ecology, Plant ecology, Plant physiology, Grassland ecology

## Abstract

Rising atmospheric CO_2_ concentrations are impacting the global terrestrial biosphere through indirect climate effects and direct effects on plant performance^1–3^. In tropical forests, long-term monitoring indicates a substantial CO_2_-driven carbon sink^4^. C_4_-grass-dominated tropical and subtropical savannas contribute approximately 30% of terrestrial net primary production^5^, and yet equivalent long-term analyses of CO_2_ responses are lacking. Here we show a clear and consistent result across a meta-analysis of 70 CO_2_-addition experiments and 32 years of in situ field observations from southern Africa: CO_2_ fertilization of wild C_4_ grasses is widespread in dry conditions. In experiments, grasses reduced stomatal conductance under higher levels of CO_2_, limiting water loss while increasing carbon gain. In the field, improved water use efficiency translated into increased C_4_ grass biomass production across three decades of observations. Finally, simulations via the Community Land Model^6^ suggest that CO_2_ fertilization of C_4_ grass aboveground productivity may continue to increase under future conditions. Together, these results challenge the view that C_4_ grasses are unresponsive to increasing levels of CO_2_, demonstrating instead that annual aboveground production of grasses in the field in southern Africa has increased by 28% over three decades (a CO_2_-driven increase of 75.1 g m^−2^ (95% confidence interval of 74.5–75.8 g m^−2^) or 0.37 tons C ha^−1^ of annual production). Although the fate of this carbon is uncertain (depending on feedbacks with fire, herbivory and woody vegetation), effects on the global carbon cycle may be profound.

## Abstract (web summary)

By combining a meta-analysis of CO_2_ enrichment experiments with three decades of southern African savanna observations, an increase in biomass production by C_4_ grasses under rising CO_2_ levels in water-limited environments is revealed.

## Main

The potential for direct CO_2_ effects on plant productivity has long been recognized, motivating a network of free-air CO_2_ enrichment (FACE) experiments^7,8^. These show widespread but context-dependent direct CO_2_ effects, including increased photosynthesis, reduced stomatal conductance and water use, and increased biomass production^9,10^. However, in situ CO_2_ enrichment experiments have largely omitted major tropical and subtropical ecosystems^7,8^. In tropical forests, long-term vegetation monitoring is filling that gap, strongly implicating CO_2_ fertilization as a driver of enhanced tree productivity^4^. In tropical and subtropical savannas, long-term observations and modelling have also suggested a role for CO_2_ fertilization as a driver of C_3_ woody plant encroachment^11,12^. However, although appropriate monitoring data are available^13^, equivalent long-term analyses of CO_2_ responses in C_4_ savanna grasses are lacking. This uncertainty means that net outcomes for tree–grass dynamics remain unclear, including possible effects on fire regimes^14^, limiting our ability to model and forecast savanna responses to global change.

In situ experiments in temperate grasslands and chamber experiments with savanna grasses provide a basis for generating reasonable hypotheses for how tropical C_4_ grasses will respond to increasing levels of CO_2_. Because the C_4_ photosynthetic pathway evolved as a CO_2_-concentrating mechanism that suppresses photorespiration, C_4_ grasses are predicted to show smaller direct photosynthetic responses to rising atmospheric CO_2_ levels than C_3_ grasses. In C_4_ crops, which have undergone intensive selection for high yields, this CO_2_-concentrating mechanism eliminates CO_2_ fertilization under well-watered conditions^15^. However, photosynthesis in wild C_4_ grasses is less efficient, and CO_2_ fertilization has been observed across a diversity of wild C_4_ grass species^16^. Although this may be partially due to enhanced activity of the photosynthetic enzyme Rubisco, higher levels of CO_2_ also reduce stomatal aperture, alleviating water deficits to promote carbon fixation. FACE experiments with C_4_ crops show that this mechanism enables CO_2_ fertilization under water scarcity^10^. Field-based experiments in mixed C_3_–C_4_ North American temperate prairies have also shown that grass productivity is enhanced by FACE, especially in seasonally dry environments, in dry years, at drier sites and under high soil nutrient availability^8,9,17^ (but see ref. ^18^). However, existing syntheses of field experiments do not disentangle C_3_ from C_4_ plant responses, and existing quantitative synthesis of smaller-scale experiments is out of date^16^ and includes only a subset of experiments^8,9^. Together, these stop short of evaluating in situ grass production in wild C_4_-dominated systems.

Here we provide a comprehensive synthesis of C_4_ savanna grass responses to rising levels of CO_2_, combining a meta-analysis of experiments, new field observations and a vegetation model case study. First, we used a meta-analysis of CO_2_-enrichment experiments to evaluate whether CO_2_ fertilization is globally widespread across wild C_4_ grasses and interacts with water availability. Second, we analysed a unique 32-year time series of savanna grass aboveground biomass from 533 plots in the Kruger National Park, South Africa (‘Kruger’ henceforth), to evaluate whether experimental findings translate into in situ biomass production. Finally, we used a land surface model (the Community Land Model (CLM)^6^ to explore potential outcomes for grassy ecosystems in the future. Combining independent lines of evidence overcomes limitations inherent in previous studies and enables us to evaluate whether hypothesized CO_2_ fertilization of C_4_ savanna grasses is increasing their production in the field.

## Rising CO_2_ levels fertilize wild C_4_ grasses

**Fig. 1:**
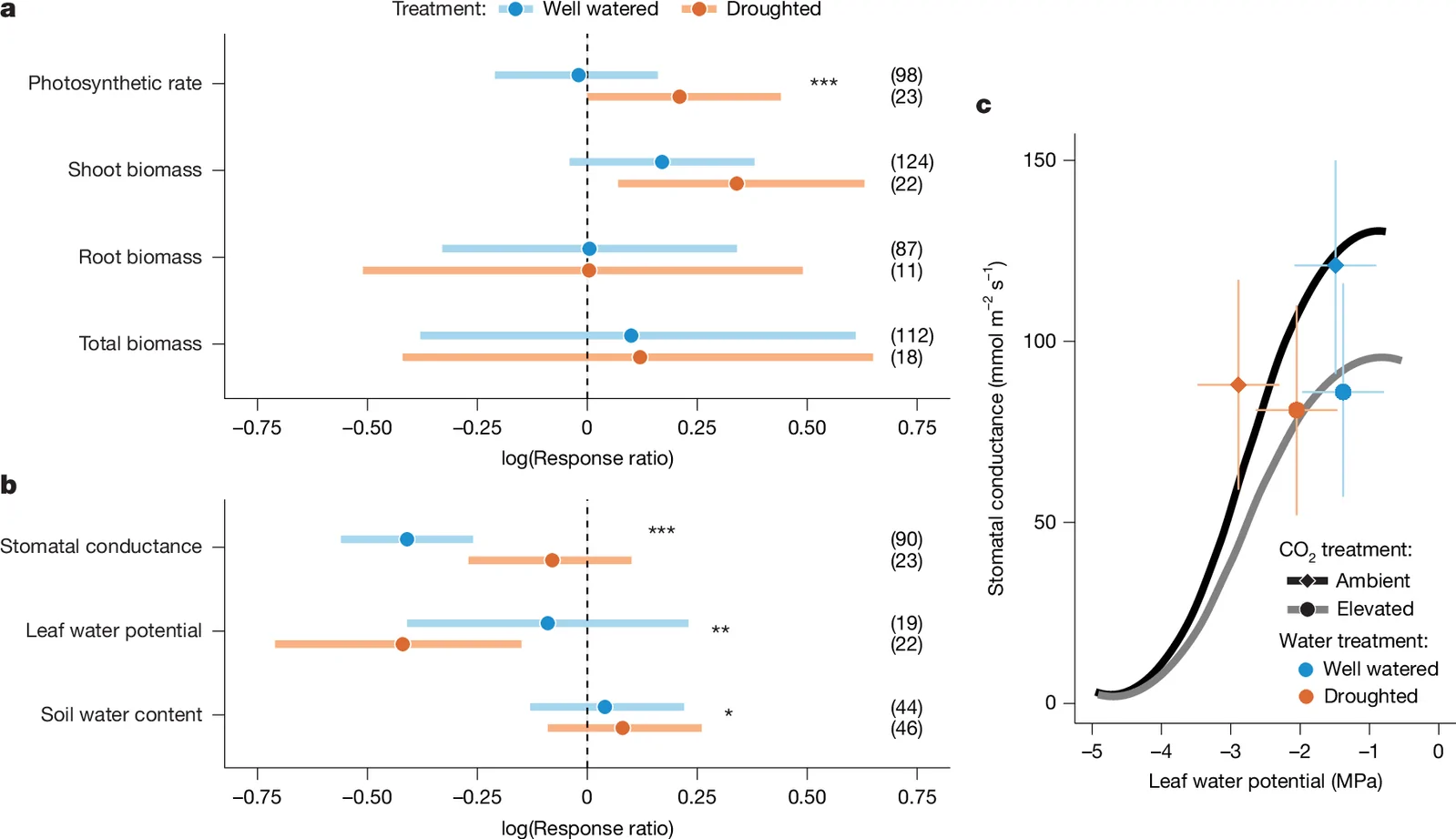
Meta-analysis of wild C_4_ grass responses to elevated CO_2_ levels under different water availability. **a**,**b**, Responses of grass productivity traits (**a**) and water-relation traits (**b**) are expressed as logRR (the natural log of the ratio of values under elevated versus ambient CO_2_ levels). Inference was based on Bayesian multilevel meta-analytic models fitted using MCMCglmm. The points indicate the estimated marginal mean logRR, the coloured bars show 95% CIs and sample sizes are given in brackets. Effects were considered significant where CIs did not overlap zero (dashed vertical lines). Water treatment had significant effects on responses for photosynthesis (*P* = 0.0006), stomatal conductance (*P* = 0.00001), leaf water potential (*P* = 0.0008) and soil water content (*P* = 0.03). No one-sided tests or multiple-comparison adjustments were used. **c**, Meta-analysis estimates of absolute changes in stomatal conductance under declining water availability, indicated by more negative leaf water potential. The points and error bars show estimated marginal mean ± 1 s.e. Sample sizes (for both ambient and elevated levels of CO_2_) were *n* = 38 well watered and *n* = 44 droughted for leaf water potential, and *n* = 52 well watered and *n* = 49 droughted for stomatal conductance. The lines show modelled stomatal responses for a representative savanna grass, parameterized using *Themeda triandra*, based on hydraulic–stomatal modelling equations ((1)–(3)). The asterisks show significant differences between droughted and well-watered treatments (*P < 0.05, **P < 0.01 and ***P < 0.001).

**Extended Data Fig. 1:**
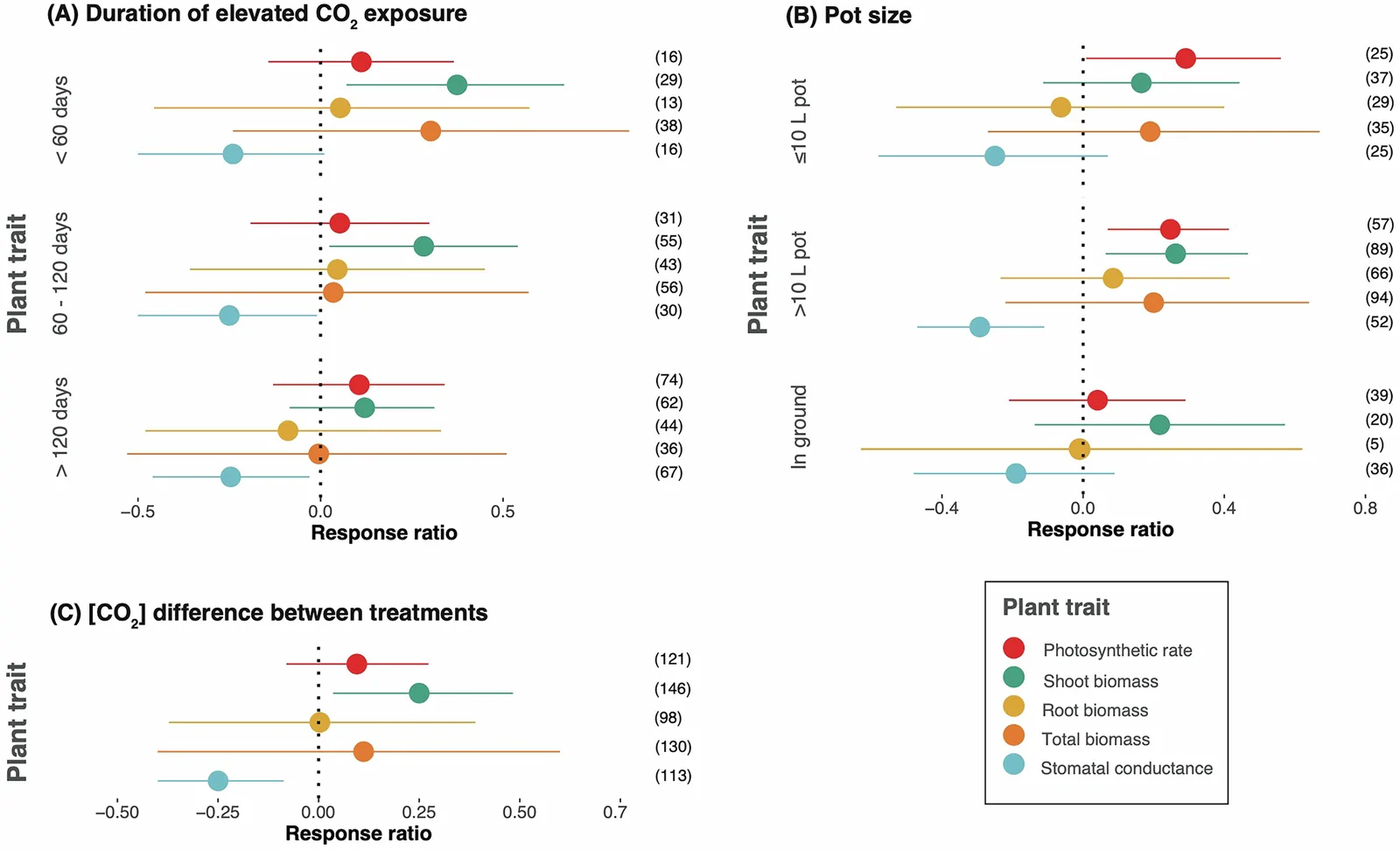
Meta-analysis coefficients for experimental factors. The coefficients extracted for the MCMCglmm meta-analyses relating to three experimental factors included as additional fixed effects in models: A) the duration of elevated CO_2_ exposure, B) pot size and C) difference in CO_2_ concentration between ambient and elevated CO_2_ treatments (mean difference in CO_2_ concentration between treatments = 332 ppm). Points and bars indicate the estimated marginal means and 95% confidence intervals of log-response ratios (the natural log of the ratio of measured values at elevated to ambient CO_2_). The response ratios for a plant trait did not differ between levels of these explanatory variables except in the following cases: response ratios for total biomass were higher (P < 0.05) in experiments <60 days than in experiments >60 days; response ratios for shoot biomass were higher (P < 0.05) in experiments <60 days than in experiments >120 days; and response ratios for photosynthetic rate were higher (P < 0.05) for plants grown in >10 L pots than those grown in the ground. Sample sizes are given in brackets.

**Extended Data Fig. 2:**
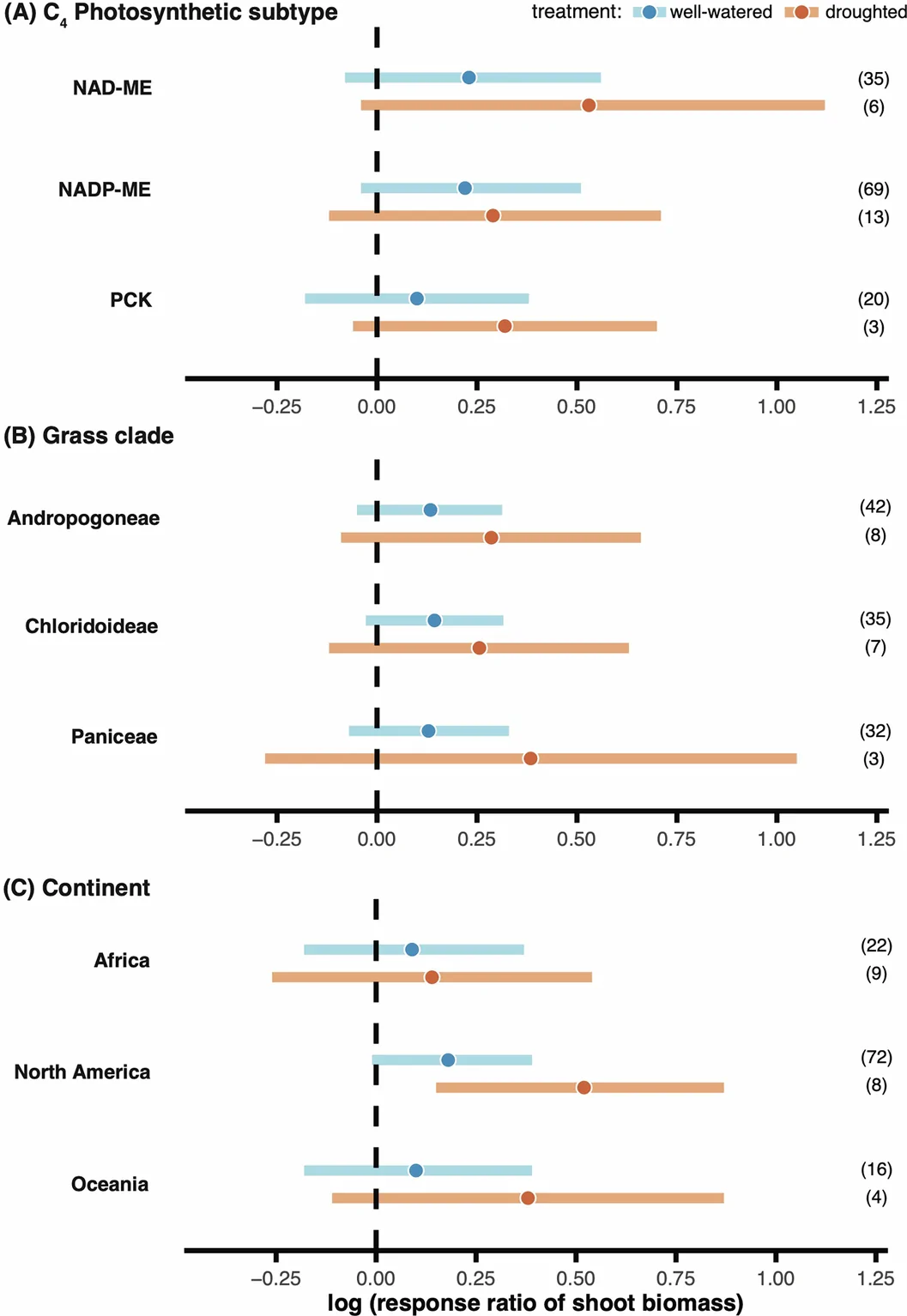
Meta-analysis of wild C_4_ grass shoot biomass responses to elevated CO_2_ divided into C_4_ photosynthetic subtype (A), grass clade (B) and continent (C). The responses of grass shoot biomass to elevated CO_2_ are displayed as log-response ratios (logRR; the natural log of the ratio of measured values at elevated to ambient CO_2_). Points represent estimated marginal means and bars show the 95% confidence intervals. Sample sizes are given in brackets.

Systematic meta-analysis of 70 experimental studies showed that CO_2_ fertilization of aboveground biomass is widespread among wild C_4_ grasses under water limitation (estimated marginal mean log response ratio under elevated levels of CO_2_ (logRR-CO_2_) = 0.34 (95% confidence interval: 0.06–0.63)), but not well-watered conditions (logRR-CO_2_ = 0.16 (−0.04 to 0.37); Fig. 1a). Photosynthetic rate also increased under elevated levels of CO_2_ in water-limited plants (logRR-CO_2_ = 0.21 (0.00–0.42); Fig. 1a). Variable responses of root biomass to elevated levels of CO_2_ (logRR-CO_2_ = 0.00 (−0.31 to 0.36) for well-watered plants; logRR-CO_2_ = 0.00 (−0.49 to 0.50) for water-limited plants) contributed to variation in total (aboveground plus belowground) biomass trends (logRR-CO_2_ = 0.10 (−0.39 to 0.60) for well-watered plants; logRR-CO_2_ = 0.12 (−0.41 to 0.68) for water-limited plants). Experimental factors (CO_2_ treatment size, pot size and experimental duration) had minimal effects on grass trait responses (Extended Data Fig. 1). In contrast with a previous meta-analysis on C_4_ grass CO_2_ responses^16^, supported by 24 years of additional research, we found that CO_2_ enrichment effects were larger under water limitation for aboveground biomass. This fertilization effect on shoot biomass was consistently stronger under water-limited conditions, although variable in size, across C_4_ subtypes, clades and regions (Extended Data Fig. 2).

**Extended Data Fig. 3:**
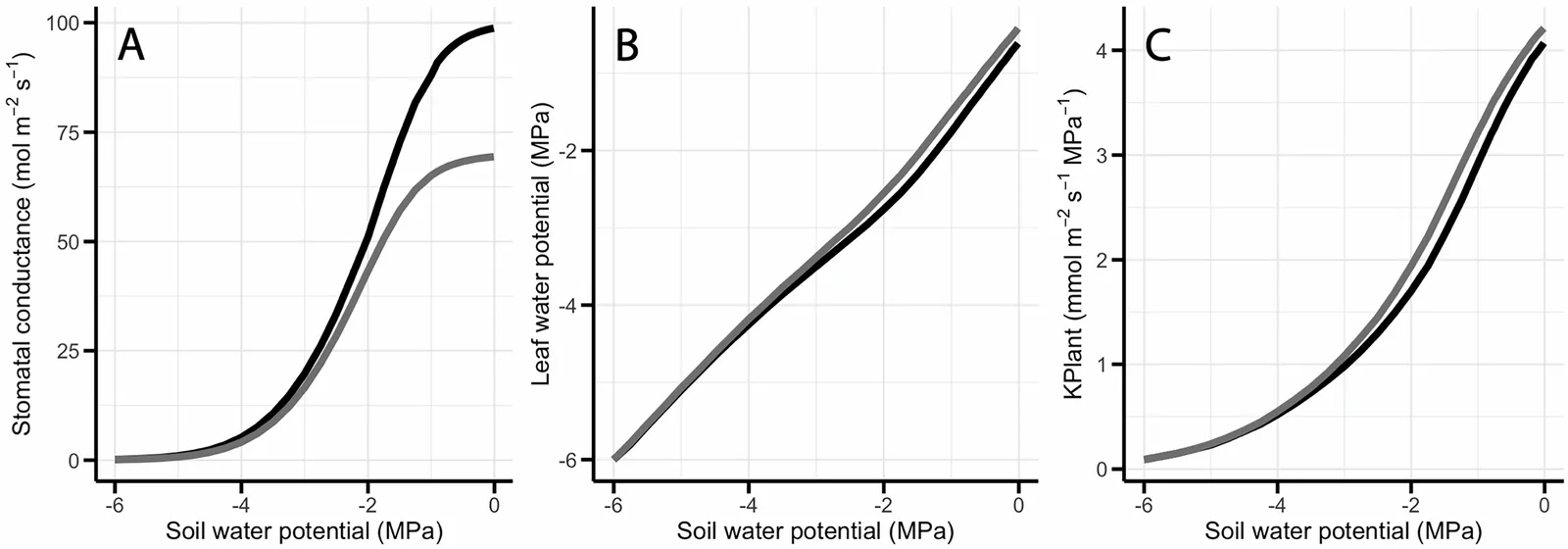
CO_2_ effects on modelled C_4_ grass hydraulics across a soil water potential gradient. Modelled stomatal conductance (A), leaf water potential (B) and plant conductance (K_plant_; C) responses of a typical C_4_ savanna grass (based on the species *Themeda triandra*) are shown under ambient CO_2_ (black lines) and elevated CO_2_ concentrations (grey lines).

As water limitation in experiments (due to a reduction of watering or rainfall) consistently amplified biomass responses to elevated levels of CO_2_, we next examined how CO_2_ altered C_4_ grass water use (Fig. 1b). CO_2_ enrichment decreased stomatal conductance in well-watered plants (logRR-CO_2_ = −0.40 (−0.60 to −0.26)) but not in water-limited plants (logRR-CO_2_ = −0.08 (−0.27 to 0.10); Fig. 1b,c), such that stomatal conductance decreased significantly under water limitation at ambient levels of CO_2_ but not at elevated levels of CO_2_ (Fig. 1c). Under water limitation, leaf water deficits were alleviated under elevated levels of CO_2_ (that is, higher midday leaf water potential; logRR-CO_2_ = −0.43 (−0.71 to −0.15); Fig. 1b,c). However, decreased transpiration under elevated levels of CO_2_ did not increase soil water content in water-limited treatments (logRR-CO_2_ = 0.08 (−0.10 to 0.25)). Modelling of C_4_ grass water relations under declining soil water (Fig. 1c and Extended Data Fig. 3) suggests that lower stomatal conductance under elevated levels of CO_2_ interacts with the hydraulic system to maintain leaf water potential. Together, the results of this experimental synthesis suggest that increased levels of CO_2_ substantially reduced water deficits in wild C_4_ grasses, which translated into photosynthetic gains under water limitation. As decreasing soil moisture and increasing drought frequency are likely outcomes of climate change arising from increasing levels of CO_2_ (refs. ^19,20^), these physiological interactions have large potential effects on the vegetation trajectories of grassy ecosystems in the future.

## Rising CO_2_ levels best explain field gains

**Fig. 2:**
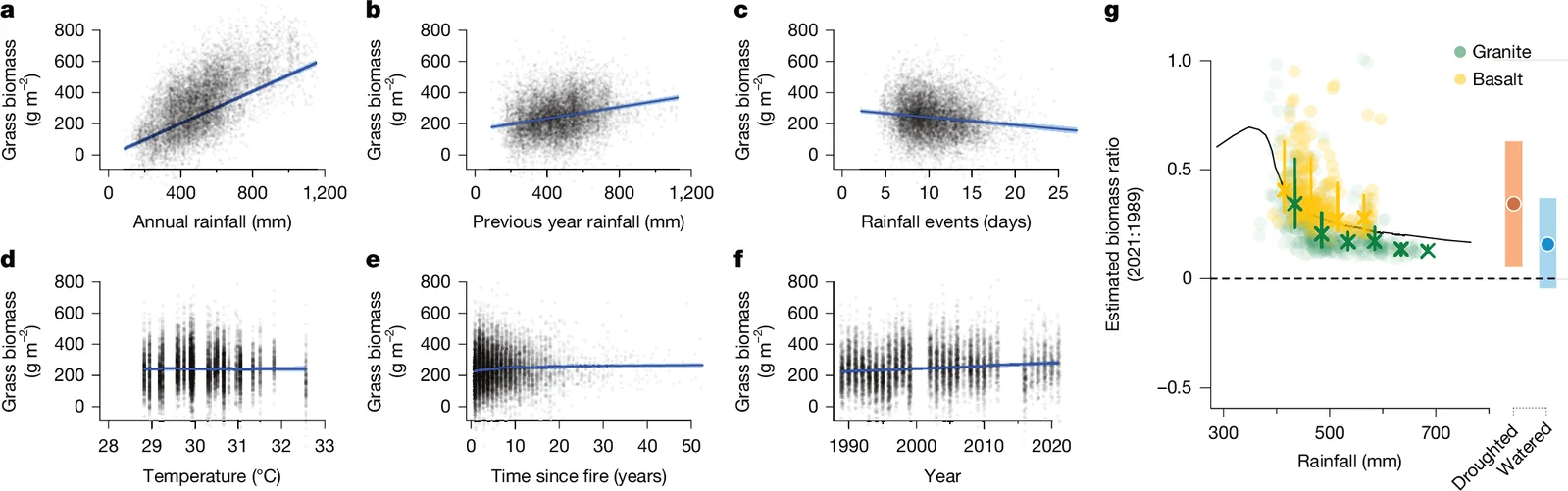
Partial dependence of grass biomass on climatic variation at monitoring sites in Kruger from 1989 to 2021. **a**–**g**, Grass biomass is associated with annual rainfall (**a**), previous year rainfall (**b**), the event distribution of rainfall (**c**), mean annual daily maximum temperature (**d**) and time since fire (**e**). After accounting for other sources of variation, grass biomass increased with time (**f**), with larger estimated response ratios in drier than in wetter savannas (**g**), shown alongside published FACE results^8,9^ (black line in panel **g**) and shoot biomass responses from Fig. 1 (bars on the right in panel **g**) plotted for reference; the dashed line indicates zero. The blue lines indicate model-predicted mean grass biomass, with all non-focal predictors held at their observed or time-averaged values as appropriate; the associated error bands show the central 68% interval of observed field estimates around the modelled mean (**a**–**f**). The crosses show median response ratios within 50-mm rainfall bins on each soil type, and the bars show central 68% intervals (**g**). Within each rainfall bin, sample sizes were 51 (400–450 mm), 90 (450–500 mm), 30 (500–550 mm) and 18 (550–600 mm) on basalts and 83 (400–450 mm), 102 (450–500 mm), 46 (500–550 mm), 71 (550–600 mm), 20 (600–650 mm) and 10 (650–700 mm) on granites. Response ratios were modelled using estimated annual grass biomass production in 1989 and 2021, allowing only year to vary and using time-averaged values of all other drivers for each plot with plot-level random effects. The lines (**a**–**f**) show best model fits (Supplementary Table 1), with grass biomass (in g m^−2^) = −7,253 + 3,973 (on granite) + (3.66 − 2.03 (on granite)) × year + 0.518 × annual rainfall + 0.183 × previous year annual rainfall − 5.02 × annual rainfall days − 0.717 × mean daily max temperature + 9.36 × log(time since fire) + random plot effects (Supplementary Table 2). The model shown here does not include any species-composition or trait-composition predictors. Panel **g** reproduced from ref. ^9^, John Wiley and Sons.

**Extended Data Fig. 4:**
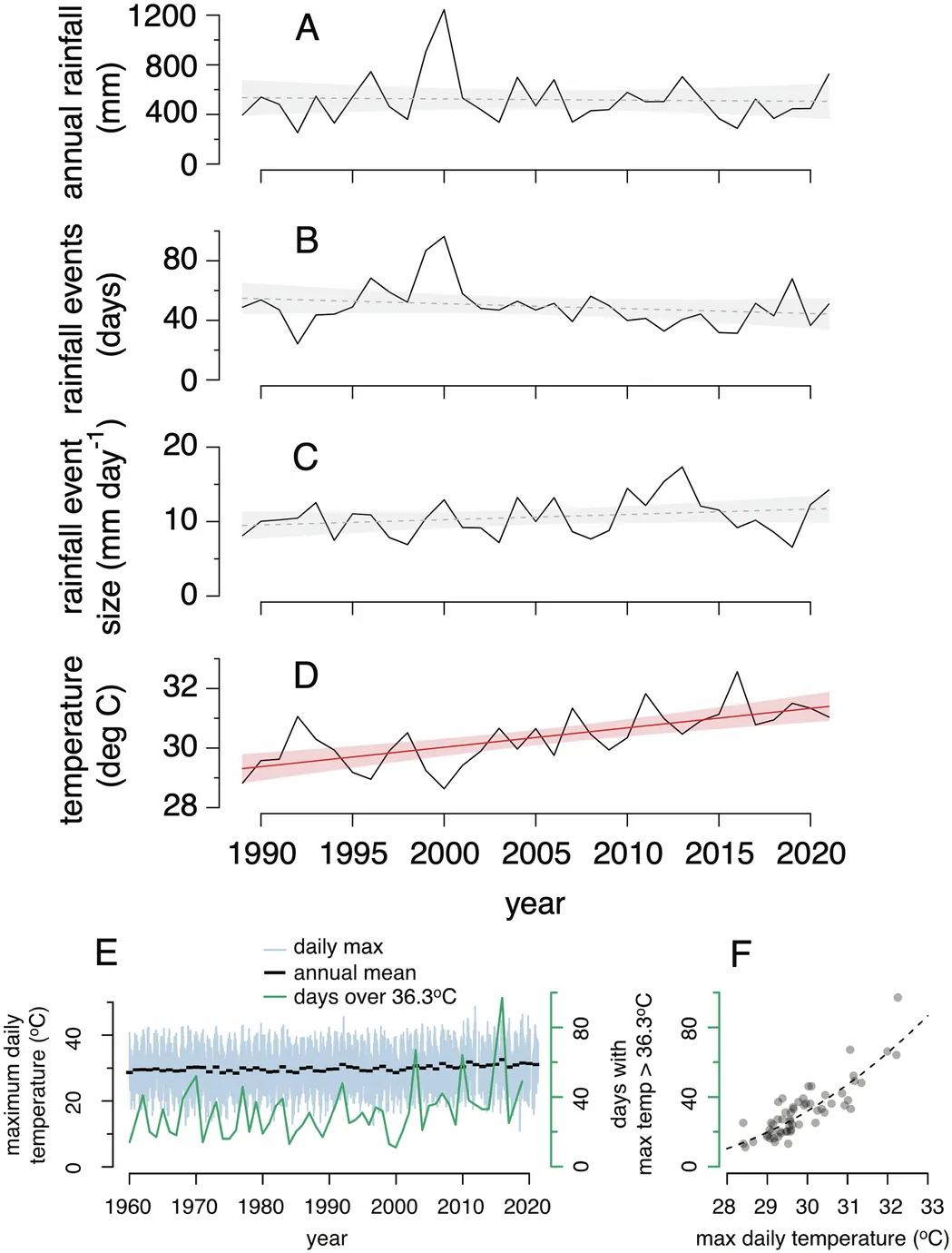
Climatological trends through time in Kruger National Park, South Africa. Rainfall climatology was estimated via records from 21 daily rainfall measurement stations throughout the park. Temperature was calculated as the annual mean of daily maximum temperature from one measurement station in Skukuza. Temporal trends were evaluated using linear regression in A-D. There has been no overall trend in rainfall climatology (flat trendlines) for annual rainfall (A), rainfall events (B), and rainfall event size (C) (shown in grey; *N* = 32, *p* > 0.05), whereas temperature (D) has increased (*temperature* = −100.5 [± 24.4] + 0.065 [± 0.012]* xyear*; *F* = 28.7, *N* = 32, *p* = 0.000009). Shaded bands indicate 95% confidence intervals around the fitted regression lines. Mean annual maximum daily temperature (E; black) was calculated as the July-June average of daily values for use in models for grass productivity. Daily observations (blue) that show a much wider variation (with values ranging from 11 °C to 48.8 °C). Mean annual maximum daily temperature is a reasonable proxy for the number of days annually with a maximum temperature exceeding the modelled temperature optimum for C_4_ productivity (36.3 °C (ref. ^173^)) (F), with a relationship described by *days*^0.5^ = −29.6 + 1.15 × *annual mean maximum daily temperature* (*R*^2^ = 0.729, *N* = 58, *p* < 0.001). Both mean annual temperature (see Supplementary Fig. 5) and days annually with max temperature exceeding 36.3 °C (*R*^2^ = 0.139, *N* = 58, *p* = 0.004) have increased through time.

Second, we examined the in situ responses of the C_4_ grass layer in a tropical savanna (Supplementary Fig. 1) to ambient changes in climate and atmospheric CO_2_ levels to determine whether experimental results translated into increases in grass biomass production over time as CO_2_ levels increased. Grass biomass varied spatially and temporally but increased overall across the 32-year observation period in Kruger (Supplementary Tables 1 and 2). Grass biomass increased for 2 years after wetter years (Fig. 2a,b and Supplementary Tables 1 and 2), reflecting the accumulation of grass biomass across years (Fig. 2e and Supplementary Tables 1 and 2, showing increased grass accumulation with time since fire), and perhaps also legacy effects of rainfall on biomass production from year to year^21^. Fewer rainfall events were also associated with greater grass biomass (Fig. 2c and Supplementary Tables 1 and 2), a relationship that arises because fewer events correspond to more rainfall per event (after controlling for annual rainfall). However, neither annual rainfall, the number of rainfall events, nor the mean rainfall event size changed directionally across the multi-decadal study period to explain the observed increase in grass biomass (Fig. 2f and Extended Data Fig. 4a–c). Conversely, high temperatures tended to reduce grass biomass (Fig. 2d and Supplementary Tables 1 and 2), yet a warming trend over the study period (Extended Data Fig. 4d), associated with more days exceeding the temperature optimum for C_4_ photosynthesis (Extended Data Fig. 4e), was insufficient to completely offset the overall biomass increase over the study period.

**Extended Data Fig. 5:**
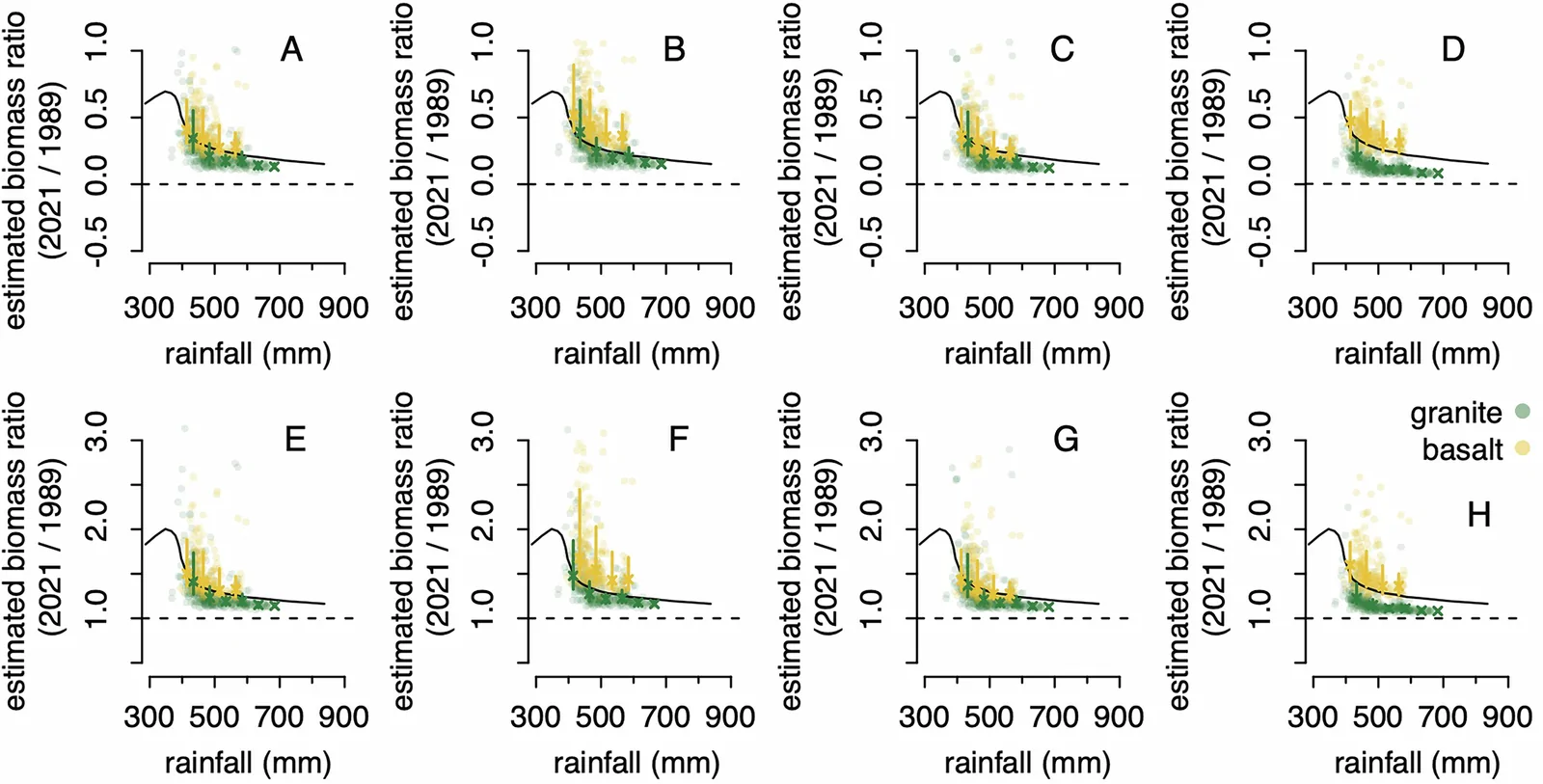
Grass biomass ratio responses to rainfall. The ratio of 2021 to 1989 estimated annual grass biomass across sites, shown in the top row as the natural log of the response ratio, and in the bottom row as an untransformed ratio. Crosses show median response ratios within 50 mm rainfall bins on each soil type, and bars show central 68% intervals. Within each rainfall bin, sample sizes were 51 (400–450 mm), 90 (450–500 mm), 30 (500–550 mm), and 18 (550–600 mm) on basalts and 83 (400–450 mm), 102 (450–500 mm), 46 (500–550 mm), 71 (550–600 mm), 20 (600–650 mm), and 10 (650–700 mm) on granites. The responses were estimated by controlling for year, geology, year x geology, rainfall, previous year rainfall, rainfall arrival rate, and temperature as fixed effects and plot as a random effect, without any community composition variables (A,E), excluding plots with > 20% relative abundance of lawn grass species (B,F), controlling for relative abundance of the five most common grass species in the park as fixed effects (C,G), and accounting for turnover in community-weighted means of SLA and maximum individual height as fixed effects (D,H; A repeats Fig. 2g,f from the main text). See Supplementary Table 3 for statistics corresponding to each of these models. For ratio calculations, rainfall climatology was modelled as constant through time. Black lines show predictions based on FACE experiment results of CO_2_-fertilization response from refs. ^8,9^. Soils derived from granite parent geologic material are sandy and nutrient-poor; soils on basalt parent geologic material are clay- and nutrient-rich. Grazing lawn grass species were *Urochloa mosambicensis, Digitaria eriantha*, and *Megathyrsus coloratum*; the five most common grass species were *Urochloa mosambicensis*, *Bothriochloa radicans*, *Digitaria eriantha*, *Themeda triandra*, and *Megathyrsus maximus*.

Changes in herbivory could not explain increases in C_4_ grass biomass in the field^22^. Overall herbivore density has increased in Kruger over the observation period^23^ (Supplementary Fig. 2), suggesting that grazing pressure has probably increased, which would, if anything, decrease grass standing biomass^22^. To further examine this possibility, we ran statistical models on the subset of plots with low relative abundance of herbivory-adapted lawn grasses. Subsetting the data in this way did not eliminate overall increases in grass biomass with time (Extended Data Fig. 5 and Supplementary Table 2), reinforcing the idea that decreased grazing is not the main driver of increases in biomass production through time.

Another potential explanation for increased grass biomass could be atmospheric nitrogen pollution from fossil fuel combustion. Globally, nitrogen deposition has alleviated nitrogen limitation to favour grass biomass production^24^, and nitrogen deposition from coal-fired power stations in South Africa has the potential to reach Kruger. However, nitrogen derived from coal carries a characteristic stable isotopic signature (δ^15^N), and an analysis of δ^15^N in herbarium specimens collected from Kruger over the late twentieth and twenty-first centuries shows no evidence of this signal^25^ (Supplementary Fig. 3). If anything, carbon-to-nitrogen ratios appear to have increased, consistent with progressive nitrogen limitation as grass biomass production has increased. Kruger may simply not experience high nitrogen deposition by global standards^24^.

After ruling out these alternatives, it appears likely that rising grass production in semi-arid Kruger savannas reflects a response to the 65 ppm increase in atmospheric CO_2_ levels during the past three decades^26^, as predicted under water-limited conditions in our experimental meta-analysis. Across all sites, grass biomass measured in the first year after fire increased by 75.1 g m^−2^ across 32 years (95% confidence interval (CI) of the mean 74.5–75.8 g m^−2^), representing a 28% increase in annual grass production. Although the absolute magnitude of plot-scale biomass increases was invariant to rainfall, drier savannas experienced larger relative biomass increases than wetter savannas (logRR-CO_2_ of 0.270 (95% CI 0.265–0.274) at 475 mm mean annual rainfall (MAR) versus 0.184 (0.178–0.190) at 625 mm MAR; Fig. 2g and Supplementary Tables 1 and 2), again consistent with predictions based on experiments of CO_2_ stimulation of photosynthesis in water-limited conditions.

## Soils and species shape community responses

Changes in grass biomass differed across the two dominant soil types in Kruger, with larger increases on nutrient-rich, clay soils derived from basalt than on nutrient-poor, sandy soils derived from granite (117.0 g m^−2^ (95% CI 108.3–125.7 g m^−2^) versus 52.0 g m^−2^ (95% CI 45.0–59.1 g m^−2^) in the first year after fire; logRR-CO_2_ = 0.33 (95% CI 0.28–0.37) versus 0.20 (95% CI 0.15–0.25); Fig. 2g and Extended Data Fig. 5). One possibility is that the fertility of the basalt-derived clays in Kruger may release grasses from soil nutrient limitation in the face of CO_2_ fertilization^27^. Alternatively, soil texture may change plant available moisture via differences in field capacity and wilting point. In a field experiment in a prairie ecosystem, grass biomass on clay soils, with higher field capacity, showed a larger CO_2_ response than on sandy soils^28^ (but see also ref. ^29^).

Next, we considered potential contributions of shifts in species abundance within communities to observed biomass increases, as past work has shown that long-term experimental exposure of mixed C_3_–C_4_ prairies in North America to CO_2_ enrichment can cause community turnover^29^. To account for functional and composition turnover in Kruger, we incorporated data on abundance and productivity-related traits (specific leaf area and plant height) of the five most common grass species in the park. Taller, more productive grasses (for example, *Megathyrsus maximus*) were increasingly dominant through time, a process which in itself may be partly determined by rising levels of CO_2_ (ref. ^29^). Statistically accounting for turnover in species and functional traits reduced, but did not eliminate, the observed grass biomass increase (to 66.7 g m^−2^ (95% CI 66.0–67.4 g m^−2^) controlling for species composition and 63.2 g m^−2^ (95% CI 62.2–64.2 g m^−2^) controlling for functional turnover in the first year after fire; Extended Data Fig. 5 and Supplementary Table 3).

**Fig. 3:**
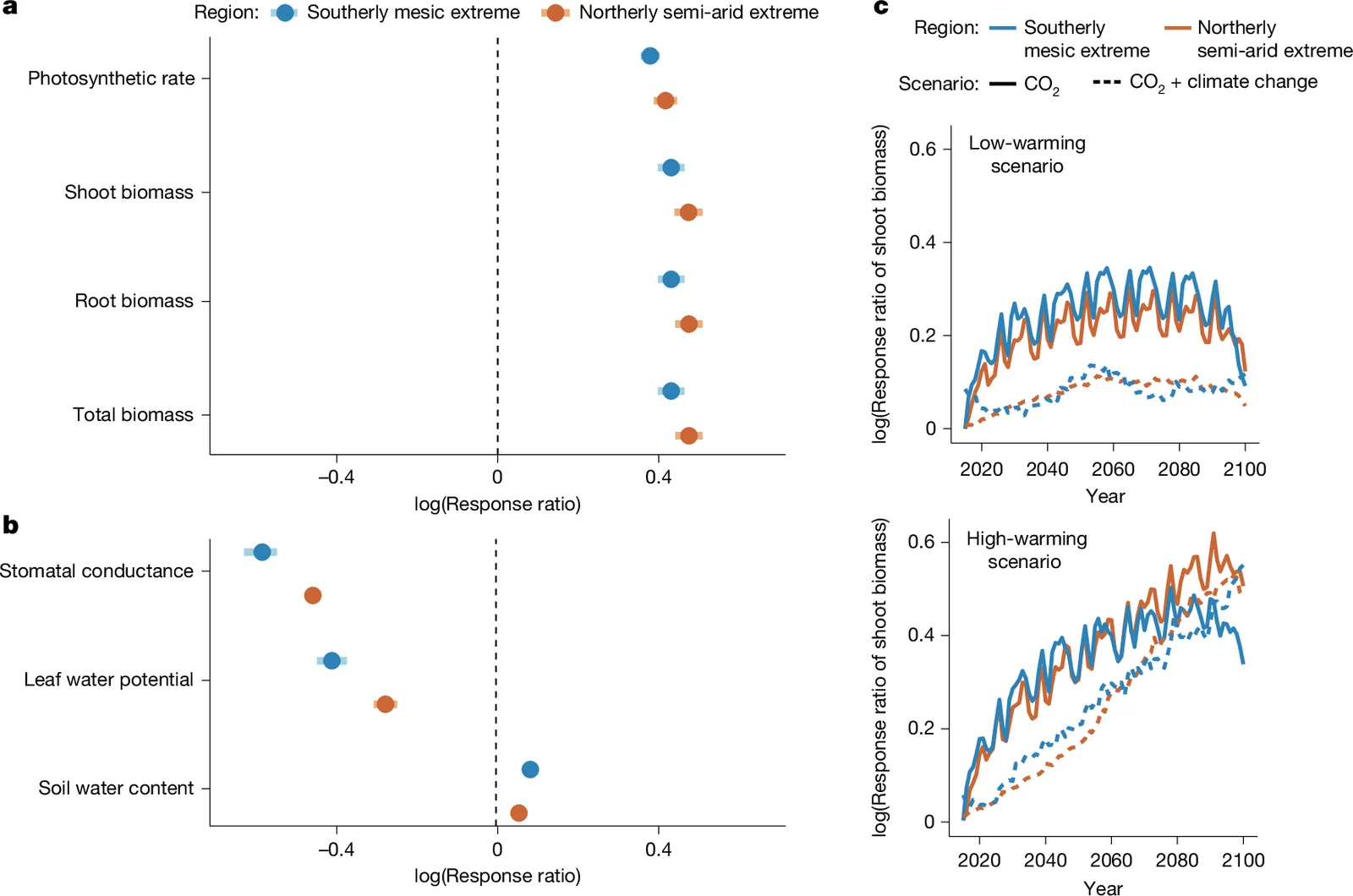
Modelled increases in C_4_ grass photosynthesis, biomass and water savings modelled for future increasing CO_2_ scenarios are reduced but not eliminated by climate change. **a**,**b**, Using a global vegetation model (CLM5), grass-trait values were predicted for two C_4_-grass-dominated regions at the northerly semi-arid and southerly mesic extremes of Kruger under a high-warming scenario. The responses of grass photosynthesis and biomass (**a**) and water relations (**b**) to CO_2_ concentrations in the year 2075 relative to those today are displayed as logRRs. The error bars represent 1 s.d. around the mean response ratio (*n* = 11), averaged over the decade 2070–2080. The year 2075 was selected because the CO_2_ concentration predicted for 2075 under this high-warming scenario (approximately 690 ppm) is similar to the average CO_2_ concentration for elevated CO_2_ treatments (700 ppm) from our literature synthesis of C_4_ grass responses to increasing levels of CO_2_. **c**, The modelled effects of climate change on the CO_2_ fertilization of aboveground C_4_ grass biomass at the two sites, under both a low-warming and a high-warming scenario, are shown as a time series of logRRs for 2015–2100. Each time series displays the 10-year running average, with a forecasting pad applied beyond 2100 to avoid an end-of-series artefact.

Overall, the potential effect size of CO_2_ fertilization in the field was smaller than water-limited CO_2_ responses in the meta-analysis (Fig. 2g). On the one hand, this is expected as experimental treatments included much larger differences in CO_2_ (180–400 ppm over ambient levels compared with an increase of only 65 ppm across 32 years in Kruger); on the other hand, a saturating response to future increases in CO_2_ levels is anticipated due to non-linear relationships between plant photosynthesis and atmospheric CO_2_ (although the point of saturation may differ depending on, for example, soil nutrient availability^29^). A more informative direct comparison can be made with a North American experiment that provided a continuous CO_2_ gradient of 200–500 ppm to four mixed C_3_–C_4_ prairie communities on three different soil types, yielding an increase of 38–58 g m^−2^ of aboveground annual net primary productivity (ANPP; 95% CI 26–62 g m^−2^)^29^. For the equivalent CO_2_ level increase on sandy versus clay-rich soils, 52 and 117 g m^−2^ of grass biomass, respectively, accumulated in the year after fire in Kruger (a reasonable estimate for ANPP), which overlapped but exceeded the range observed across experimental prairie communities on similar variation in soil texture at similar rainfall^29^. Direct comparisons can also be made with trends inferred from satellite observations for C_4_-dominated ecosystems^1^, which, scaled to the full 32-year span of our dataset, yielded a closer match to our estimates, at 80 g m^−2^ of ANPP (95% CI 3–175 g m^−2^; Methods).

## Simulations capture biomass trends

**Extended Data Fig. 6:**
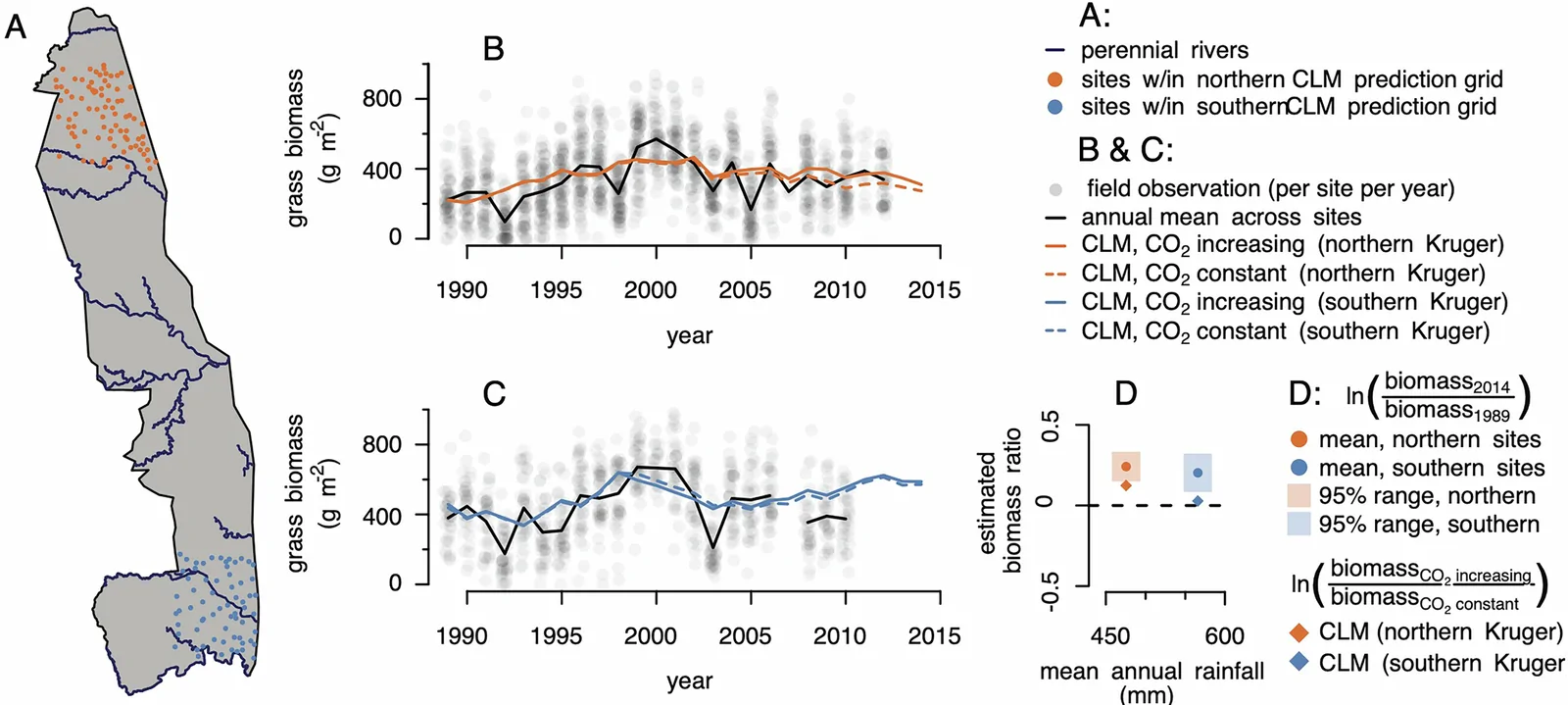
Comparison of observed grass biomass with CLM5 model predictions of historical C_4_ grass biomass across the time period 1989–2014. Panal A shows a map of site locations included in validation for each model grid square. Panels B and C show time series of aboveground grass biomass in observations and global model predictions for the north (B) and south (C) of Kruger (with either CO_2_ increasing or held at 1989 levels), and comparison of predicted CO_2_ responses from CLM5 versus statistical modelling of observed biomass from northern and southern regions of Kruger, shown as the log-response ratio (D). For panel D, log-response ratios were calculated from model-predicted biomass under increasing versus fixed 1989 CO_2_; no separate statistical hypothesis test was performed for this comparison. CLM5, forced with real climate observations, closely matched mean observed grass biomass, as assessed by linear regression, in both north/semi-arid (R^2^ = 0.354, N = 24, p = 0.002; grass_observed_ = 1.09 [ ± 0.31] ⋅ grass_model_ – 308 [± 1081]) and south/mesic (R^2^ = 0.393, N = 21, p = 0.002; grass_observed_ = 0.85 [± 0.24] ⋅ grass_model_ + 141 [± 1232]) test regions. GIS data and layers in panel A were supplied by South African National Parks (SANParks) and used with permission under a SANParks data user agreement for research purposes.

To evaluate how these processes could influence future biosphere responses to climate change, we modelled C_4_ grass responses to rising atmospheric levels of CO_2_ and changes in climate using CLM version 5 (CLM5)^6^. CLM5 represents CO_2_ responses of C_4_ grasses through two mechanisms. First, C_4_ photosynthesis shows a sharply saturating response to intercellular levels of CO_2_ (ref. ^30^), well below the maximum values of CO_2_ concentrations included in this study, although this CO_2_ response depends strongly on plant water status^31^. Second, stomatal conductance increases with photosynthesis, but decreases in direct response to CO_2_ and atmospheric vapour pressure deficit^6,32,33^ We focused our modelling on two test sites, one centred in the northerly semi-arid region of Kruger (with less than 500 mm MAP)^34^ and the other in the southerly mesic region of Kruger (500 mm or more MAP^34^) up to the year 2100 (Extended Data Fig. 6). Simulations prescribed the plant functional type in each grid cell as entirely C_4_ grass, to avoid the complexities introduced by competition and succession by other plant functional types. We first evaluated the match between observations of C_4_ grass biomass variation in the Kruger field data and model predictions over the ‘historical’ model period between 1989 and 2014 (Extended Data Fig. 6). Model predictions of past grass biomass agreed well with the mean annual observed grass biomass in both semi-arid (*R*^2^ = 0.354, *n* = 24, *P* = 0.002; grass_observed_ = 1.09 (±0.31) × grass_model_ − 308 (±1,081)) and mesic (*R*^2^ = 0.393, *n* = 21, *P* = 0.002; grass_observed_ = 0.85 (±0.24) × grass_model_ + 141 (±1,232)) test regions. In addition, comparing historical model runs with increasing versus constant CO_2_ showed CO_2_ fertilization effects that were within the 95% CI of estimates from field observations (Extended Data Fig. 6). Together, these lend confidence in the ability of the model to capture ecosystem-level productivity variation.

## Climate dampens future CO_2_ fertilization

**Extended Data Fig. 7:**
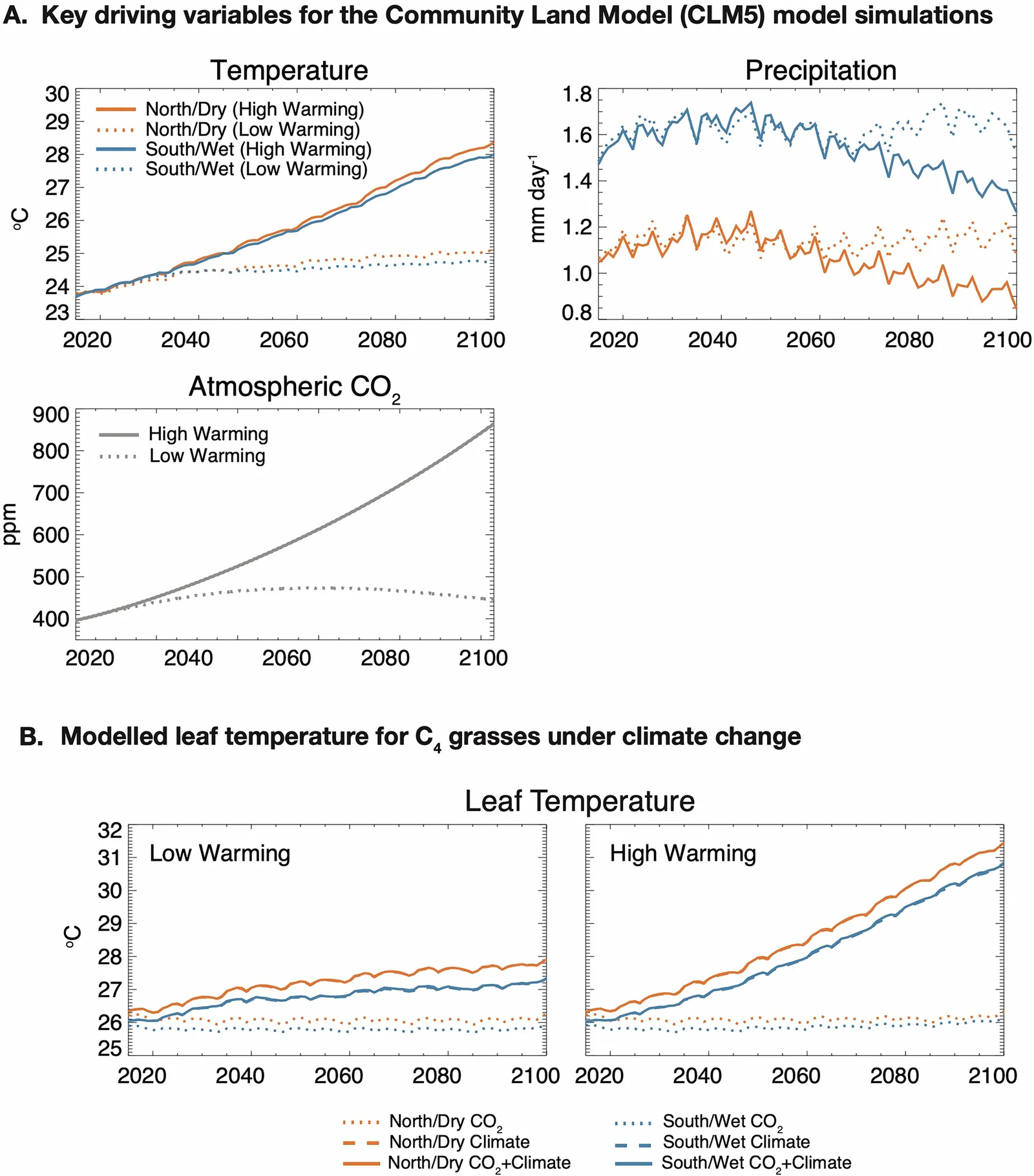
Key driving variables for the Community Land Model version 5 (CLM5) model simulations (A) and modelled leaf temperatures (B). Shown in panel A are averaged ground temperatures (°C), precipitation (mm day^−1^) and atmospheric CO_2_ levels (ppm) from 2015 to 2100 for two regions in Kruger National Park (a drier, semi-arid northern region (‘North/Dry’) and a wetter, mesic southern region (‘South/Wet’)) under low- (SSP1-2.6) and high-warming (SSP3-7.0) scenarios. Shown in panel B areannually averaged leaf temperature (°C) from 2015 to 2100 for two regions in Kruger National Park (a drier northern region and a wetter southern region) under a low and high warming scenario. Each driver of change (CO_2_ and climate change) is evaluated individually and in combination. Dashed lines (‘Climate only’ run) are overlain by solid lines (‘CO_2_ and Climate’ run).

**Extended Data Fig. 8:**
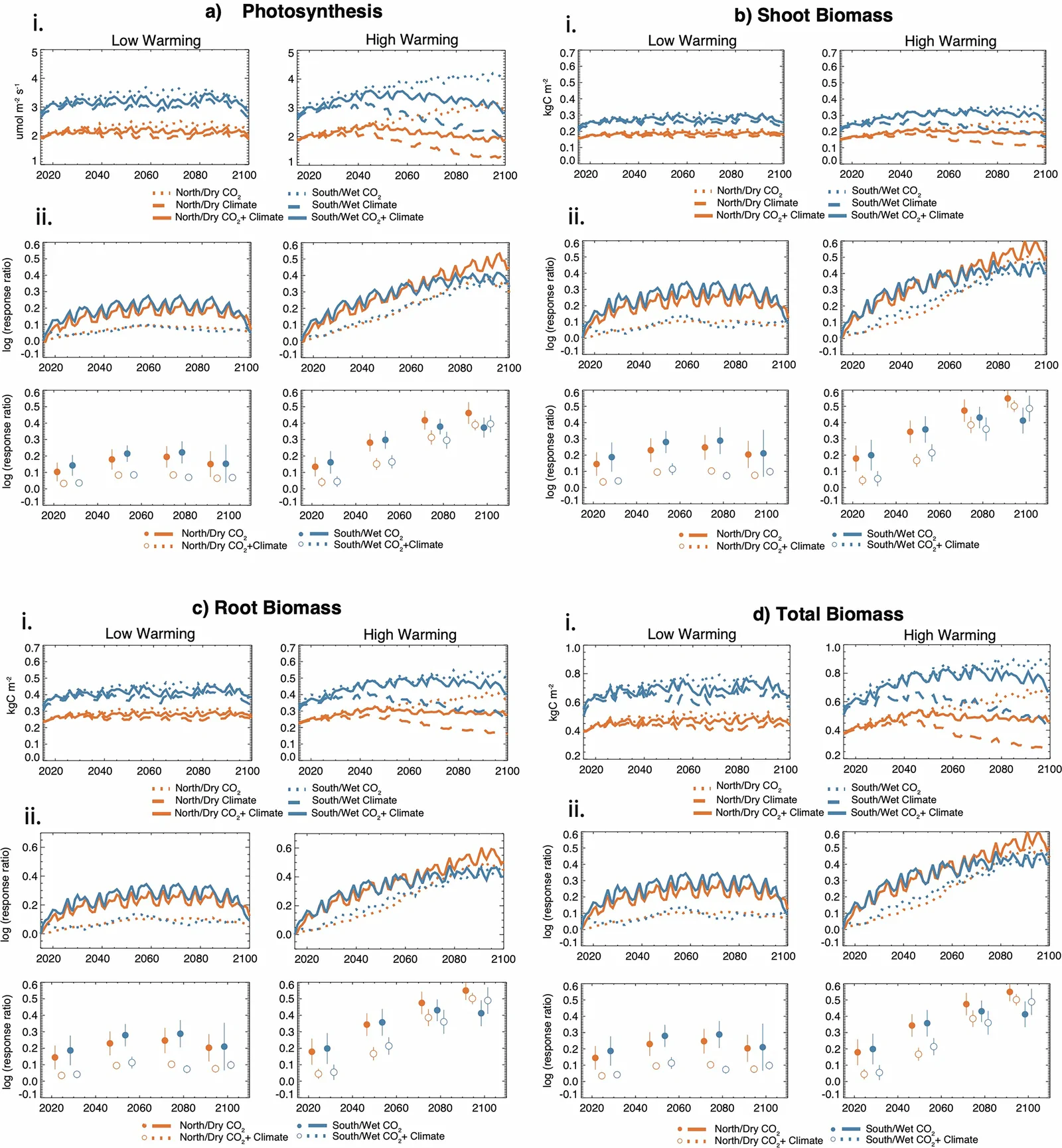
Modelled photosynthesis and biomass for C_4_-grass-dominated savanna sites under future climate change scenarios. Modelled time series of photosynthesis (a), shoot biomass (b), root biomass (c) and total biomass (d) until 2100 for two regions in Kruger National Park (a drier northern region (‘North/Dry’) and a wetter southern region (‘South/Wet’)) under low and high warming scenarios are shown (subpanels i). Each driver of change (CO_2_ and climate change) is evaluated individually and in combination. The influence of CO_2_ is also displayed as a log response ratio time series and as binned 10-year averages (subpanels ii). Each time series displays the 10-year running average, with a forecasting pad applied beyond 2100 to avoid end-of-series artefacts. Bars show the standard deviation of each 10-year binned values, with n = 10 annual values per bar.

**Extended Data Fig. 9:**
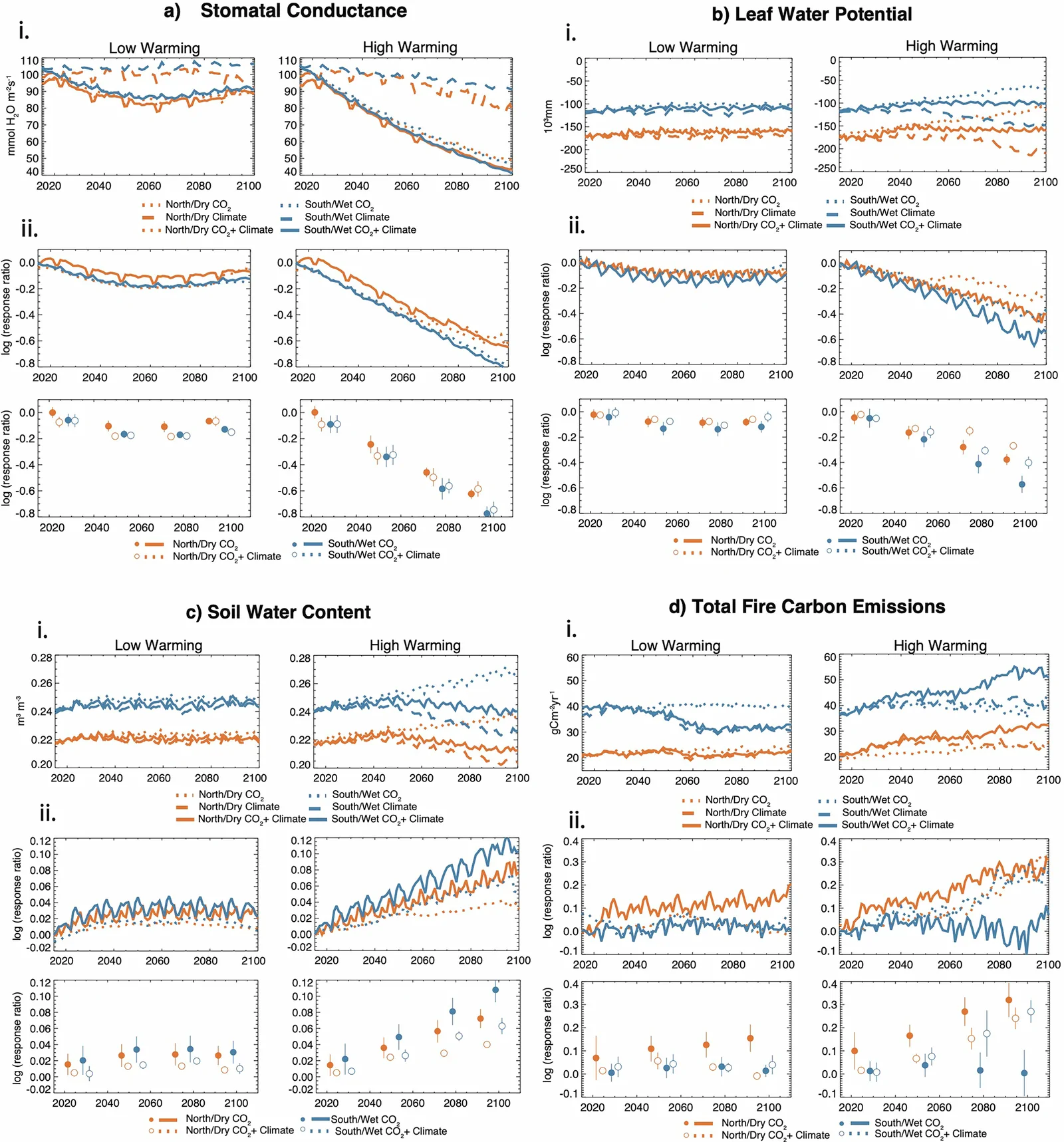
Modelled water-relation traits, soil moisture and fire emissions for C_4_-grass-dominated savanna sites under future climate change scenarios. Modelled time series of stomatal conductance (A), leaf water potential (B), soil water content (C) and total fire carbon emissions (D) until 2100 for two regions in Kruger National Park (a drier northern region (‘North/Dry’) and a wetter southern region (‘South/Wet’)) under low and high warming scenarios are shown (i. subpanels). Each driver of change (CO_2_ and climate change) is evaluated individually and in combination. The influence of CO_2_ is also displayed as a log response ratio time series and as binned 10-year averages (ii. subpanels). Each time series displays the 10-year running average, with a forecasting pad applied beyond 2100 to avoid end-of-series artefacts. Bars show the standard deviation of each 10-year binned values, with n = 10 annual values per bar.

We next used CLM5 to predict future ecosystem trajectories. We evaluated future CO_2_ effects against experimental meta-analysis results, factorially switching each driver off to isolate the modelled effects of CO_2_ versus climate change, focusing on CO_2_-only runs for experimental validation (Extended Data Fig. 7a). Under future elevated levels of CO_2_, modelled photosynthesis and biomass production increased (see Fig. 3a for SSP3-7.0; Extended Data Fig. 8a–d). The magnitude of increase was larger at both sites than the experimental results but particularly at the semi-arid site, consistent with our findings that water limitation drives CO_2_ fertilization of photosynthesis and aboveground biomass (Fig. 3a; RR_north/semi-arid_ = 0.42 and RR_south/mesic_ = 0.38 for photosynthesis; RR_north/semi-arid_ = 0.47 and RR_south/mesic_ = 0.43 for aboveground biomass across approximately 700 ppm of CO_2_ under SSP3-7.0 from 2015–2075). The model predicted a strong increase in root biomass in response to increasing levels of CO_2_, which contrasts with the highly variable response of C_4_ grass root biomass to elevated levels of CO_2_ found in our experimental synthesis and in grassland FACE experiments (for example, logRRs ranging from −0.4 to +0.4 in ref. ^35^; Fig. 3a and Extended Data Fig. 8c), suggesting that the model overestimates future grass root biomass and associated soil carbon storage. Modelled effects of CO_2_ on plant water status (Fig. 3b and Extended Data Fig. 9a–c) suggest contrasts with both our synthesis (Fig. 1b,c) and with FACE experiments^36^, predicting consistently reduced stomatal conductance instead of the observed sustained high stomatal conductance under drought with CO_2_ enrichment. The difference implies that refinements to the CLM representation of stomatal and hydraulic regulation under elevated levels of CO_2_ and water limitation on productivity may be beneficial, particularly as this is an active area of physiological research^37,38^.

However, overall, comparison with both historical observations and experimental meta-analysis suggests that CLM5 is able to phenomenologically capture C_4_ grass responses to CO_2_, indicating that model projections could be informative of future C_4_ grass production. Simulations showed a clear continued increase in C_4_ grass production under increased levels of CO_2_ but also showed that climate change reduced, but did not eliminate, CO_2_ fertilization of C_4_ grass biomass into the future (Fig. 3c and Extended Data Fig. 8b). Modelled climate effects—showing lower levels of photosynthesis and productivity with higher air temperature and lower levels of rainfall—are consistent with observations from Kruger field data (Fig. 2d). In the model, these outcomes resulted from lower stomatal conductance and associated higher leaf temperature (Extended Data Fig. 7b), which depresses photosynthetic capacity once it exceeds 36.3 °C (Supplementary Fig. 4). This model response concords with past work showing that photosynthesis in C_4_ grasses declines at such high temperatures through a number of mechanisms^39–42^. The combination of CO_2_ and climate effects on productivity was larger in the high-warming (SSP3-7.0) than in the low-warming (SSP1-2.6) scenario, suggesting that temperature suppresses productivity but not enough to eliminate CO_2_ fertilization (Fig. 3c). Together, the combination of rising CO_2_ levels and climate change is projected to result in increases in C_4_ grass production of between 0.01 and 0.05 kg C m^−2^ by 2100 (representing an increase of 6–21% from 2015 levels; Extended Data Fig. 8b).

## Fire impacts remain uncertain

**Extended Data Fig. 10:**
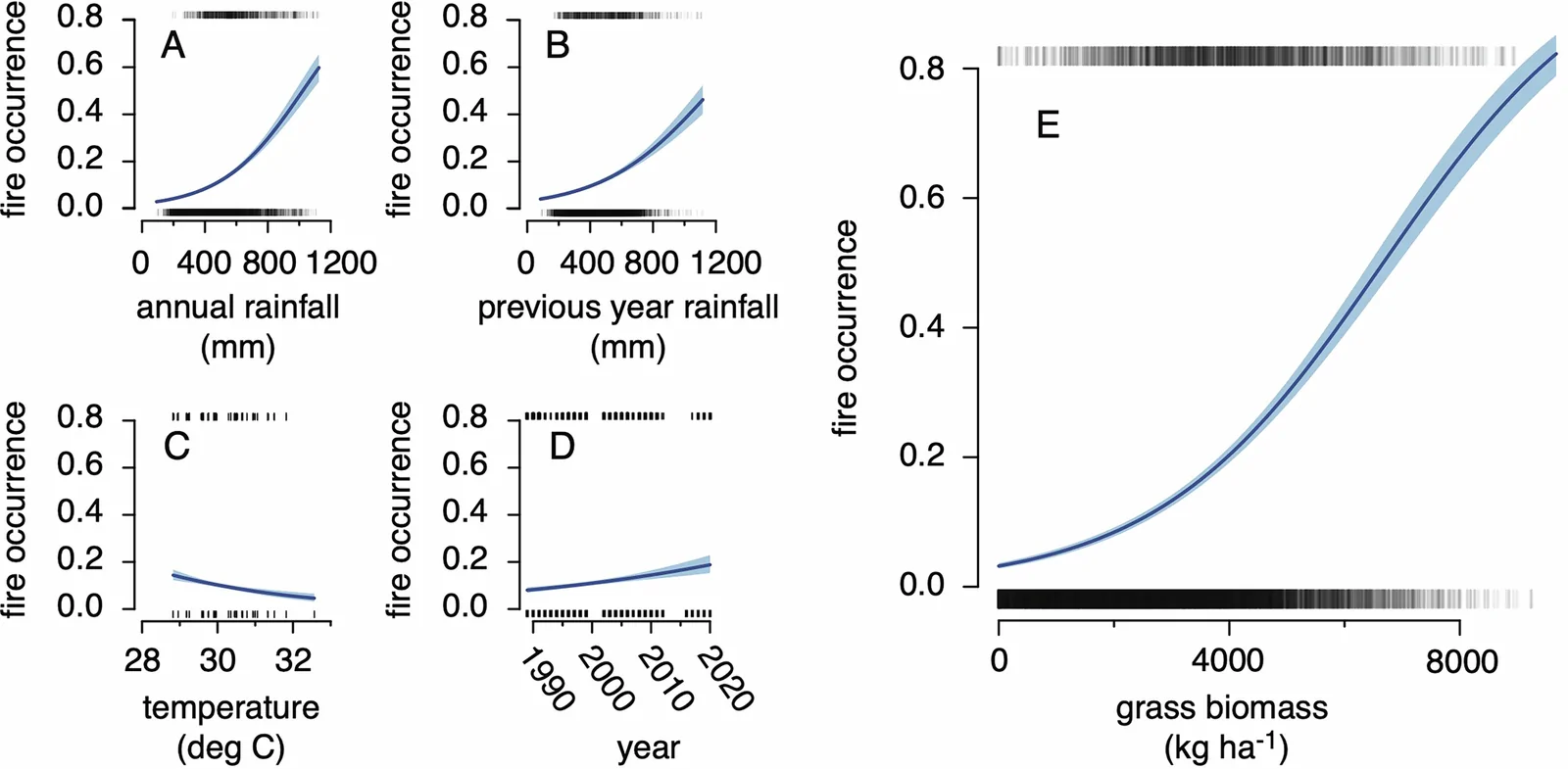
Partial dependence of fire occurrence at monitoring sites in Kruger National Park, South Africa, from 1989 to 2020. Panels A-D are derived from a single logistic regression including climatological variables through time, and panel E is derived from a separate logistic regression including grass biomass through time. Annual rainfall (A) and previous year rainfall (B) tended to increase fire occurrence, while temperature (C) tended to decrease fire occurrence. Fire occurrence also slightly increased through time, from a predicted probability of 0.12 in 1989 to 0.17 in 2020 (D). These effects are likely due to increasing grass biomass, since fire occurrence consistently increased with grass biomass through time (E). Grey bands at the top and bottom of each plot show observations of individual fire occurrences. Blue lines show the model-predicted mean probability of fire occurrence, with blue bands indicating 95% confidence intervals around the predicted mean. Model selection results are shown in Supplementary Table 4 and full model results in the first row of Supplementary Table 5.

Finally, we examined whether CO_2_ fertilization of grass affected an important downstream ecological process: fire. As fire activity is fuel limited in many savannas^14^, CO_2_ fertilization of C_4_ grass biomass could increase fire extent^43^. Consistent with this prediction, in field observations, we found multi-decadal increases in fire occurrence in Kruger associated with increasing grass biomass, although trends were small and statistically weak (Extended Data Fig. 10 and Supplementary Tables 4 and 5). In CLM5 simulations, future fire depended on whether fire regimes were limited by fuel amount (which increased with CO_2_ fertilization at the semi-arid site) or fuel moisture (at the mesic site; Extended Data Fig. 9d). Climate change increased fire activity at the mesic site (due to declining biomass moisture) but damped CO_2_-driven fire increases at the semi-arid site (by decreasing grass biomass). This switch from fuel-amount to fuel-moisture limitation has been observed^14^ at higher levels of rainfall than modelled, which suggests that models should be refined to decrease their sensitivity to fuel moisture in savannas. Together, model results suggest that CO_2_ effects on future grass-fuelled savanna fire occurrence could be mediated by plant water relations (see Supplementary Discussion on fire observations).

## Implications for savanna carbon

In summary, across 70 experiments and 32 years of in situ field observations, we found consistent evidence that higher CO_2_ levels increase C_4_ grass biomass production under water limitation. In experimental settings, increased productivity was linked to decreased water use and improved plant water status. This suggests that the CO_2_ fertilization that we observed in the field is principally driven by reduced plant water deficits, which could extend growth following rainfall events and into the dry season^44^, rather than by a direct stimulation of Rubisco activity. CLM5 simulations suggest that observed productivity increases in situ could continue through the twenty-first century, exceeding suppressive effects of increasing temperatures and aridity.

Overlooked C_4_ grass CO_2_ fertilization has major implications for savanna ecosystem function. In forests, CO_2_ fertilization has been linked to carbon storage^4^. In C_4_ grasslands, carbon is stored long term primarily in soil and in coarse woody roots^45,46^, as aboveground carbon is vulnerable to herbivory and fire^47^. Most soil organic carbon is derived from grass root litter^48^, which did not increase in experiments, suggesting that responses of soil organic carbon due to CO_2_ effects on grasses could be negligible. However, CO_2_ fertilization could alter forage quality (higher carbon-to-nitrogen ratio and lower protein levels)^49^, changing animal consumption, litter decomposability and conversion to recalcitrant soil carbon. CO_2_ fertilization could also change dynamics of tree–grass interactions, amplifying C_3_ woody plant encroachment and losses of C_4_ grass cover^50^. Interactions with soil water and fire^6^ make this difficult to predict, but stronger CO_2_ fertilization of C_4_ grasses in drier savannas could help to explain their slower rates of woody plant encroachment^51^. Together, these potential mechanisms result in highly uncertain responses of savanna carbon to increasing CO_2_, making them a prime target for future work. Overall, these results combine with past work to demonstrate CO_2_ fertilization in both trees and grasses across the tropics^4^ and to highlight the importance of direct CO_2_ effects on past and future vegetation trajectories.

## Methods

### Meta-analysis

#### Search methodology

Suitable studies were identified through a systematic literature search. To create a comprehensive search string, a naive Boolean search string of (‘elevated CO_2_’ or ‘high CO_2_’) and ‘C_4_’ and (‘grass*’ or ‘Poaceae’) was used initially in ISI Web of Science, resulting in 186 results (4 March 2021). The citation record and abstracts of all results were then exported for keyword co-occurrence network analysis using the R package Litsearchr^52^. This generated the final Boolean search string of (‘C_4_’ or ‘C-4’) and (‘grass*’) and (‘elevat*’ or ‘increas*’ or ‘enrich*’) and (‘carbon dioxide’ or ‘CO_2_’) and (‘biomass’ or ‘growth’ or ‘product*’ or ‘respons*’).

The final search terms were used in ISI Web of Science and SCOPUS, resulting in 768 unique results (March 2021). Google Scholar was not used because it does not recognize wildcard components (*) and a search without these components produced too many results (more than 36,000). Several e-theses repositories (White Rose repository, British Library EThOS and South African ETD Collections) were also queried, resulting in two further suitable studies.

#### Inclusion and exclusion criteria

The studies were screened by title and abstract and were selected if they investigated the responses of wild, C_4_ grass species of grass-dominated ecosystems (grasslands and savannas) to elevated [CO_2_]. Therefore, studies on non-grass, C_3_ and domesticated species were excluded, as well as those from other ecosystems (for example, agricultural weeds and wetland species such as *Spartina patens*). We analysed data using a phylogenetically controlled method (see the ‘Modelling’ section below) so responses needed to be identified to the species level. Therefore, studies that did not isolate responses of particular species (for example, grassland community responses) were excluded.

For inclusion, studies had to include both ambient [CO_2_] (aCO_2_) and elevated [CO_2_] (eCO_2_) treatments. A range of [CO_2_] has been used in both treatments, reflecting increasing atmospheric [CO_2_] during the approximately 40-year timeframe of these studies and changing predictions of near-future [CO_2_]. Ambient treatments used [CO_2_] less than or equal to 450 ppm. [eCO_2_] treatments ranged from 460 to 1,005 ppm. To investigate how C_4_ grass responses to eCO_2_ interacted with plant water status, studies that also included treatments relating to water availability (in addition to [CO_2_] treatments) were included.

Studies had to report at least one of the following metrics: aboveground biomass, belowground biomass, total biomass, stomatal conductance measured at the growth [CO_2_] (*G*_*s*_) or light-saturated net CO_2_ assimilation rates measured at the growth [CO_2_] (*A*).

#### Data extraction

Grass responses at aCO_2_ or eCO_2_ were extracted either from the main body of the text, tables or Supplementary Information, or manually digitized from figures using WebPlotDigitizer^53^. When data were not available, we contacted authors directly to request data.

For each species within each study, we extracted the following information: the two [CO_2_] values, the mean value of the variable of interest (biomass, *G*_*s*_ or *A*) under aCO_2_ and eCO_2_, the sample size and the standard error (s.e.; either as presented or calculated from the standard deviation and sample size). Occasionally, when multiple data points were reported for one species in a study (for example, measurements were taken over several years), data were recorded separately but linked by a study identification number.

In studies in which both CO_2_ and water availability treatments were applied, the CO_2_ response was collected separately by water treatment level. ‘Low’ water availability treatments involved a reduction, relative to ‘high’ water availability treatments, in the amount of water supplied to plants, either due to the restriction or withholding of supplementary watering, an experimental reduction of rainfall (by using rain-out shelters, for example), or the occurrence of a low-rainfall event (for example, a growing season with below-average rainfall). If additional environmental stresses (such as salinity or ozone treatments) were imposed factorially, only the CO_2_ response at the ambient, non-stressful level of this other factor was used.

To account for other factors that could influence grass responses to eCO_2_, metadata describing the experimental methodology of each study were recorded. These included pot size (categorized as less than 10 l pots, more than 10 l pots or if the plants were in the ground), the length of exposure to CO_2_ treatments (from treatment initiation to plant trait measurement; categorized as less than 60 days, 60–120 days or more than 120 days), and the exposure methodology. The latter consisted of four categories: FACE, open-top chamber, indoor-controlled environment growth chamber or outdoor enclosed greenhouse.

The final dataset for analysis consisted of 655 pairs of observations under aCO_2_ and eCO_2_, of which 550 did not limit water availability and 105 were droughted, on 82 C_4_ grass species from 70 studies^43,54–122^. The species represented 42 out of the 314 genera containing C_4_ grass species, all 4 grass subfamilies where C_4_ photosynthesis has evolved (Aristoideae, Chloridoideae, Micrairoideae and Panicoideae), and the 3 C_4_ subtypes (NAD-ME, NADP-ME and PCK). These species are found across broad temperature and rainfall ranges, covering a significant proportion of climatic space from temperate to tropical biomes (Supplementary Fig. 5a). The dataset had global coverage, with data from C_4_ grass species from Africa (15 studies), Asia (4 studies), North America (34 studies), Oceania (9 studies) and South America (8 studies). Twelve of these 70 studies were also included in a previous analysis by Wand et al.^16^ of C_3_ and C_4_ grass responses to eCO_2_ (refs. ^70,72,75,88,90,99,106,110,113,117,118,122^). The other 51 studies utilized in this previous meta-analysis^16^ were not included here because they investigated C_3_ (33 studies) or non-grassland species (wetland or agricultural land species; 7 studies), did not report any variables of interest here (for example, a focus on leaf traits; 6 studies), reported community level responses (2 studies) or were not accessible (3 studies).

#### Effect size calculations

Log-transformed response ratios (RR) were used to calculate the size of CO_2_ effects on grass traits, a common approach in ecological meta-analyses when comparing a control and a treatment group^123^. RRs were calculated as:

$$\mathrm{RR}=\mathrm{ln}\left(\frac{{\overline{x}}_{T}}{{\overline{x}}_{C}}\right)$$

where ${\overline{x}}_{T}$ is the measured value for the treatment group (the eCO_2_ group) and ${\overline{x}}_{C}$ is the measured value for the control group (the aCO_2_ group). A negative RR (RR < 0) represents a decrease in the measured variable under eCO_2_ (relative to aCO_2_), and a positive RR (RR > 0) represents an increase in the measured variable under eCO_2_.

s.e. values were imputed for the small number of studies that did not report s.e. or standard deviations. Missing values were imputed using Bracken’s approach^124^, which uses the mean variable value and an average s.e.-to-mean ratio for that CO_2_ treatment.

When pooling RR from multiple studies, each RR should be weighted by its precision to give less weight to studies with high s.e. and vice versa. Weights were calculated as inversely proportional to the sampling variance of a RR observation^123^ as

$$\mathrm{s.e.}(\mathrm{RR})=\sqrt{\frac{{\mathrm{s.e.}}_{C}^{2}}{{\overline{x}}_{C}^{2}}+\frac{{\mathrm{s.e.}}_{T}^{2}}{{\overline{x}}_{T}^{2}}}$$

where s.e._*C*_ and s.e._*T*_ are the s.e. for the control (aCO_2_) and treatment (eCO_2_) groups, respectively.

#### Modelling

Linear mixed effects models were fitted using the ‘MCMCglmm’ function (MCMCglmm package)^125^ in the R language and environment^126^. This approach implements Markov chain Monte Carlo (MCMC) routines for fitting generalized linear mixed models, while accounting for non-independence and correlated random effects arising from phylogenetic relationships. To do this, the dataset was linked to a completely sampled and dated Bayesian phylogeny that incorporated 11,297 grass taxa^127^ pruned to only include species in this study.

For each of the five grass metrics, a linear mixed effects model was fitted with RR as the response variable and ‘water stress’ (either none or drought), as the main fixed effect of interest. To account for the effect of differences in experimental methodology that may influence RR, additional fixed effects were fitted to account for ‘CO_2_ treatment size’ (the difference in [CO_2_] between aCO_2_ and eCO_2_ treatments), ‘pot size’ (the volume of soil the plant was grown in) and ‘exposure duration’ (to CO_2_ treatments). Exposure methodology was not included as an additional fixed effect because it tended to be correlated with exposure duration and pot size. For example, in the aboveground biomass data, all plants in FACE experiments were grown in the ground with a long exposure to eCO_2_ (mean duration = 301 days), whereas plants grown in indoor growth chambers were exposed for shorter durations (mean duration = 89 days) and were grown in pots (45% in pots less than 10 l). ‘Species’ and ‘study’ (an identifier for the study) were included as random effects. Observation-level sampling variance was included as mev = s.e.^2^, because MCMCglmm defines mev as the known measurement-error variance for each effect size. This separates within-study sampling error from between-study heterogeneity, so more precise estimates are weighted more strongly, whereas the random effects capture residual among-study variation. Models were run for 1,000,000 iterations with a burn-in of 1,000 iterations, and parameter-expanded priors for the random effects (*V* = diag(1), nu = 1, alpha.mu = 0 and alpha.*V* = diag(1) × 1,000). Convergence of models was tested using the Heidelberger convergence diagnostic test (‘heidel.diag’ function in the Coda package^128^). The Emmeans package^129^ was then used to produce estimated marginal mean response ratios and their 95% confidence intervals from MCMCglmm model outputs.

To ensure our approach of accounting for CO_2_ treatment size effects on trait responses described above (that is, including CO_2_ treatment size as a fixed effect in models) was robust, an alternative, complementary approach was taken. A different metric (‘relativized β-factor’), proposed by Walker et al. (equation (1) in ref. ^130^), that standardizes CO_2_ effects on traits by the change in CO_2_ concentration was used as an alternative to RRs. For this metric, β = 1 represents direct proportionality between a variable’s CO_2_ response and the change in CO_2_. MCMCglmm models were run for all five plant traits as described above, except the response variable was the relativized β-factor (rather than RR) and CO_2_ treatment size was removed as an explanatory variable. The results were similar to those gained from the RR approach (Supplementary Fig. 6), and so we only describe the RR model outputs in the main text for ease of comparison with the field and modelling components of this paper.

#### Publication bias

Publication bias was assessed visually through funnel plots of the inverse variance and effect size residuals (Supplementary Fig. 7). There was little potential for bias across the whole dataset, and both positive and negative RRs have been well represented in the data.

#### CO_2_ effects on plant water relations

To further explore CO_2_ effects on plant water relations under different water availabilities, values of leaf water potential and soil water content were extracted from eight studies identified in the meta-analysis^43,57,65,69,75,80,95,97^ (study details given in Supplementary Table 6) that measured these traits under different CO_2_ and water-availability treatments. The low-water-availability treatments of these studies involved reductions in soil water contents ranging from 23% to 83%, or (when soil water content information was not given) a reduction in water supply of 33–99%, relative to the high-water-availability treatment (see Supplementary Table 6 for details). This dataset contained 41 paired leaf water potential measurements (*n* = 82 total; 38 control and 44 drought) and 90 paired soil water content measurements (*n* = 180; 88 control and 92 drought) at aCO_2_ and eCO_2_ for 11 C_4_ grass species. MCMCglmm models were fitted to these data with CO_2_ and water treatment (and their interaction if significant) as explanatory variables. All other model specifications were the same as above except ‘exposure duration’ (to CO_2_ treatment) was not included as an explanatory variable due to a bias in the proportion of observations (that is, 62% and 78% of soil water content and leaf water potential measurements) arising from experiments with a long exposure (more than 120 days) to CO_2_ treatments in this subset of studies. No observation-level sampling variance was included in the soil water content model as the error on these measurements was rarely provided. The extracted model coefficients are plotted in Fig. 1b.

For insights into underlying physiological mechanisms, we explored how interrelated plant water relations parameters changed in relation to CO_2_ and water using a hydraulic–stomatal modelling approach. Using stomatal and hydraulic parameters derived for a widespread, C_4_ savanna grass, *Themeda triandra*^131^ (Supplementary Table 7), the responses of stomatal conductance (*G*_*s*_) and leaf water potential (*Ψ*_leaf_) to declining soil water potential (*Ψ*_soil_) and different CO_2_ treatments (aCO_2_ versus eCO_2_) were modelled. Three equations were used to simulate these responses (following ref. ^132^). Equation (1) determines *Ψ*_leaf_ from plant hydraulic conductance (*K*_plant_) and *G*_*s*_ at a given *Ψ*_soil_ and vapour pressure deficit (VPD) based on steady-state water transport according to the Ohm’s law analogy^133^,

1 $${\varPsi }_{\mathrm{leaf}}={\varPsi }_{\mathrm{soil}}-\frac{{G}_{s}\,\mathrm{VPD}}{{K}_{\mathrm{plant}}}.$$

Equation (2) models the vulnerability of *K*_plant_ to declining *Ψ*_leaf_ as the sigmoidal function given by

2 $${K}_{\mathrm{plant}}=\frac{{K}_{\max }}{1+{e}^{-({\varPsi }_{\mathrm{leaf}}-{P}_{K50})/c}}$$

where *K*_max_ is the maximum leaf hydraulic conductivity under well-watered conditions, and *P*_*K*50_ is *Ψ*_leaf_ at 50% loss of hydraulic conductivity in leaves. The constant *c* defines the shape of the sigmoidal curve. Equation (3) models the decline in *G*_*s*_ with more negative *Ψ*_leaf_ as the sigmoidal function

3 $${G}_{s}=\frac{{G}_{{s}_{\mathrm{max}}}}{1+{e}^{-({\varPsi }_{{\rm{l}}{\rm{e}}{\rm{a}}{\rm{f}}}-{P}_{Gs50)}/c}}$$

where ${G}_{{s}_{\mathrm{max}}}$ is the maximum light-saturated stomatal conductance under well-watered conditions, *P*_*Gs*50_ is the *Ψ*_leaf_ at 50% stomatal closure, and *c* is a constant that defines the shape of the sigmoidal curve.

The responses of *G*_*s*_ and *Ψ*_leaf_ were simulated for a range of *Ψ*_soil_ (from 0 to −5 MPa), at a VPD of 2.5 kPa (chosen to represent a high VPD scenario typical of savanna ecosystems), and under both aCO_2_ and eCO_2_ by simultaneously solving equations (1)–(3) in base R. The modelled relationships are displayed in Fig. 1c, along with estimated marginal means of leaf water potential and stomatal conductance under different CO_2_ and water-availability treatments (derived from studies listed in Supplementary Table 6).

### Field observations

#### Study area

Kruger covers nearly 20,000 km^2^ in low-elevation areas (260–839 m; the ‘lowveld’) of northeastern South Africa, spanning tropical and subtropical latitudes (22° 20′ to 25° 30′ S; 31° 10′ to 32° 00′ E; Supplementary Fig. 1). It is dominated by two underlying parent materials, a granite and a basalt, producing soils that are broadly characterized as sandy and nutrient poor versus clay and nutrient rich, respectively^13^. Mean annual rainfall ranges from 350 mm in the north to 750 mm in the south, although inter-annual variation is significant. Park management continuously maintains 21 daily rainfall measurement stations throughout the park and one daily temperature measurement station is maintained by South African Weather Services. The flora of Kruger includes 400 or more species of tree and shrub and 200 or more species of grass.

Fire is a major ecological feature of the park; the average fire return interval is about 3.5 years, but fire regimes vary locally. Fire frequencies range from one fire per year to one every 34 years. The park is also host to a diverse assemblage of African mammals. Elephants (*Loxodonta africana*) occur at a density of 0.7 km^−2^ and make up the largest propotion of herbivore biomass (1,900 kg km^−2^). Herbivore biomass totals approximately 6,080 kg km^−2^ (10 large stock units (LSU) km^−2^). Because spatially resolved herbivore density estimates are not available, we have not included this explicitly in this analysis, but note that herbivore population sizes have increased significantly in the past several decades (Supplementary Fig. 2) and therefore cannot be responsible for any increases in grass biomass (but see ref. ^134^ for short-term grass–grazer interactions). However, rhino poaching has locally resulted in shifts from grazing lawn to bunchgrass, which we examine via changes in grass species composition (see below).

#### Grass biomass data

Kruger management established 533 veld condition assessment (VCA) sites (343 on granitic and 190 on basaltic soils) to monitor grass biomass to inform fire management, starting in 1989 (Supplementary Fig. 1). Sites were thus located primarily in areas where herbivore impacts are relatively mild (that is, sites were not located in areas dominated by grazing lawns, such as sodic sites and near rivers, where fires are rare). Grass biomass was measured every year in February or March, which corresponds to the end of the growing season and therefore peak standing grass biomass, in plots of 50 m × 60 m with a calibrated disc pasture meter (DPM). Measurements to derive plot-level grass biomass estimates were taken every 2 m along four 50-m transects, running at 0 m, 20 m, 40 m and 60 m along the length of the plot, for a total of 104 DPM estimates per plot. A local DPM calibration is available from Trollope and Potgieter (1986)^135^ (Supplementary Fig. 8), showing reasonable accuracy at the rate of DPM sampling performed here. The most recent grass data available for this analysis were collected in 2021. The subset of plots that were actually sampled for grass biomass and community composition each year varied substantially in number and identity; plot sampling intensity has decreased over time to approximately 150 per year in 2020, reflecting a decrease in investment in monitoring by park managers.

Grass species were identified and recorded. From 1989 through 2006, grass identifications were restricted to 21 indicator species, termed ‘key’ species, with additional categories for ‘other grasses’, ‘forbs’ and ‘bare ground’. Species composition was not recorded in 2007 or 2010–2012. In 2008–2009, grass identification reverted to the original methodology, but with the addition of 9 key species (for a total of 30). Starting in 2016, all grass species were identified, and a category for ‘sedges’ was added. See ref. ^136^ for comprehensive descriptions of ‘key species’ and species-composition methodology. Here, to aid comparison across years, we have degraded more recent community composition sampling to the taxonomic resolution of the original 1989–2006 dataset, focusing our analysis on changes through time in the relative abundances of the five most common key species that were recorded throughout the sampling period: *Bothriochloa radicans*, *Digitaria eriantha*, *Megathyrsus maximus*, *Themeda triandra* and *Urochloa mosambicensis*.

Across the VCA plots, 94 C_4_ grass species have been identified, representing 43 out of the 314 genera that contain C_4_ species, and 3 out of the 4 grass subfamilies where C_4_ photosynthesis has evolved (Aristoideae, Chloridoideae, Panicoideae but not Micrairoideae, which is concentrated in Australia and Asia). All three C_4_ subtypes are present in approximately equal proportions (in 2021, 32%, 39% and 29% of individual species observations were NAD-ME, NADP-ME and PCK subtypes, respectively). Species are broadly representative of those found in arid-to-mesic tropical and subtropical savannas but not high-rainfall savannas (Supplementary Fig. 5b).

#### Rainfall and fire occurrence

Since 1988, park management has maintained 21 daily rainfall measurement stations throughout the park, which we used to generate rainfall surfaces via inverse distance weighting for extraction and comparison with grass biomass and fire occurrences at monitoring sites. Of these stations, three have been continuously maintained since 1960, which were used to estimate rainfall for comparison with parkwide burned area. Maximum daily temperature has been measured at one station across both time periods. Averaging across the entire park, there has been no systematic change through time in rainfall, number of days with rainfall or rainfall per rainfall day, but mean annual daily maximum temperature increased between 1989 and 2021 (Extended Data Fig. 4).

Kruger management also maintains records of the spatial distribution of fires throughout the park, whether accidental or set by management; older records were kept by hand and have been digitized, whereas more recent records were based on fire-scar mapping from MODIS satellite-derived data (accurate to 250 m).

#### Grass functional trait data

This dataset includes maximum culm height (hereafter, ‘height’), specific leaf area (SLA), leaf carbon-to-nitrogen ratio, leaf nitrogen per area, leaf nitrogen per mass and stomatal conductance (*G*_*s*_). Species-level data on height were extracted from ref. ^137^, and for the other traits from the TRY database^33,138–166^. For each trait, we calculated a community mean trait value weighted by relative abundance (community weighted mean) for all plots with more than 80% of species relative abundance represented. Trait coverage was sufficient to calculate community weighted means for more than 50% of plots for only height and SLA, so we restricted our analysis to those traits.

#### Data analysis

Except where noted, statistical analyses were performed on all data except from the year 2000, when record rainfall resulted in widespread flooding (mostly from surface runoff) and thus a vegetation response that is uncoupled from total rainfall amount. Data were analysed via linear mixed effects models using the R ‘lmer’ function in ‘lme4’^167^ with plot as a random effect, and effect sizes were estimated in the ‘emmeans’ package^129^.

Model selection was done via minimization of Akaike Information Criterion corrected for small sample sizes (AICc); we selected as ‘best’ the simplest model within two AICc units of the model with the lowest overall AICc. We tested four different models for grass biomass: (1) grass biomass dependence on soil type, annual rainfall, previous year annual rainfall, rainfall events (in number of days), annual mean daily temperature, time since fire, and year, on a data subset including all grass observations; (2) the same set of factors as (1) but on a data subset excluding sites with more than 20% relative abundance of three common lawn grass species (*Urochloa mosambicensis*, *Digitaria eriantha* and *Megathyrsus coloratum*) to exclude the possibility that local loss of large grazers such as rhinos could be driving temporal change; (3) the same set of factors as (1) and also including relative abundances of the five most common species across our dataset (*Urochloa mosambicensis*, *Bothriochloa radicans*, *Digitaria eriantha*, *Themeda triandra* and *Megathyrsus maximus*) to disentangle the role of community turnover in producing changes in grass biomass; and finally (4) the same set of factors as (1) and also including community weighted means of specific leaf area and individual height to further disentangle the role of trait turnover in creating changes in grassy biomass production. We considered only additive combinations of factors but tested the significance of interactions between year and rainfall and between year and soil type, to evaluate whether possible increases in grass biomass have varied systematically depending on environmental context. In addition, we performed a sensitivity analysis examining the potential effects of DPM-associated errors in estimated grass biomass by randomly sampling observation errors from the DPM calibration and repeating this process 100 times; all runs yielded the same best statistical model as the non-randomized analysis, showing that statistical results were robust to DPM sampling errors (Supplementary Fig. 8). We also evaluated possible model sensitivity to decreases in sampling intensity through time by randomly sampling the full dataset to standardize effort through time; this process also yielded the same best statistical model as the statistical model on the full dataset, with similar effect sizes.

We examined drivers of variation in fire activity in two ways. First, we modelled fire occurrences (yes or no) at each of the VCA sites over time, which we modelled via binomial mixed effects models with ‘glmer’ (lme4 (ref. ^167^)), using site as a random effect, in two ways: (1) fire occurrence depending on rainfall, previous year rainfall, rainfall events, temperature and year; and (2) fire occurrence depending on grass biomass and year. Second, we modelled parkwide proportional burned area through time using a linear model with respect to variation in annual rainfall, previous year rainfall, rainfall events, temperature and year.

#### Comparison of magnitude of CO_2_ effect with published estimates

Estimates of CO_2_-driven increases in aboveground grass productivity have been compared with published estimates from refs. ^1,29^.

In ref. ^29^, grasses were subjected to a gradient of CO_2_ concentrations from 250 to 500 ppm, allowing us to estimate the difference in productivity between [CO_2_] of 350 ppm (equivalent to 1989 levels) and 415 ppm (equivalent to 2021 levels). We digitized the data from figure 2a–c in ref. ^29^, and fitted new linear models to estimate increases for all three soil types included in that work. Confidence intervals were estimated from modelled increases spanning all three soil types.

In ref. ^1^, we used only estimates that were directly based on satellite-informed estimates of gross primary production (GPP), avoiding estimates that made strong theoretical assumptions about physiological optimality, which generally have been developed for C_3_ plants. To do this, we digitized supplementary figure 5b, subpanel ‘C_4_ biomes’, focusing on satellite-derived estimates corresponding with bar ‘C_1_’. Confidence intervals were estimated by digitizing estimates from all remote sensing products. To convert GPP carbon into biomass ANPP, we assumed an average carbon-use efficiency of 0.48 for savannas in the conversion of photosynthate to biomass^168^ and a carbon concentration for grass of 0.42 (from ref. ^169^).

### Land surface and vegetation modelling

To simulate land surface processes in African savannas, we used the CLM5 (ref. ^6^), which is the terrestrial component of the Community Earth System Model version 2 (CESM2; ref. ^170^). We used the standard download of the CLM5 model, available publicly on GitHub (https://github.com/escomp/ctsm), and ran the configuration of this model recommended by its developers. CLM5 represents terrestrial carbon and nitrogen biogeochemistry, vertically resolved soil carbon, and nitrification–denitrification to treat the response of vegetation to future climate and fires. The CLM5 land units are vegetated, lake, urban, glacier and crop. Vegetation and crops are represented by plant functional types (PFTs), each with its own set of ecophysiological, morphological, phenological and biogeochemical parameters. The default PFT distribution of natural vegetation and crops was derived from satellite observations (for example, MODIS) and agricultural census data^171,172^. There are 16 types of natural vegetation (including C_4_ grasses) and 8 active crops^6^.

For C_4_ grasses, photosynthesis follows the Collatz et al. (1992) model^30^, which calculates net photosynthesis based on Rubisco and phosphoenolpyruvate (PEP) carboxylase-limited rates of carboxylation, assuming a sharply saturating response to intercellular CO_2_ concentrations and including limitations from light availability and temperature^6^. The temperature optimum for C_4_ productivity, set via the maximum carboxylation rate of Rubisco (*V*_C__max_), is 36.3 °C (ref. ^173^) (Supplementary Fig. 21). Stomatal conductance for CO_2_ and water is calculated using the Medlyn et al. (2011) model^32^, which assumes a linear increase with net leaf photosynthesis and non-linear declines with VPD and aCO_2_ concentration. This framework is applied to both C_3_ and C_4_ plants. For C_4_ grasses, CLM5 uses empirical parameter values for the Medlyn slope parameter (g1) derived from the global synthesis of Lin et al.^33^, following implementation tests in CABLE by de Kauwe et al.^174^. Both photosynthesis and stomatal conductance are calculated for sunlit and shaded leaves to capture their distinct microenvironments.

Carbon uptake by C_4_ grasses is driven by prescribed photosynthetic parameters (for example, *V*_pmax_ (the maximum catalytic rate of PEP carboxylase, *V*_Cmax_ and *J*_max_ (the maximum rate of photosynthetic electron transport)). These parameters do not adjust dynamically in response to CO_2_ or nitrogen^6^. However, carbon–nitrogen interactions are represented through fixed carbon-to-nitrogen ratios for plant tissues, and nitrogen uptake is regulated by the fixation and uptake of nitrogen (FUN) model^175^, which assigns carbon costs to nitrogen acquisition from soil and internal remobilization. Nitrogen limitation can constrain tissue growth (for example, leaf area) and thereby indirectly influence canopy-scale uptake under eCO_2_.

Carbon allocation in CLM5 follows a hierarchical priority scheme, in which carbon is first used for maintenance respiration, then stored for future needs, and finally allocated to new growth. For grasses, including C_4_ grasses, carbon allocation is primarily determined by fixed allometric parameters that set the proportion of carbon allocated to leaves, stems and roots. Specifically, fine root allocation is set as a fixed ratio relative to leaf biomass, whereas coarse root allocation follows a predefined proportion relative to stem biomass. Although these allocation ratios remain fixed, environmental factors such as CO_2_ concentration, temperature, soil moisture and nutrient availability can influence photosynthesis and overall carbon uptake, indirectly affecting carbon accumulation.

The water–carbon coupling in CLM5 is managed by a plant hydraulic stress approach^176^, which models water transport through plant tissues and calculates water potentials for roots, xylem and leaves. This balances water supply and demand by modulating maximum stomatal conductance to prevent high xylem tension and low leaf water potential. Soil water limitation effects on photosynthesis are quantified using a diagnostic soil moisture stress factor, which assesses the influence of plant water status on carbon cycling processes. A more detailed explanation with specific formulations and parameterizations is provided in refs. ^6,31^.

CLM5 also includes a prognostic treatment of fires based on a modified version of the fire algorithm from refs. ^177,178^. The fire algorithm accounts for agricultural, deforestation, peat and landscape fires, and estimates area burned and fire emissions using information about climate and weather conditions, vegetation composition and structure, and human activity. After the burned area is determined, the fire module calculates the impact of fire, including biomass and peat carbon losses, fire-induced vegetation mortality, adjustment of the vegetation carbon-to-nitrogen pools, and total fire carbon and other trace gas emissions.

#### Historical simulations

To examine the C_4_ grass sensitivity in the model to climate conditions, we performed single-point simulations at Kruger. We selected two sites with different rainfall conditions: a drier, semi-arid site in the north (22.83° S, 31.25° E; average 420 mm rainfall per year) and a wetter, more mesic site at the southern end of the park (25.12° S, 31.88° E; average 620 mm rainfall per year). At both sites, we converted all PFTs within the 50 × 50 km CLM5 grid cell to C_4_ grass, to focus specifically on C_4_ grass responses and avoid effects from other PFTs (for example, via competition and succession processes), and spun up the model for about 600 years so that all the state variables in the model, including total ecosystem soil carbon, reached equilibrium. The present-day spin-up was based on a historical simulation for 1850–2014 using historical nitrogen and aerosol deposition as well as aCO_2_ forcing^6^. The meteorological forcings were from the Global Soil Wetness Project (GSWP3 version 1; http://hydro.iis.u-tokyo.ac.jp/GSWP3/, last access: 15 November 2023), with forcing data available from 1901 to 2014 and cycled from 1901 to 1920 for years before 1901.

#### Future simulations

For our future sensitivity modelling experiment, and following the historical simulations, the Kruger simulations were run from 2015 to 2100 with changes in CO_2_ concentration and climate following the SSP1-2.6 and SSP3-7.0, representing low and high future warming scenarios, respectively. Following standard practice for offline CLM5 simulations, we applied an anomaly forcing approach^6,179^. Atmospheric forcing variables (for example, temperature, precipitation, wind, pressure, humidity and radiation) from the GSWP3 observations (2001–2014) were used as a baseline, onto which monthly mean anomalies from CESM2 fully coupled simulations performed for the Coupled Model Intercomparison Project phase 6 experiments were superimposed. This approach preserved observed high-frequency variability while imposing long-term climate trends consistent with the Coupled Model Intercomparison Project phase 6 SSP projections.

For both scenarios, simulations were performed by changing only CO_2_ and holding all other forcings at 2015 levels (CO_2_ only); only changing climate and holding all other forcings at 2015 levels (climate only); and changing all forcings (CO_2_ + climate; see Extended Data Fig. 7a for driving variables). For each run, we extracted simulated photosynthesis, aboveground and belowground biomass and total biomass, stomatal conductance, leaf water potential, soil moisture, leaf temperature and total fire carbon emissions from fires at each field site (Extended Data Figs. 8 and 9), which represent specific C_4_ grass PFT conditions.

#### Validation

CLM5 simulations have been extensively evaluated against satellite and tower flux observations, on a global scale, across Africa and including grasslands^180,181^. For this study, we evaluated aboveground biomass model predictions against field observations collected at Kruger for 1989–2014 at sites within each modelled grid square for local validation. We plotted all individual site observations as well as annual means against model predictions for the time period 1989–2014 (Extended Data Fig. 6). This was done for simulations both where CO_2_ concentrations increased during this time period and where they were held at 1989 levels (Extended Data Fig. 6b,c).

### Reporting summary

Further information on research design is available in the Nature Portfolio Reporting Summary linked to this article.

## Online content

Any methods, additional references, Nature Portfolio reporting summaries, source data, extended data, supplementary information, acknowledgements, peer review information; details of author contributions and competing interests; and statements of data and code availability are available at https://doi.org/10.1038/s41586-026-10935-4.

## Supplementary information

Supplementary Information This file contains Supplementary Figs. 1–8, Supplementary Tables 1–7 and Supplementary Discussion.

Reporting Summary

## Data Availability

The data supporting the findings of this study are available from the Dryad Digital Repository (https://doi.org/10.5061/dryad.rbnzs7hq7). The meta-analysis was informed by literature searches conducted using the following databases and repositories: Web of Science (https://clarivate.com/academia-government/scientific-and-academic-research/research-discovery-and-referencing/web-of-science/), Scopus (https://www.elsevier.com/en-gb/products/scopus/search), the White Rose Research Online repository (https://eprints.whiterose.ac.uk/) and the South African National ETD Portal for theses and dissertations (https://spu-za.libguides.com/az/national-etd-portal-south-african-theses-and-dissertations). Trait values for common grass species found in the veld condition assessment plots at Kruger were extracted from the TRY database (https://www.try-db.org/TryWeb/Home.php). Citations for the TRY database and the original publications have been provided.

## Code availability

CLM5.0 is publicly available through the Community Terrestrial System Model (CTSM) GitHub repository^6^.

## References

[1] Chen, C., Riley, W. J., Prentice, I. C. & Keenan, T. F. CO_2_ fertilization of terrestrial photosynthesis inferred from site to global scales. *Proc. Natl Acad. Sci. USA* **119**, e2115627119 (2022). 10.1073/pnas.2115627119 PMC8915860 35238668

[2] Forzieri, G., Dakos, V., McDowell, N. G., Ramdane, A. & Cescatti, A. Emerging signals of declining forest resilience under climate change. *Nature* **608**, 534–539 (2022). 10.1038/s41586-022-04959-9 PMC9385496 35831499

[3] Jia, G. et al. in *Climate Change and Land: an IPCC Special Report on Climate Change, Desertification, Land Degradation, Sustainable Land Management, Food Security, and Greenhouse Gas Fluxes in Terrestrial Ecosystems* (eds Shukla, P. R. et al.) 131–242 (IPCC, 2019).

[4] Hubau, W. et al. Asynchronous carbon sink saturation in African and Amazonian tropical forests. *Nature* **579**, 80–87 (2020). 10.1038/s41586-020-2035-0 PMC7617213 32132693

[5] Grace, J., José, J. S., Meir, P., Miranda, H. S. & Montes, R. A. Productivity and carbon fluxes of tropical savannas. *J. Biogeogr.* **33**, 387–400 (2006).

[6] Lawrence, D. M. et al. The Community Land Model version 5: description of new features, benchmarking, and impact of forcing uncertainty. *J. Adv. Model. Earth Syst.* **11**, 4245–4287 (2019).

[7] Leakey, A. D., Bishop, K. A. & Ainsworth, E. A. A multi-biome gap in understanding of crop and ecosystem responses to elevated CO_2_. *Curr. Opin. Plant Biol.* **15**, 228–236 (2012). 10.1016/j.pbi.2012.01.009 22284851

[8] Pan, Y. et al. Contrasting responses of woody and grassland ecosystems to increased CO_2_ as water supply varies. *Nat. Ecol. Evol.* **6**, 315–323 (2022). 10.1038/s41559-021-01642-6 35027723

[9] Nowak, R. S., Ellsworth, D. S. & Smith, S. D. Functional responses of plants to elevated atmospheric CO_2_—do photosynthetic and productivity data from FACE experiments support early predictions? *New Phytol.* **162**, 253–280 (2004).

[10] Ainsworth, E. A. & Long, S. P. 30 Years of free-air carbon dioxide enrichment (FACE): what have we learned about future crop productivity and its potential for adaptation? *Glob. Chang. Biol.* **27**, 27–49 (2021). 10.1111/gcb.15375 33135850

[11] Bond, W. J. & Midgley, G. F. Carbon dioxide and the uneasy interactions of trees and savannah grasses. *Phil. Trans. R. Soc. B* **367**, 601–612 (2012). 10.1098/rstb.2011.0182 PMC3248705 22232770

[12] Buitenwerf, R., Bond, W. J., Stevens, N. & Trollope, W. S. W. Increased tree densities in South African savannas: >50 years of data suggests CO_2_ as a driver. *Glob. Chang. Biol.* **18**, 675–684 (2012).

[13] Staver, A. C., Botha, J. & Hedin, L. Soils and fire jointly determine vegetation structure in an African savanna. *New Phytol.* **216**, 1151–1160 (2017). 10.1111/nph.14738 28840610

[14] Alvarado, S. T., Andela, N., Silva, T. S. F. & Archibald, S. Thresholds of fire response to moisture and fuel load differ between tropical savannas and grasslands across continents. *Global Ecol. Biogeogr.* **29**, 331–344 (2020).

[15] Leakey, A. D. et al. Photosynthesis, productivity, and yield of maize are not affected by open-air elevation of CO_2_ concentration in the absence of drought. *Plant Physiol.* **140**, 779–790 (2006). 10.1104/pp.105.073957 PMC1361343 16407441

[16] Wand, S. J. E., Midgley, G. F., Jones, M. H. & Curtis, P. S. Responses of wild C_4_ and C_3_ grass (Poaceae) species to elevated atmospheric CO_2_ concentration: a meta-analytic test of current theories and perceptions. *Glob. Chang. Biol.* **5**, 723–741 (1999).

[17] Morgan, J. A. et al. C_4_ grasses prosper as carbon dioxide eliminates desiccation in warmed semi-arid grassland. *Nature* **476**, 202–205 (2011). 10.1038/nature10274 21814202

[18] Reich, P. B., Hobbie, S. E., Lee, T. D. & Pastore, M. A. Unexpected reversal of C_3_ versus C_4_ grass response to elevated CO_2_ during a 20-year field experiment. *Science* **360**, 317–320 (2018). 10.1126/science.aas9313 29674593

[19] Spinoni, J., Naumann, G., Carrao, H., Barbosa, P. & Vogt, J. World drought frequency, duration, and severity for 1951–2010. *Int. J. Climatol.* **34**, 2792–2804 (2014).

[20] Chiang, F., Mazdiyasni, O. & AghaKouchak, A. Evidence of anthropogenic impacts on global drought frequency, duration, and intensity. *Nat. Commun.* **12**, 2754 (2021). 10.1038/s41467-021-22314-w PMC8115225 33980822

[21] Sala, O. E., Gherardi, L. A., Reichmann, L., Jobbágy, E. & Peters, D. Legacies of precipitation fluctuations on primary production: theory and data synthesis. *Phil. Trans. R. Soc. B* **367**, 3135–3144 (2012). 10.1098/rstb.2011.0347 PMC3479688 23045711

[22] Staver, A. C., Abraham, J. O., Hempson, G. P., Karp, A. T. & Faith, J. T. The past, present, and future of herbivore impacts on savanna vegetation. *J. Ecol.* **109**, 2804–2822 (2021).

[23] Smit, I. P., Peel, M. J., Ferreira, S. M., Greaver, C. & Pienaar, D. J. Megaherbivore response to droughts under different management regimes: lessons from a large African savanna. *Afr. J. Range Forage Sci.* **37**, 65–80 (2020).

[24] Stevens, C. J. et al. Anthropogenic nitrogen deposition predicts local grassland primary production worldwide. *Ecology* **96**, 1459–1465 (2015).

[25] Del Toro, I., Case, M. F., Karp, J., Slingsby, J. & Staver, A. C. Carbon isotope trends across a century of herbarium specimens suggest CO_2_ fertilization of C_4_ grasses. *New Phytol.* **243**, 560–566 (2024). 10.1111/nph.19868 38812264

[26] Keeling, R. F., Morgan, E. J. & Keeling, C. D. Atmospheric monthly in situ CO_2_ data—Mauna Loa Observatory, Hawaii. In Scripps CO2 Program Data. *UC San Diego Library Digital Collections* 10.6075/J08W3BHW (2017).

[27] Terrer, C. et al. Nitrogen and phosphorus constrain the CO_2_ fertilization of global plant biomass. *Nat. Clim. Change* **9**, 684–689 (2019).

[28] Fay, P. A. et al. Soil-mediated effects of subambient to increased carbon dioxide on grassland productivity. *Nat. Clim. Change* **2**, 742–746 (2012).

[29] Polley, H. W. et al. CO_2_ enrichment and soil type additively regulate grassland productivity. *New Phytol.* **222**, 183–192 (2019). 10.1111/nph.15562 30367488

[30] Collatz, G. J., Ribas-Carbo, M. & Berry, J. A. Coupled photosynthesis-stomatal conductance model for leaves of C_4_ plants. *Funct. Plant Biol.* **19**, 519–538 (1992).

[31] Kennedy, D. et al. Implementing plant hydraulics in the Community Land Model version 5. *J. Adv. Model. Earth Syst.* **11**, 485–513 (2019).

[32] Medlyn, B. E. et al. Reconciling the optimal and empirical approaches to modelling stomatal conductance. *Glob. Chang. Biol.* **17**, 2134–2144 (2011).

[33] Lin, Y. S. et al. Optimal stomatal behaviour around the world. *Nat. Clim. Change* **5**, 459–464 (2015).

[34] Noy-Meir, I. Desert ecosystems: environment and producers. *Annu. Rev. Ecol. Syst.* **4**, 25–52 (1973).

[35] Piñeiro, J. et al. Effects of elevated CO_2_ on fine root biomass are reduced by aridity but enhanced by soil nitrogen: a global assessment. *Sci. Rep.* **7**, 15355 (2017). 10.1038/s41598-017-15728-4 PMC5681551 29127358

[36] Purcell, C. et al. Increasing stomatal conductance in response to rising atmospheric CO_2_. *Ann. Bot.* **121**, 1137–1149 (2018). 10.1093/aob/mcx208 PMC5946907 29394303

[37] Scoffoni, C., Albuquerque, C., Buckley, T. N. & Sack, L. The dynamic multi-functionality of leaf water transport outside the xylem. *New Phytol.* **239**, 2099–2107 (2023). 10.1111/nph.19069 37386735

[38] Márquez, D. A., Wong, S. C., Stuart-Williams, H., Cernusak, L. A. & Farquhar, G. D. Mesophyll airspace unsaturation drives C_4_ plant success under vapor pressure deficit stress. *Proc. Natl Acad. Sci. USA* **121**, e2402233121 (2024). 10.1073/pnas.2402233121 PMC11441488 39284054

[39] Crafts-Brandner, S. J. & Salvucci, M. E. Sensitivity of photosynthesis in a C_4_ plant, maize, to heat stress. *Plant Physiol.* **129**, 1773–1780 (2002). 10.1104/pp.002170 PMC166765 12177490

[40] Boyd, R. A., Gandin, A. & Cousins, A. B. Temperature responses of C_4_ photosynthesis: biochemical analysis of rubisco, phosphoenolpyruvate carboxylase, and carbonic anhydrase in *Setaria viridis*. *Plant Physiol.* **169**, 1850–1861 (2015). 10.1104/pp.15.00586 PMC4634053 26373659

[41] Yin, X., van der Putten, P. E. L., Driever, S. M. & Struik, P. C. Temperature response of bundle-sheath conductance in maize leaves. *J. Exp. Bot.* **67**, 2699–2714 (2016). 10.1093/jxb/erw104 PMC4861018 26969744

[42] Sonawane, B. V., Sharwood, R. E., von Caemmerer, S., Whitney, S. M. & Ghannoum, O. Short-term thermal photosynthetic responses of C_4_ grasses are independent of the biochemical subtype. *J. Exp. Bot.* **68**, 5583–5597 (2017). 10.1093/jxb/erx350 PMC5853683 29045727

[43] Raubenheimer, S. L., Simpson, K., Carkeek, R. & Ripley, B. Could CO_2_-induced changes to C_4_ grass flammability aggravate savanna woody encroachment? *Afr. J. Range Forage Sci.* **39**, 82–95 (2022).

[44] Reyes-Fox, M. et al. Elevated CO_2_ further lengthens growing season under warming conditions. *Nature* **510**, 259–262 (2014). 10.1038/nature13207 24759322

[45] Abreu, R. C. R. et al. The biodiversity cost of carbon sequestration in tropical savanna. *Sci. Adv.* **3**, e1701284 (2017). 10.1126/sciadv.1701284 PMC5576881 28875172

[46] Zhou, Y. et al. Limited increases in savanna carbon stocks over decades of fire suppression. *Nature* **603**, 445–449 (2022). 10.1038/s41586-022-04438-1 35296846

[47] Poulter, B. et al. Contribution of semi-arid ecosystems to interannual variability of the global carbon cycle. *Nature* **509**, 600–603 (2014). 10.1038/nature13376 24847888

[48] Zhou, Y. et al. Soil carbon in tropical savannas mostly derived from grasses. *Nat. Geosci.* **16**, 710–716 (2023).

[49] Robinson, E. A., Ryan, G. D. & Newman, J. A. A meta-analytical review of the effects of elevated CO_2_ on plant–arthropod interactions highlights the importance of interacting environmental and biological variables. *New Phytol.* **194**, 321–336 (2012). 10.1111/j.1469-8137.2012.04074.x 22380757

[50] Luo, X. et al. Mapping the global distribution of C_4_ vegetation using observations and optimality theory. *Nat. Commun.* **15**, 1219 (2024). 10.1038/s41467-024-45606-3 PMC10858286 38336770

[51] Skowno, A. L. et al. Woodland expansion in South African grassy biomes based on satellite observations (1990–2013): general patterns and potential drivers. *Glob. Chang. Biol.* **23**, 2358–2369 (2017). 10.1111/gcb.13529 27732761

[52] Grames, E. M., Stillman, A. N., Tingley, M. W. & Elphick, C. S. An automated approach to identifying search terms for systematic reviews using keyword co-occurrence networks. *Methods Ecol. Evol.* **10**, 1645–1654 (2019).

[53] Rohatgi, A. WebPlotDigitizer. *GitHub* https://github.com/ankitrohatgi/WebPlotDigitizer (2023).

[54] Abdalla, A. L. et al. Nutritive value and enteric methane production of *Brachiaria* spp. under elevated [CO_2_]. *Int. J. Plant Prod.* **14**, 119–126 (2020).

[55] Abdalla Filho, A. L. et al. Fiber fractions, multielemental and isotopic composition of a tropical C_4_ grass grown under elevated atmospheric carbon dioxide. *PeerJ* **7**, e5932 (2019). 10.7717/peerj.5932 PMC6385687 30809426

[56] Abdalla Filho, A. L. et al. CO_2_ fertilization does not affect biomass production and nutritive value of a C_4_ tropical grass in short timeframe. *Grass Forage Sci.* **74**, 670–677 (2019).

[57] Adam, N. R., Owensby, C. E. & Ham, J. M. The effect of CO_2_ enrichment on leaf photosynthetic rates and instantaneous water use efficiency of *Andropogon gerardii* in the tallgrass prairie. *Photosynth. Res.* **65**, 121–129 (2000). 10.1023/A:1006489919192 16228479

[58] Anderson, L. J., Maherali, H., Johnson, H. B., Polley, H. W. & Jackson, R. B. Gas exchange and photosynthetic acclimation over subambient to elevated CO_2_ in a C_3_–C_4_ grassland. *Glob. Chang. Biol.* **7**, 693–707 (2001).

[59] Barbehenn, R. V., Chen, Z., Karowe, D. N. & Spickard, A. C_3_ grasses have higher nutritional quality than C_4_ grasses under ambient and elevated atmospheric CO_2_. *Glob. Chang. Biol.* **10**, 1565–1575 (2004).

[60] Baruch, Z. & Jackson, R. B. Responses of tropical native and invader C_4_ grasses to water stress, clipping and increased atmospheric CO_2_ concentration. *Oecologia* **145**, 522–532 (2005). 10.1007/s00442-005-0153-x 16003505

[61] Bellasio, C., Quirk, J. & Beerling, D. J. Stomatal and non-stomatal limitations in savanna trees and C_4_ grasses grown at low, ambient and high atmospheric CO_2_. *Plant Sci.* **274**, 181–192 (2018). 10.1016/j.plantsci.2018.05.028 30080602

[62] von Caemmerer, S., Ghannoum, O., Conroy, J. P., Clark, H. & Newton, P. C. D. Photosynthetic responses of temperate species to free air CO_2_ enrichment (FACE) in a grazed New Zealand pasture. *Funct. Plant Biol.* **28**, 439–450 (2001).

[63] Carter, D. R. & Peterson, K. M. Effects of a CO_2_-enriched atmosphere on the growth and competitive interaction of a C_3_ and a C_4_ grass. *Oecologia* **58**, 188–193 (1983). 10.1007/BF00399215 28310576

[64] Carvalho, J. M. et al. Elevated CO_2_ and warming change the nutrient status and use efficiency of *Panicum maximum* Jacq. *PLoS ONE* **15**, e0223937 (2020). 10.1371/journal.pone.0223937 PMC7069640 32168346

[65] Clark, H., Newton, P. C. D. & Barker, D. J. Physiological and morphological responses to elevated CO_2_ and a soil moisture deficit of temperate pasture species growing in an established plant community. *J. Exp. Bot.* **50**, 233–242 (1999).

[66] de Oliveira, A. C. G., Rios, P. M., Pereira, E. G. & Souza, J. P. Growth and competition between a native leguminous forb and an alien grass from the Cerrado under elevated CO_2_. *Austral Ecol.* **46**, 750–761 (2021).

[67] Dijkstra, F. A., Blumenthal, D., Morgan, J. A., LeCain, D. R. & Follett, R. F. Elevated CO_2_ effects on semi-arid grassland plants in relation to water availability and competition. *Funct. Ecol.* **24**, 1152–1161 (2010).

[68] de Faria, A. P., Marabesi, M. A., Gaspar, M. & França, M. G. C. The increase of current atmospheric CO_2_ and temperature can benefit leaf gas exchanges, carbohydrate content and growth in C_4_ grass invaders of the Cerrado biome. *Plant Physiol. Biochem.* **127**, 608–616 (2018). 10.1016/j.plaphy.2018.04.042 29738989

[69] Fravolini, A., Williams, D. G. & Thompson, T. L. Carbon isotope discrimination and bundle sheath leakiness in three C_4_ subtypes grown under variable nitrogen, water and atmospheric CO_2_ supply. *J. Exp. Bot.* **53**, 2261–2269 (2002). 10.1093/jxb/erf084 12379793

[70] Ghannoum, O., Caemmerer, S. V., Barlow, E. W. R. & Conroy, J. P. The effect of CO_2_ enrichment and irradiance on the growth, morphology and gas exchange of a C_3_ (*Panicum laxum*) and a C_4_ (*Panicum antidotale*) grass. *Funct. Plant Biol.* **24**, 227–237 (1997).

[71] Ghannoum, O., von Caemmerer, S. & Conroy, J. P. Plant water use efficiency of 17 Australian NAD-ME and NADP-ME C_4_ grasses at ambient and elevated CO_2_ partial pressure. *Funct. Plant Biol.* **28**, 1207–1217 (2001).

[72] Gifford, R. M. & Morison, J. I. L. Photosynthesis, water use and growth of a C_4_ grass stand at high CO_2_ concentration. *Photosynth. Res.* **7**, 77–90 (1985). 10.1007/BF00032924 24443015

[73] Hager, H. A., Ryan, G. D., Kovacs, H. M. & Newman, J. A. Effects of elevated CO_2_ on photosynthetic traits of native and invasive C_3_ and C_4_ grasses. *BMC Ecol.* **16**, 28 (2016). 10.1186/s12898-016-0082-z PMC4888642 27246099

[74] Hager, H. A., Ryan, G. D. & Newman, J. A. Effects of elevated CO_2_ on competition between native and invasive grasses. *Oecologia* **192**, 1099–1110 (2020). 10.1007/s00442-020-04636-6 32253494

[75] Hamerlynck, E. P., McAllister, C. A., Knapp, A. K., Ham, J. M. & Owensby, C. E. Photosynthetic gas exchange and water relation responses of three tallgrass prairie species to elevated carbon dioxide and moderate drought. *Int. J. Plant Sci.* **158**, 608–616 (1997).

[76] Hunt, H. W., Elliott, E. T., Detling, J. K., Morgan, J. A. & Chen, D.-X. Responses of a C3 and a C_4_ perennial grass to elevated CO_2_ and temperature under different water regimes. *Glob. Chang. Biol.* **2**, 35–47 (1996).

[77] Johnson, S. N., Lopaticki, G. & Hartley, S. E. Elevated atmospheric CO_2_ triggers compensatory feeding by root herbivores on a C_3_ but not a C_4_ grass. *PLoS ONE* **9**, e90251 (2014). 10.1371/journal.pone.0090251 PMC3961222 24651855

[78] Kellogg, E. A., Farnsworth, E. J., Russo, E. T. & Bazzaz, F. Growth responses of C_4_ grasses of contrasting origin to elevated CO_2_. *Ann. Bot.* **84**, 279–288 (1999).

[79] Kgope, B. S., Bond, W. J. & Midgley, G. F. Growth responses of African savanna trees implicate atmospheric [CO_2_] as a driver of past and current changes in savanna tree cover. *Austral Ecol.* **35**, 451–463 (2010).

[80] Knapp, A. K., Hamerlynck, E. P. & Owensby, C. E. Photosynthetic and water relations responses to elevated CO_2_ in the C_4_ grass *Andropogon gerardii*. *Int. J. Plant Sci.* **154**, 459–466 (1993).

[81] LeCain, D. R. et al. Root biomass of individual species, and root size characteristics after five years of CO_2_ enrichment on native shortgrass steppe. *Plant Soil* **279**, 219–228 (2006).

[82] LeCain, D. R. & Morgan, J. A. Growth, gas exchange, leaf nitrogen and carbohydrate concentrations in NAD-ME and NADP-ME C_4_ grasses grown in elevated CO_2_. *Physiol. Plant.* **102**, 297–306 (1998).

[83] LeCain, D. R., Morgan, J. A., Mosier, A. R. & Nelson, J. A. Soil and plant water relations determine photosynthetic responses of C3 and C4 grasses in a semi-arid ecosystem under elevated CO_2_. *Ann. Bot.* **92**, 41–52 (2003). 10.1093/aob/mcg109 PMC4243634 12754182

[84] Lee, T. D., Barrott, S. H. & Reich, P. B. Photosynthetic responses of 13 grassland species across 11 years of free-air CO_2_ enrichment is modest, consistent and independent of N supply. *Glob. Chang. Biol.* **17**, 2893–2904 (2011).

[85] Lee, T. D., Tjoelker, M. G., Ellsworth, D. S. & Reich, P. B. Leaf gas exchange responses of 13 prairie grassland species to elevated CO_2_ and increased nitrogen supply. *New Phytol.* **150**, 405–418 (2001).

[86] Maherali, H., Reid, C. D., Polley, H. W., Johnson, H. B. & Jackson, R. B. Stomatal acclimation over a subambient to elevated CO_2_ gradient in a C_3_/C_4_ grassland. *Plant Cell Environ.* **25**, 557–566 (2002).

[87] Manea, A., Leishman, M. R. & Downey, P. O. Exotic C_4_ grasses have increased tolerance to glyphosate under elevated carbon dioxide. *Weed Sci.* **59**, 28–36 (2011).

[88] Marks, S. & Clay, K. Effects of CO_2_ enrichment, nutrient addition, and fungal endophyte-infection on the growth of two grasses. *Oecologia* **84**, 207–214 (1990). 10.1007/BF00318273 28312754

[89] McGranahan, D. A. & Yurkonis, K. A. Variability in grass forage quality and quantity in response to elevated CO_2_ and water limitation. *Grass Forage Sci.* **73**, 517–521 (2018).

[90] Morgan, J. A., Knight, W. G., Dudley, L. M. & Hunt, H. W. Enhanced root system C-sink activity, water relations and aspects of nutrient acquisition in mycotrophic *Bouteloua gracilis* subjected to CO_2_ enrichment. *Plant Soil* **165**, 139–146 (1994).

[91] Morgan, J. A., LeCain, D. R., Read, J. J., Hunt, H. W. & Knight, W. G. Photosynthetic pathway and ontogeny affect water relations and the impact of CO_2_ on *Bouteloua gracilis* (C_4_) and *Pascopyrum smithii* (C_3_). *Oecologia* **114**, 483–493 (1998). 10.1007/s004420050472 28307897

[92] Morgan, J. A., Lecain, D. R., Mosier, A. R. & Milchunas, D. G. Elevated CO_2_ enhances water relations and productivity and affects gas exchange in C_3_ and C_4_ grasses of the Colorado shortgrass steppe. *Glob. Chang. Biol.* **7**, 451–466 (2001).

[93] Morgan, J. A. et al. CO_2_ enhances productivity, alters species composition, and reduces digestibility of shortgrass steppe vegetation. *Ecol. Appl.* **14**, 208–219 (2004).

[94] Newman, Y. C., Sollenberger, L. E., Boote, K. J., Allen, L. H. & Littell, R. C. Carbon dioxide and temperature effects on forage dry matter production. *Crop Sci.* **41**, 399–406 (2001).

[95] Pallett, N. *The Effects of Elevated CO*_*2*_* on C*_*4*_* Panicoid Grass Drought Tolerance*. MSc Thesis, Rhodes Univ. (2018).

[96] Pastore, M. A., Lee, T. D., Hobbie, S. E. & Reich, P. B. Strong photosynthetic acclimation and enhanced water-use efficiency in grassland functional groups persist over 21 years of CO_2_ enrichment, independent of nitrogen supply. *Glob. Chang. Biol.* **25**, 3031–3044 (2019). 10.1111/gcb.14714 31148322

[97] Pastore, M. A., Lee, T. D., Hobbie, S. E. & Reich, P. B. Interactive effects of elevated CO_2_, warming, reduced rainfall, and nitrogen on leaf gas exchange in five perennial grassland species. *Plant Cell Environ.* **43**, 1862–1878 (2020). 10.1111/pce.13783 32400900

[98] Paterson, E., Rattray, E. A. S. & Killham, K. Effect of elevated atmospheric CO_2_ concentration on C-partitioning and rhizosphere C-flow for three plant species. *Soil Biol. Biochem.* **28**, 195–201 (1996).

[99] Polley, H. W., Johnson, H. B., Mayeux, H. S. & Brown, D. A. Leaf and plant water use efficiency of C_4_ species grown at glacial to elevated CO_2_ concentrations. *Int. J. Plant Sci.* **157**, 164–170 (1996).

[100] Quirk, J., Bellasio, C., Johnson, D. A., Osborne, C. P. & Beerling, D. J. C_4_ savanna grasses fail to maintain assimilation in drying soil under low CO_2_ compared with C_3_ trees despite lower leaf water demand. *Funct. Ecol.* **33**, 388–398 (2019).

[101] Reich, P. B. et al. Do species and functional groups differ in acquisition and use of C, N and water under varying atmospheric CO_2_ and N availability regimes? A field test with 16 grassland species. *New Phytol.* **150**, 435–448 (2001).

[102] Rudmann, S. G., Milham, P. J. & Conroy, J. P. Influence of high CO_2_ partial pressure on nitrogen use efficiency of the C_4_ Grasses *Panicum coloratum* and *Cenchrus ciliaris*. *Ann. Bot.* **88**, 571–577 (2001).

[103] Runion, G. B., Prior, S. A., Capo-chichi, L. J. A., Torbert, H. A. & van Santen, E. Varied growth response of cogongrass ecotypes to elevated CO_2_. *Front. Plant Sci.* **6**, 1182 (2016). 10.3389/fpls.2015.01182 PMC4700147 26779216

[104] Seneweera, S. P., Ghannoum, O. & Conroy, J. High vapour pressure deficit and low soil water availability enhance shoot growth responses of a C_4_ grass (*Panicum coloratum* cv. Bambatsi) to CO_2_ enrichment. *Funct. Plant Biol.* **25**, 287–292 (1998).

[105] Seneweera, S. P., Ghannoum, O. & Conroy, J. P. Root and shoot factors contribute to the effect of drought on photosynthesis and growth of the C_4_ grass *Panicum coloratum* at elevated CO_2_ partial pressures. *Aust. J. Plant Physiol.* 10.1071/PP01007 (2001).

[106] Sionit, N. & Patterson, D. T. Responses of C_4_ grasses to atmospheric CO_2_ enrichment. *Oecologia* **65**, 30–34 (1984). 10.1007/BF00384459 28312106

[107] Smith, S., Strain, B. & Sharkey, T. Effects of CO_2_ enrichment on four Great Basin grasses. *Funct. Ecol.* **1**, 139–143 (1987).

[108] Tooth, I. M. & Leishman, M. R. Post-fire resprouting responses of native and exotic grasses from Cumberland Plain Woodland (Sydney, Australia) under elevated carbon dioxide. *Austral Ecol.* **38**, 1–10 (2013).

[109] Tooth, I. M. & Leishman, M. R. Elevated carbon dioxide and fire reduce biomass of native grass species when grown in competition with invasive exotic grasses in a savanna experimental system. *Biol. Invasions* **16**, 257–268 (2014).

[110] Volin, J. C., Reich, P. B. & Givnish, T. J. Elevated carbon dioxide ameliorates the effects of ozone on photosynthesis and growth: species respond similarly regardless of photosynthetic pathway or plant functional group. *New Phytol.* **138**, 315–325 (1998). 10.1046/j.1469-8137.1998.00100.x 33863086

[111] Wand, S. J. E. & Midgley, G. F. Effects of atmospheric CO_2_ concentration and defoliation on the growth of *Themeda triandra*. *Grass Forage Sci.* **59**, 215–226 (2004).

[112] Wand, S., Midgley, G. & Stock, W. Response to elevated CO_2_ from a natural spring in a C_4_-dominated grassland depends on seasonal phenology. *Afr. J. Range Forage Sci.* **19**, 81–91 (2002).

[113] Wand, S. J. E., Midgley, G. F. & Musil, C. F. Physiological and growth responses of two African species, *Acacia karroo* and *Themeda triandra*, to combined increases in CO_2_ and UV-B radiation. *Physiol. Plant.* **98**, 882–890 (1996).

[114] Wand, S. J. E., Midgley, G. F. & Stock, W. D. Growth responses to elevated CO_2_ in NADP-ME, NAD-ME and PCK C_4_ grasses and a C_3_ grass from South Africa. *Funct. Plant Biol.* **28**, 13–25 (2001).

[115] Watling, J. R. & Press, M. C. How does the C_4_ grass *Eragrostis pilosa* respond to elevated carbon dioxide and infection with the parasitic angiosperm *Striga hermonthica*? *New Phytol.* **140**, 667–675 (1998). 10.1046/j.1469-8137.1998.00303.x 33862957

[116] Weller, S. L., Florentine, S. K., Mutti, N. K., Jha, P. & Chauhan, B. S. Response of *Chloris truncata* to moisture stress, elevated carbon dioxide and herbicide application. *Sci. Rep.* **9**, 10721 (2019). 10.1038/s41598-019-47237-x PMC6656741 31341230

[117] Wilsey, B. J., Coleman, J. S. & McNaughton, S. J. Effects of elevated CO_2_ and defoliation on grasses: a comparative ecosystem approach. *Ecol. Appl.* **7**, 844–853 (1997).

[118] Wilsey, B. J., McNaughton, S. J. & Coleman, J. S. Will increases in atmospheric CO_2_ affect regrowth following grazing in C_4_ grasses from tropical grasslands? A test with *Sporobolus kentrophyllus*. *Oecologia* **99**, 141–144 (1994). 10.1007/BF00317094 28313959

[119] Xiao, L., Liu, G. & Xue, S. Elevated CO_2_ concentration and drought stress exert opposite effects on plant biomass, nitrogen, and phosphorus allocation in *Bothriochloa ischaemum*. *J. Plant Growth Regul.* **35**, 1088–1097 (2016).

[120] Xu, Z. et al. Effects of elevated CO_2_, warming and precipitation change on plant growth, photosynthesis and peroxidation in dominant species from North China grassland. *Planta* **239**, 421–435 (2014). 10.1007/s00425-013-1987-9 24463932

[121] Yu, J., Sun, L., Fan, N., Yang, Z. & Huang, B. Physiological factors involved in positive effects of elevated carbon dioxide concentration on Bermudagrass tolerance to salinity stress. *Environ. Exp. Bot.* **115**, 20–27 (2015).

[122] Ziska, L. H., Hogan, K. P., Smith, A. P. & Drake, B. G. Growth and photosynthetic response of nine tropical species with long-term exposure to elevated carbon dioxide. *Oecologia* **86**, 383–389 (1991). 10.1007/BF00317605 28312925

[123] Hedges, L. V., Gurevitch, J. & Curtis, P. S. The meta-analysis of response ratios in experimental ecology. *Ecology* **80**, 1150–1156 (1999).

[124] Bracken, M. B. in *Effective Care of Newborn Infants* (eds Sinclair, J. C. & Bracken M. B.) 13–20 (Oxford Univ. Press, 1992).

[125] Hadfield, J. D. MCMC methods for multi-response generalized linear mixed models: the MCMCGLMM R package. *J. Stat. Softw.* **33**, 1–22 (2010).

[126] R Core Team. *R: a Language and Environment for Statistical Computing* https://www.R-project.org/ (R Foundation for Statistical Computing, 2023).

[127] Forrestel, E. J. *Biogeographic Influences on Grassland Community Structure and Function*. PhD Thesis, Yale Univ. (2015).

[128] Plummer, M., Best, N., Cowles, K. & Vines, K. CODA: convergence diagnosis and output analysis for MCMC. *R News* **6**, 7–11 (2006).

[129] Lenth, R. emmeans: Estimated marginal means, aka least-squares means. R package version 1.9.0 https://CRAN.R-project.org/package=emmeans (CRAN, 2023).

[130] Walker, A. P. et al. Integrating the evidence for a terrestrial carbon sink caused by increasing atmospheric CO_2_. *New Phytol.* **229**, 2413–2445 (2021). 10.1111/nph.16866 32789857

[131] Jacob, V. et al. High safety margins to drought-induced hydraulic failure found in five pasture grasses. *Plant Cell Environ.* **45**, 1631–1646 (2022). 10.1111/pce.14318 35319101

[132] Osborne, C. P. & Sack, L. Evolution of C_4_ plants: a new hypothesis for an interaction of CO_2_ and water relations mediated by plant hydraulics. *Phil. Trans. R. Soc. B* **367**, 583–600 (2012). 10.1098/rstb.2011.0261 PMC3248710 22232769

[133] Wei, C., Tyree, M. T. & Steudle, E. Direct measurement of xylem pressure in leaves of intact maize plants. A test of the cohesion-tension theory taking hydraulic architecture into consideration. *Plant Physiol.* **121**, 1191–1206 (1999). 10.1104/pp.121.4.1191 PMC59486 10594106

[134] Staver, A. C., Wigley-Coetsee, C. & Botha, J. Grazer movements exacerbate grass declines during drought in an African savanna. *J. Ecol.* **107**, 1482–1491 (2019).

[135] Trollope, W. S. W. & Potgieter, A. L. F. Estimating grass fuel loads with a disc pasture meter in the Kruger National Park. *J. Grassl. Soc. S. Afr.* **3**, 148–152 (1986).

[136] Wigley-Coetsee, C. & Staver, A. Grass community responses to drought in an African savanna. *Afr. J. Range Forage Sci.* **37**, 43–52 (2020).

[137] Clayton, W. D., Vorontsova, M. S., Harman, K. T. & Williamson, H. GrassBase—the online world. *Kew* http://www.kew.org/data/grasses-db.html (2006).

[138] Kattge, J. et al. TRY plant trait database—enhanced coverage and open access. *Glob. Chang. Biol.* **26**, 119–188 (2020). 10.1111/gcb.14904 31891233

[139] Adler, P. B. et al. Functional traits explain variation in plant life history strategies. *Proc. Natl Acad. Sci. USA* **111**, 740–745 (2014). 10.1073/pnas.1315179111 PMC3896207 24379395

[140] Atkin, O. K. et al. Global variability in leaf respiration in relation to climate, plant functional types and leaf traits. *New Phytol.* **206**, 614–636 (2015). 10.1111/nph.13253 25581061

[141] Baruch, Z. & Goldstein, G. Leaf construction cost, nutrient concentration, and net CO_2_ assimilation of native and invasive species in Hawaii. *Oecologia* **121**, 183–192 (1999). 10.1007/s004420050920 28308558

[142] Catford, J. A., Morris, W. K., Vesk, P. A., Gippel, C. J. & Downes, B. J. Species and environmental characteristics point to flow regulation and drought as drivers of riparian plant invasion. *Diversity Distrib.* **20**, 1084–1096 (2014).

[143] Craine, J. M. et al. Global patterns of foliar nitrogen isotopes and their relationships with climate, mycorrhizal fungi, foliar nutrient concentrations, and nitrogen availability. *New Phytol.* **183**, 980–992 (2009). 10.1111/j.1469-8137.2009.02917.x 19563444

[144] Craine, J. M., Lee, W. G., Bond, W. J., Williams, R. J. & Johnson, L. C. Environmental constraints on a global relationship among leaf and root traits of grasses. *Ecology* **86**, 12–19 (2005).

[145] Craine, J. M. et al. Global diversity of drought tolerance and grassland climate-change resilience. *Nat. Clim. Change* **3**, 63–67 (2013).

[146] Diaz, S. et al. The plant traits that drive ecosystems: evidence from three continents. *J. Veg. Sci.* **15**, 295–304 (2004).

[147] Domingues, T. F., Martinelli, L. A. & Ehleringer, J. R. Ecophysiological traits of plant functional groups in forest and pasture ecosystems from eastern Amazônia, Brazil. *Plant Ecol.* **193**, 101–112 (2007).

[148] Fonseca, C. R., Overton, J. M., Collins, B. & Westoby, M. Shifts in trait-combinations along rainfall and phosphorus gradients. *J. Ecol.* **88**, 964–977 (2000).

[149] Fortunel, C. et al. Leaf traits capture the effects of land use changes and climate on litter decomposability of grasslands across Europe. *Ecology* **90**, 598–611 (2009). 10.1890/08-0418.1 19341132

[150] He, J.-S. et al. Leaf nitrogen:phosphorus stoichiometry across Chinese grassland biomes. *Oecologia* **155**, 301–310 (2008). 10.1007/s00442-007-0912-y 18278518

[151] Kattge, J., Knorr, W., Raddatz, T. & Wirth, C. Quantifying photosynthetic capacity and its relationship to leaf nitrogen content for global-scale terrestrial biosphere models. *Glob. Chang. Biol.* **15**, 976–991 (2009).

[152] Kazakou, E., Vile, D., Shipley, B., Gallet, C. & Garnier, E. Co-variations in litter decomposition, leaf traits and plant growth in species from a Mediterranean old-field succession. *Funct. Ecol.* **20**, 21–30 (2006).

[153] Kerkhoff, A. J., Fagan, W. F., Elser, J. J. & Enquist, B. J. Phylogenetic and growth form variation in the scaling of nitrogen and phosphorus in the seed plants. *Am. Nat.* **168**, E103–E122 (2006). 10.1086/507879 17004214

[154] Kleyer, M. et al. The LEDA Traitbase: a database of life-history traits of the Northwest European flora. *J. Ecol.* **96**, 1266–1274 (2008).

[155] Maire, V. et al. Global effects of soil and climate on leaf photosynthetic traits and rates. *Global Ecol. Biogeogr.* **24**, 706–717 (2015).

[156] McDonald, P. G., Fonseca, C. R., Overton, J. M. & Westoby, M. Leaf-size divergence along rainfall and soil-nutrient gradients: is the method of size reduction common among clades? *Funct. Ecol.* **17**, 50–57 (2003).

[157] Meziane, D. & Shipley, B. Interacting components of interspecific relative growth rate: constancy and change under differing conditions of light and nutrient supply. *Funct. Ecol.* **13**, 611–622 (1999).

[158] Onoda, Y. et al. Physiological and structural tradeoffs underlying the leaf economics spectrum. *New Phytol.* **214**, 1447–1463 (2017). 10.1111/nph.14496 28295374

[159] Pakeman, R. J. et al. Relative climatic, edaphic and management controls of plant functional trait signatures. *J. Veg. Sci.* **20**, 148–159 (2009).

[160] Peco, B., de Pablos, I., Traba, J. & Levassor, C. The effect of grazing abandonment on species composition and functional traits: the case of dehesa grasslands. *Basic Appl. Ecol.* **6**, 175–183 (2005).

[161] Prentice, I. C. et al. Evidence of a universal scaling relationship for leaf CO_2_ drawdown along an aridity gradient. *New Phytol.* **190**, 169–180 (2011). 10.1111/j.1469-8137.2010.03579.x 21182527

[162] Reich, P. B., Oleksyn, J. & Wright, I. J. Leaf phosphorus influences the photosynthesis-nitrogen relation: a cross-biome analysis of 314 species. *Oecologia* **160**, 207–212 (2009). 10.1007/s00442-009-1291-3 19212782

[163] Shipley, B. & Vu, T.-T. Dry matter content as a measure of dry matter concentration in plants and their parts. *New Phytol.* **153**, 359–364 (2002).

[164] Tucker, S. S., Craine, J. M. & Nippert, J. B. Physiological drought tolerance and the structuring of tallgrass prairie assemblages. *Ecosphere* **2**, art48 (2011).

[165] Wang, H. et al. The China Plant Trait Database: toward a comprehensive regional compilation of functional traits for land plants. *Ecology* **99**, 500 (2018). 10.1002/ecy.2091 29155446

[166] Wright, I. J. et al. The worldwide leaf economics spectrum. *Nature* **428**, 821–827 (2004). 10.1038/nature02403 15103368

[167] Bates, D., Mächler, M., Bolker, B. & Walker, S. Fitting linear mixed-effects models using lme4. *J. Stat. Softw.* **67**, 1–48 (2015).

[168] Liu, Y. et al. Evaluating the responses of net primary productivity and carbon use efficiency of global grassland to climate variability along an aridity gradient. *Sci. Total Environ.* **652**, 671–682 (2019). 10.1016/j.scitotenv.2018.10.295 30380475

[169] Simpson, K. J. et al. C_4_ photosynthesis and the economic spectra of leaf and root traits independently influence growth rates in grasses. *J. Ecol.* **108**, 1899–1909 (2020).

[170] Danabasoglu, G. et al. The Community Earth System Model version 2 (CESM2). *J. Adv. Model. Earth Syst.* **12**, e2019MS001916 (2020).

[171] Levis, S., Badger, A., Drewniak, B., Nevison, C. & Ren, X. CLM crop yields and water requirements: avoided impacts by choosing RCP 4.5 over 8.5. *Clim. Change* **146**, 501–515 (2018).

[172] Portmann, F. T., Siebert, S. & Döll, P. MIRCA2000—global monthly irrigated and rainfed crop areas around the year 2000: a new high-resolution data set for agricultural and hydrological modeling. *Global Biogeochem. Cycles* 10.1029/2008GB003435 (2010).

[173] Bonan, G. B. et al. Improving canopy processes in the Community Land Model (CLM4) using global flux fields empirically inferred from FLUXNET data. *J. Geophys. Res.* **116**, G02014 (2011).

[174] De Kauwe, M. G. et al. A test of an optimal stomatal conductance scheme within the CABLE land surface model. *Geosci. Model Dev.* **8**, 431–452 (2015).

[175] Shi, M., Fisher, J. B., Brzostek, E. R. & Phillips, R. P. Carbon cost of plant nitrogen acquisition: global carbon cycle impact from an improved plant nitrogen cycle in the Community Land Model. *Glob. Chang. Biol.* **22**, 1299–1314 (2016). 10.1111/gcb.13131 26473512

[176] Li, F., Zeng, X. D. & Levis, S. A process-based fire parameterization of intermediate complexity in a dynamic global vegetation model. *Biogeosciences* **9**, 2761–2780 (2012).

[177] Li, F., Levis, S. & Ward, D. S. Quantifying the role of fire in the Earth system—part 1: improved global fire modeling in the Community Earth System Model (CESM1). *Biogeosciences* **10**, 2293–2314 (2013).

[178] Umair, M., Kim, D. & Choi, M. Impact of climate, rising atmospheric carbon dioxide, and other environmental factors on water-use efficiency at multiple land cover types. *Sci. Rep.* **10**, 11644 (2020). 10.1038/s41598-020-68472-7 PMC7363916 32669589

[179] Lawrence, D. M., Koven, C. D., Swenson, S. C., Riley, W. J. & Slater, A. G. Permafrost thaw and resulting soil moisture changes regulate projected high-latitude CO_2_ and CH_4_ emissions. *Environ. Res. Lett.* **10**, 094011 (2015).

[180] Denager, T. et al. Point-scale multi-objective calibration of the Community Land Model (version 5.0) using in situ observations of water and energy fluxes and variables. *Hydrol. Earth Syst. Sci.* **27**, 2827–2845 (2023).

[181] Oloruntoba, B. J., Kollet, S., Montzka, C., Vereecken, H. & Hendricks Franssen, H.-J. High resolution land surface modelling over Africa: the role of uncertain soil properties in combination with temporal model resolution. *Hydrol. Earth Syst. Sci.* **29**, 1659–1683 (2025).

