# Supplementary material for: Increasing CO2 levels fertilize C4 grass production
Source: Nature. 2026 Aug 19;658(8135):415–20. doi: 10.1038/s41586-026-10935-4 (PMC13645630; doi:10.1038/s41586-026-10935-4)
Supplement: Supplementary file 1 [file 41586_2026_10935_MOESM1_ESM.pdf]

nature portfolio

https://doi.org/10.1038/s41586-026-10935-4

Supplementary information

Increasing CO_2 levels fertilize C_4 grass production

In the format provided by the authors and unedited

Supplementary information

Increasing CO_2 levels fertilize C_4 grass production

Kimberley J. Simpson[1,2]*, A. Carla Staver[3]*, James A. King[1], William J. Bond[4], Corli Coetsee[5,6], Nita C. M. Pallett[2], Adam F. A. Pellegrini[7], Sarah L. Raubenheimer[2,8], Brad S. Ripley[2], Maria Val Martin[1], Colin P. Osborne[1]

[1]Plants, Photosynthesis and Soil, School of Biosciences, University of Sheffield; Sheffield, UK.

[2]Botany Department, Rhodes University; Makhanda, South Africa.

[3]Department of Ecology and Evolutionary Biology, Princeton University; Princeton, USA.

[4]Department of Biological Sciences, University of Cape Town; Cape Town, South Africa.

[5]Scientific Services, South African National Parks; Skukuza, South Africa.

[6]School of Natural Resource Management, Nelson Mandela University; George, South Africa.

[7]Earth System Science Department, Stanford University; Stanford, USA.

[8]Institute for Global Change Biology, School for Environment and Sustainability, University of Michigan; Ann Arbor, USA.

* These authors contributed equally to this work

Table of Contents

Supplementary figures

Supplementary Figure 1 | Locations of long-term empirical observations in Kruger National Park, South Africa. …………………………………………………………………………3
Supplementary Figure 2 | Herbivore biomass in Kruger National Park through time…………………………………………………………………………………….....4
Supplementary Figure 3 | C_4 grass leaf nitrogen through time………………………………5
Supplementary Figure 4 | The modelled relationship between the maximum carboxylation rate of photosynthesis and leaf temperature for C_4 plants………………………………………6
Supplementary Figure 5 | The climatic distribution of the C_4 grass study species……………7
Supplementary Figure 6 | Meta-analysis results using the relativised beta-factor metric……………………………………………………………………………………..8
Supplementary Figure 7 | Funnel plots of plant trait response ratio data. ……………………9
Supplementary Figure 8 | Disc pasture meter calibration for Kruger National Park………………………………………………………………………….............10

Supplementary tables

Supplementary Table 1 | Model selection for *in situ* grass biomass observations…………...11
Supplementary Table 2 | Effect size estimates for *in situ* grass biomass observations……………………………………………………………………………..12
Supplementary Table 3 | Effect size estimates for *in situ* grass species relative abundance and community-weighted trait mean observations…………..………...13
Supplementary Table 4 | Model selection for *in situ* fire occurrence and parkwide burned area……………………………………………………………………….........................14
Supplementary Table 5 | Effect size estimates for *in situ* fire occurrence and parkwide burned area………………………………………………………...15
Supplementary Table 6 | Studies used to analyse stomatal conductance and leaf water potential traits under CO_2 and water treatments……..……………………………................16
Supplementary Table 7 | Parameters used for hydraulic-stomatal modelling………………18

Supplementary Discussion

Fire Occurrence in Kruger National Park……………………………………………………19

Supplementary figures

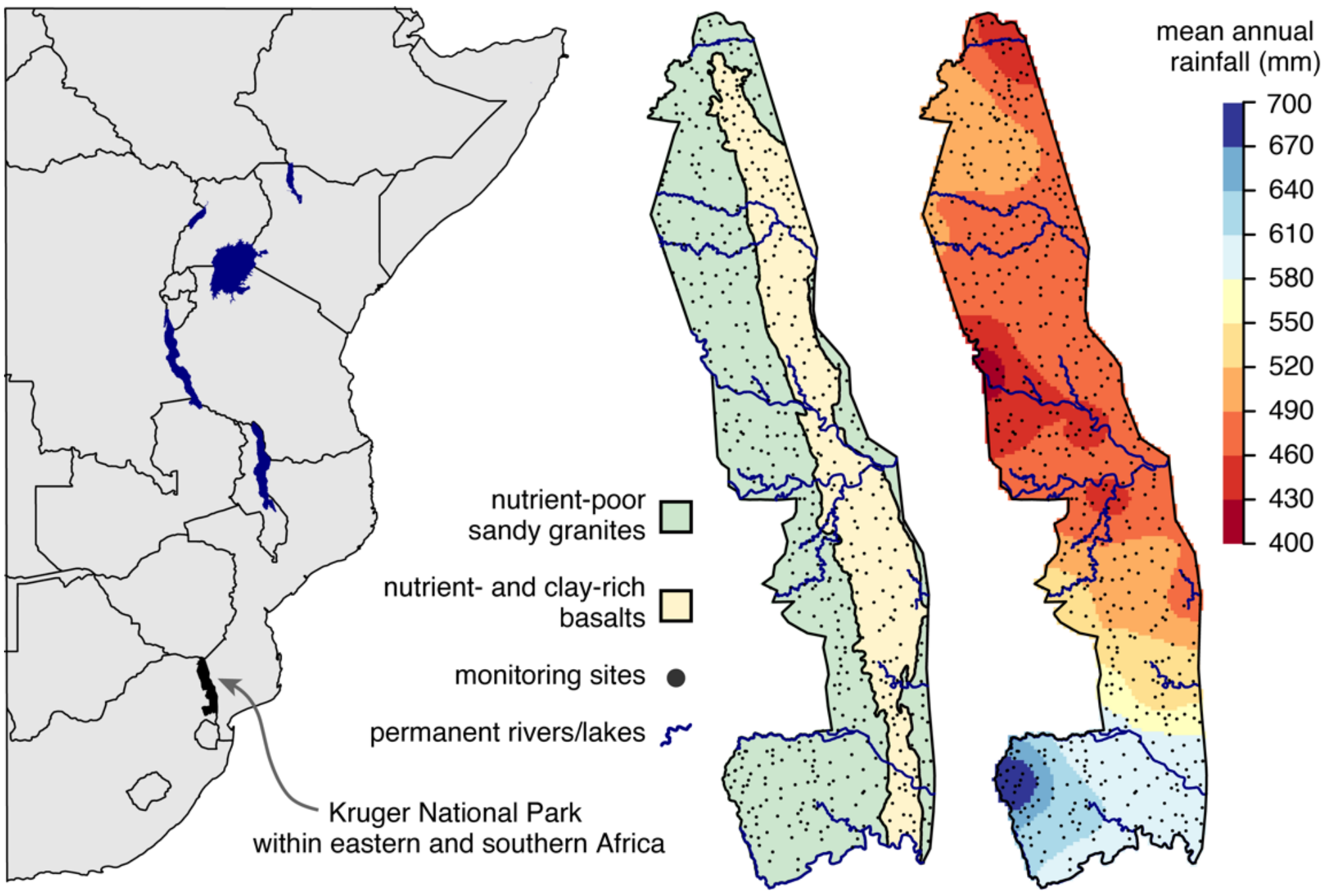

Figure S1. Locations of long-term empirical observations. Sites are shown within Kruger National Park, South Africa, with soil parent geology and mean annual rainfall gradients for context (from park management records as described in the Methods). GIS layers were supplied by South African National Parks (SANParks) and used with permission under a SANParks data user agreement.

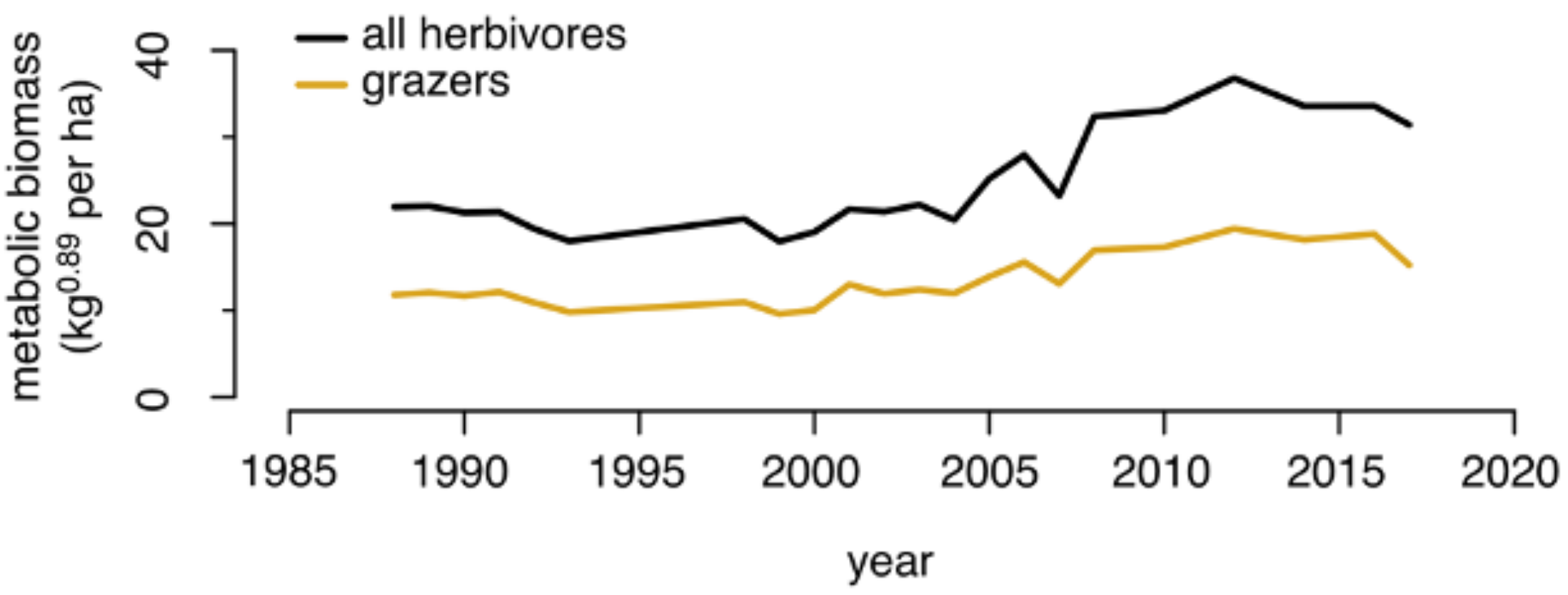

Figure S2. Herbivore biomass in Kruger National Park through time. Both grazer metabolic biomass density (R^2 = 0.610, N = 21, p < 0.001) and total-herbivore metabolic biomass density (R^2 = 0.625, N = 21, p < 0.001) have increased through times since the 1980s in the park, suggesting that declines in grazing pressure are probably not responsible for increases in grass biomass observed across the landscape.

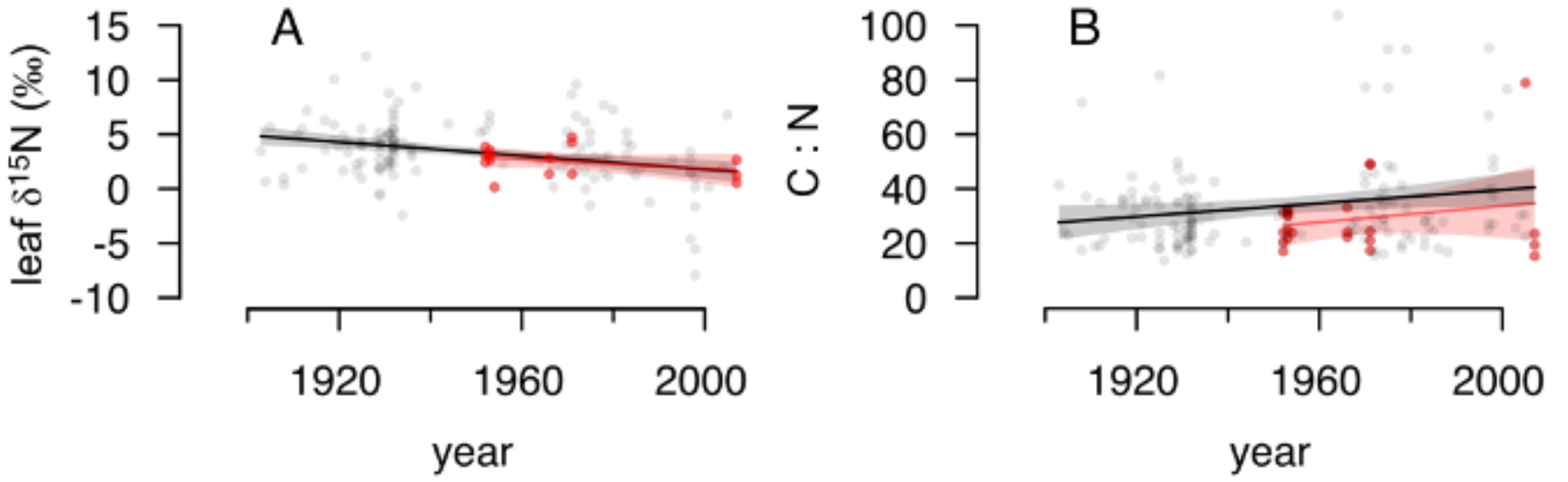

Figure S3. C_4 grass leaf nitrogen through time. Trends through time in grass leaf $\delta^{15}N$ (A) and grass leaf carbon-to-nitrogen ratios (B) from herbarium samples collected in Kruger National Park (red) and in South Africa (grey) from Ref. 25. In all South African C_4 grass samples, grass leaf $\delta^{15}N$ tended to decrease slightly ($R^2 = 0.104$, $N = 148$, $p < 0.001$) whereas C:N tended to increase slightly through time ($R^2 = 0.028$, $N = 177$, $p = 0.026$), both of which are consistent with progressive nitrogen limitation rather than with nitrogen deposition/fertilization. Trends in the Kruger samples alone were not significant, likely due to small sample sizes, but trends parallel those observed in South Africa ($\delta^{15}N$: $R^2 = 0.113$, $N = 15$, $p = 0.22$; C:N: $R^2 = 0.045$, $N = 25$, $p = 0.33$).

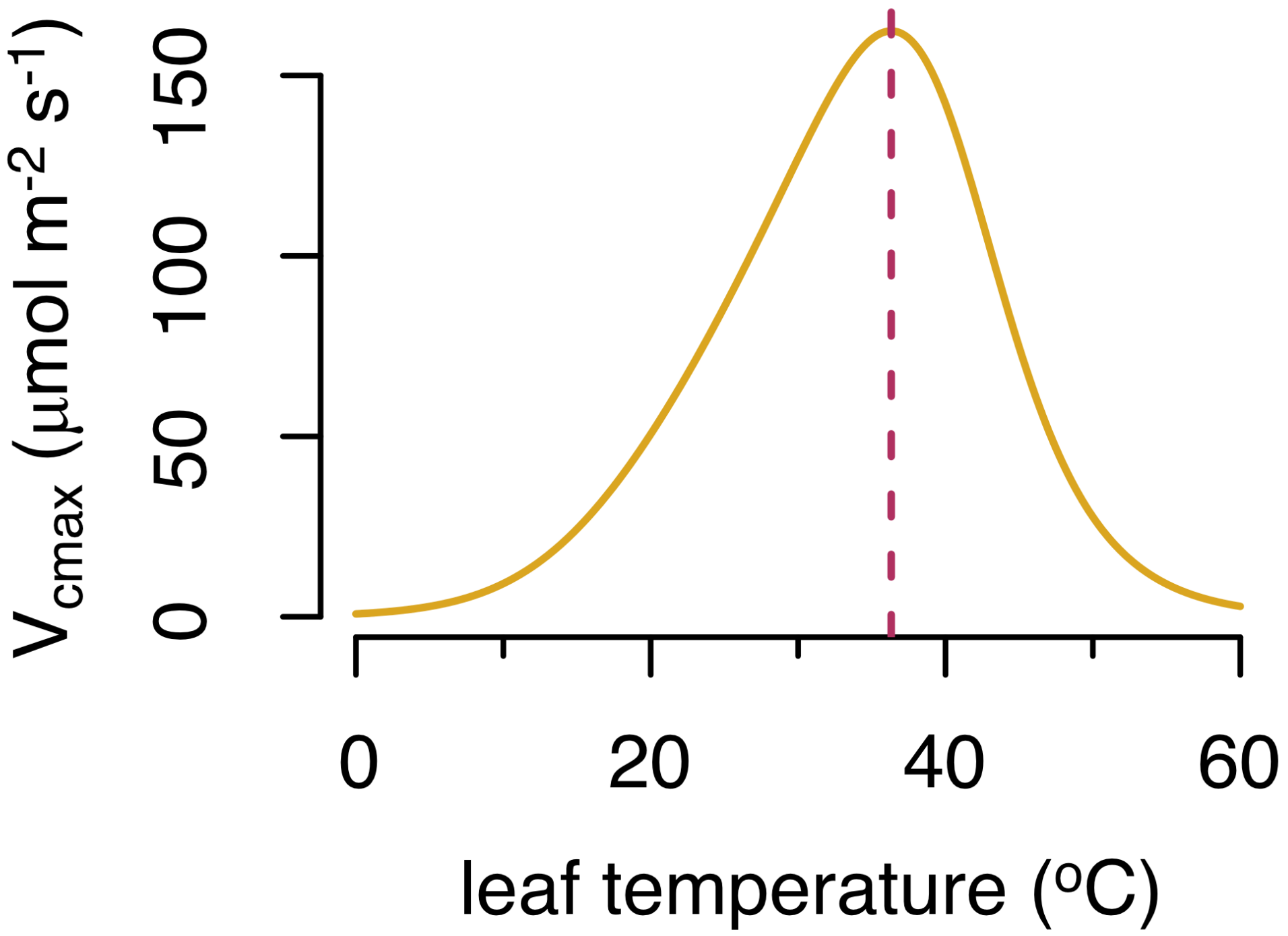

Figure S4. The relationship between the maximum carboxylation rate of photosynthesis (V_{cmax}) and leaf temperature for C_4 plants implemented in the Community Land Model 5[6]. The highest V_{cmax}, indicated by the dashed line, occurs at 36.3ºC (following ref. 174).

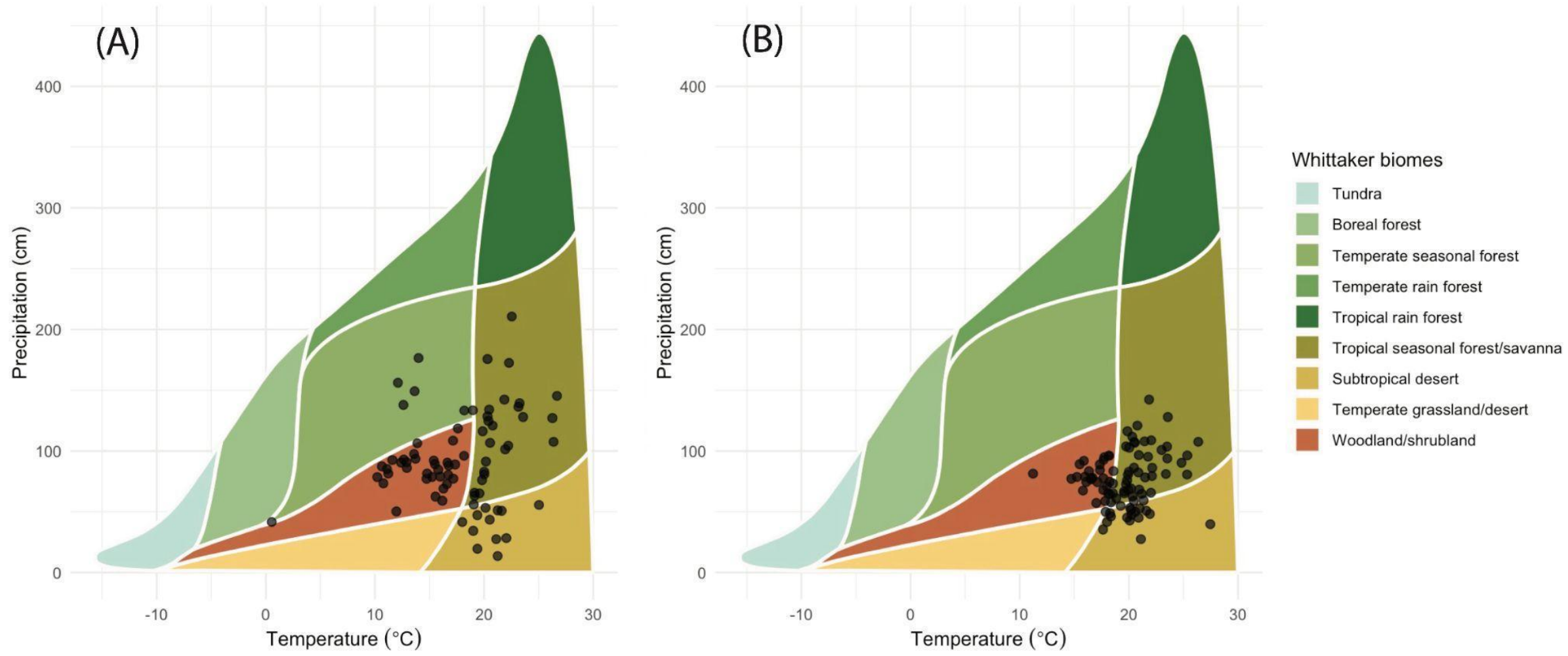

Figure S5. The climatic distribution of the C_4 grass study species. The species present in the meta-analysis (A) and the field plot data from Kruger National Park (B) are each represented by a single point superimposed onto a Whittaker biome plot. To determine the climatic niche of each grass species, we extracted georeferenced occurrence records from the Global Biodiversity Information Facility (GBIF) web portal (GBIF.org; GBIF Occurrence Download, DOI: 10.15468/dl.rckugp) and cleaned these to improve data quality (following ref. 182). Values were extracted for each occurrence point for two Worldclim products (WORLDCLIM version 2; ref. 183; accessed at a 30-s spatial resolution) and summarised as the mean for each species across the geographical area represented by the occurrence data.

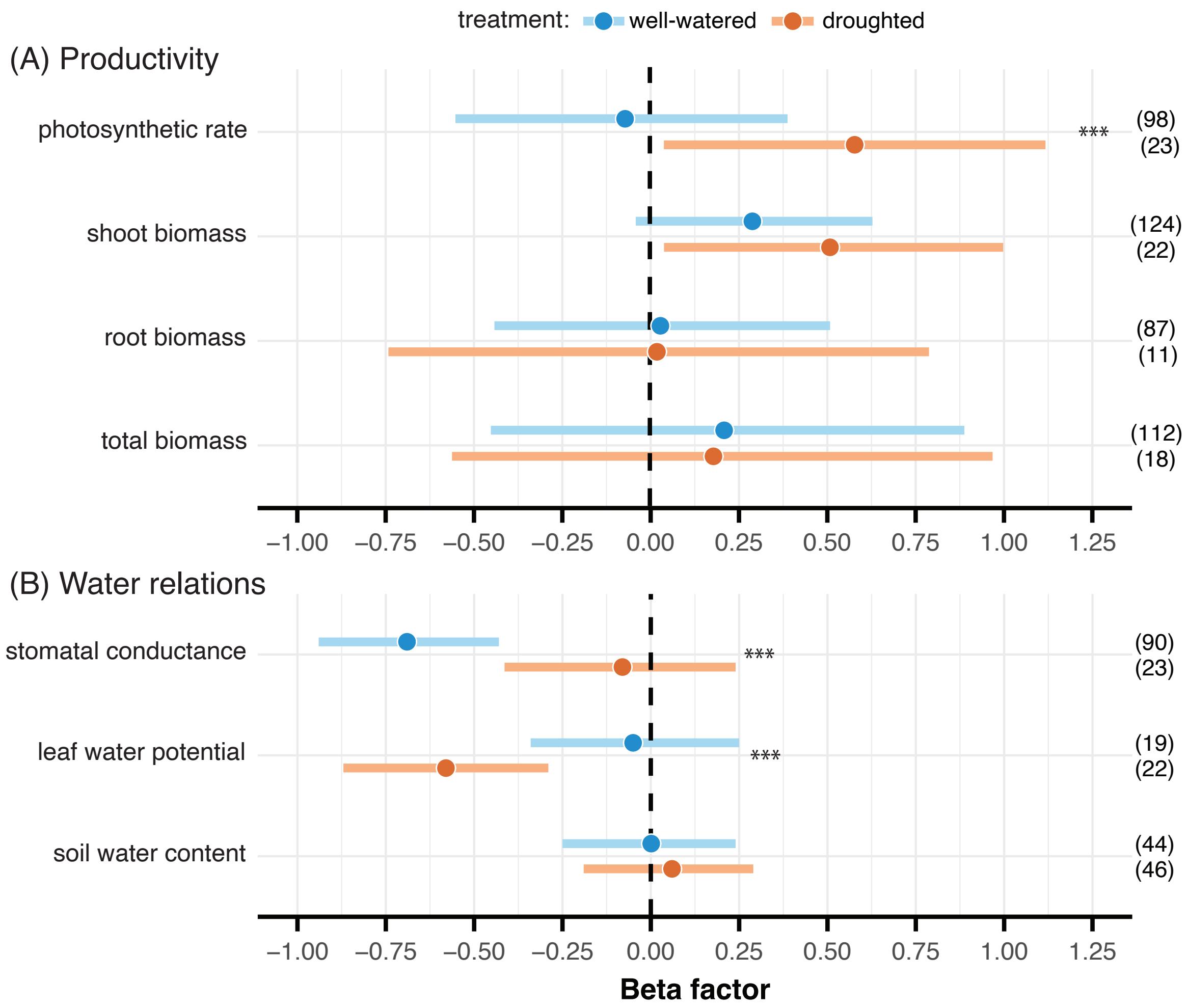

Figure S6. Meta-analysis of wild C_4 grass responses to elevated CO_2 under different water availability using the relativised beta-factor metric. Responses of grass productivity traits (**A**) and water-relation traits (B) are displayed using the relativised beta factor metric, which standardises CO_2 effects on traits by the change in CO_2 concentration (calculated from equation 1 in ref. 130). For this metric, $\beta = 1$ represents direct proportionality between a variable's CO_2 response and the change in CO_2. Points represent estimated marginal means and bars show the 95% confidence intervals of relativised beta factors. Significant CO_2 effects are indicated by beta-factor 95% confidence intervals that do not overlap zero. Asterisks show significant differences between drought and well-watered treatments (* $P<0.05$; ** $P<0.01$; *** $P<0.001$). Sample sizes are given in brackets.

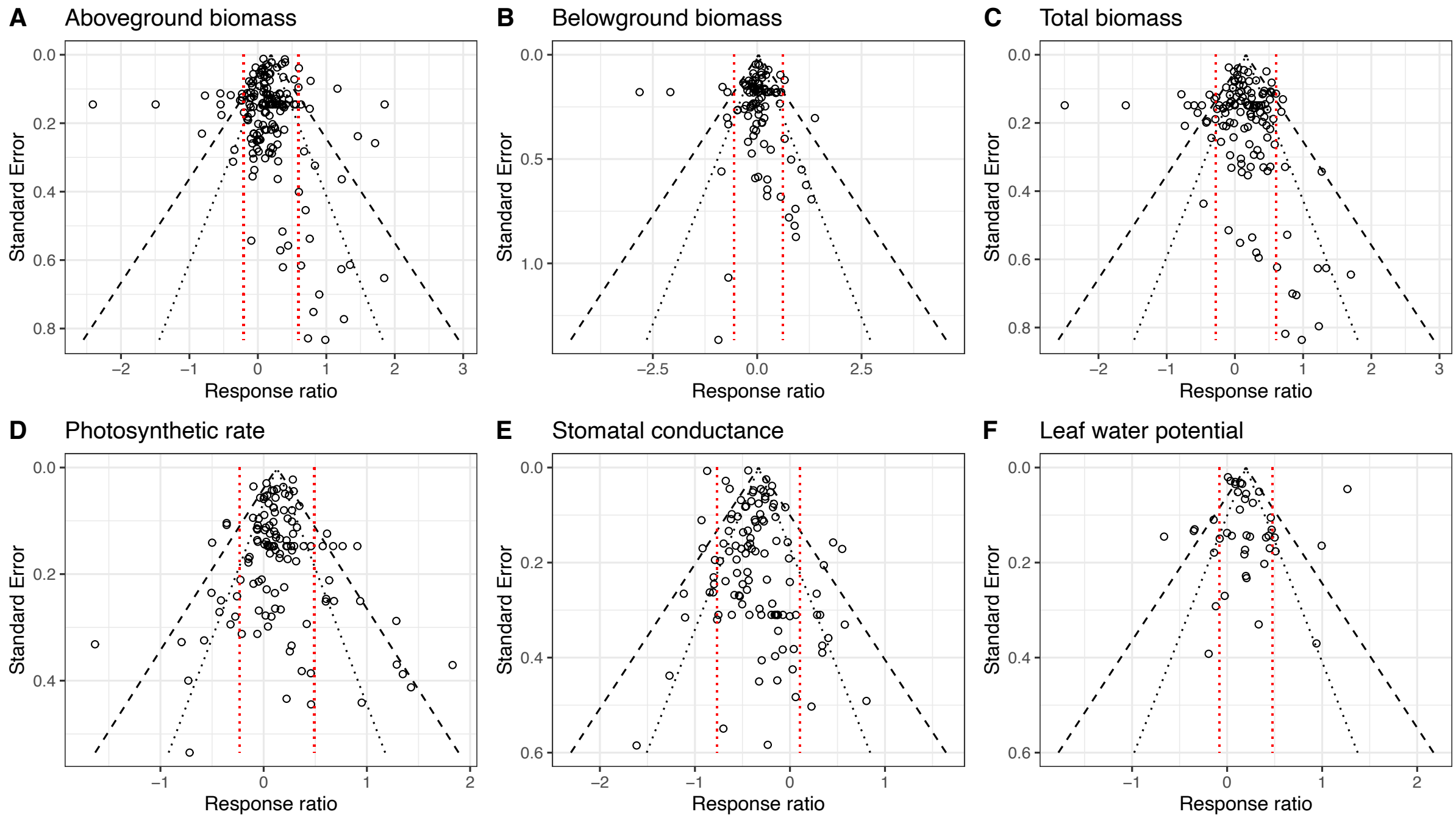

Figure S7. Funnel plots of plant trait response ratio data. Plots show the inverse standard error for variables shown in Figure 1, except soil water content where a measure of standard error was rarely provided. Black dotted and dashed lines show limits of the 95% and 99% confidence intervals respectively, and the red dotted line in the mean response ratio 95% confidence interval.

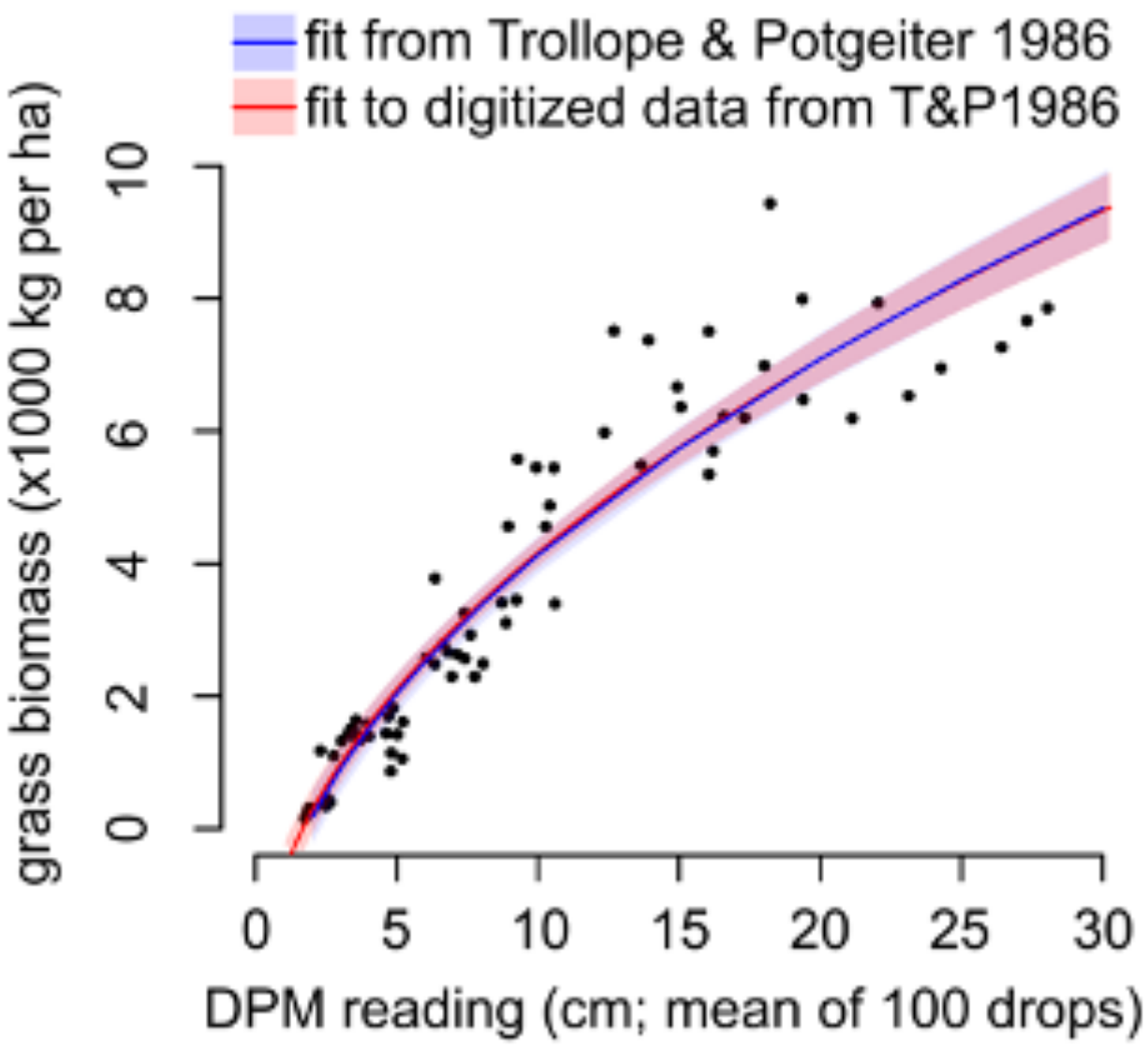

Figure S8. Disc pasture meter (DPM) calibration for Kruger National Park from Trollope and Potgieter (1986)[135]. Raw data are no longer available, but the original paper published a calibration line including errors, and data digitized from the published plots closely match the original model fit. The fit shown is for clipped grass biomass versus the average of 100 DPM drops, which closely matches the 104 DPM drops per plot used in the field dataset included in this paper. We ran 100 randomizations to check whether errors associated with the use of the DPM might change statistical results reported in Tables S1-S2 but found that the same reported model was selected for every run.

Supplementary tables

Table S1. Model selection for *in situ* grass biomass observations. Grass biomass was modelled using linear mixed effects models, with plot as a random effect, in response to year, parent geologic type ('geology': nutrient-poor, sandy, granite-derived or nutrient-rich, clayey, basalt-derived soils), annual rainfall ('rain': July-June to capture a January-April wet season, where grass biomass was sampled in April), previous year rainfall, number of total rainfall events (July-June), mean daily temperature (July-June), time since fire ('TSF', in years, log-transformed) and year, including interactions between year and annual rainfall and geology and annual rainfall. Data were not scaled before modelling. Top models for each data subset (the simplest model among those within two AIC units of the model with the lowest AIC, shown in bold) are similar irrespective of data subset and whether species relative abundances or trait community-weighted means (CWMs) were included as explanatory variables. The best model for the full biomass dataset is highlighted in grey across data subsets, for which model results are shown in Table S2 and reported in the main text.

Data subset	model	ΔAIC
grass biomass [all data, *N* = 10,498]	***~ year × geology + rain + previous rain + rain events + temperature +* ln(*TSF*)**	***0***
	~ year × (geology + rain) + previous rain + rain events + temperature + ln(TSF)	3.67
grass biomass [no lawns[*], *N* = 7,544]	**~ year × (geology + rain) + prev. rain + rain events + temperature + ln(TSF)**	**0**
	~ year × (geology + rain) + previous rain + rain events + ln(TSF)	8.97
	~ year × geology + rain + previous rain + rain events + temperature + ln(*TSF*)	*9.74*
grass biomass [plots with species comp[†], *N* = 9,490]	~ year × (geology + rain) + previous rain + rain events + temperature + ln(TSF) + Uroc mos + Both rad + Digi eri + Them tri + Mega max	0
	~ year × geology + rain + previous rain + rain events + temperature +* ln(*TSF*) *+ Uroc mos + Both rad + Digi eri + Them tri + Mega max	***1.26***
grass biomass [trait plots[‡], *N* = 4,522]	***~ year × geology + rain + previous rain + rain events + temperature +* ln(*TSF*) *+ SLA$_{CWM}$ + max height$_{CWM}$***	***0***
	~ year × (geology + rain) + previous rain + rain events + temperature + ln(TSF) + SLA$_{CWM}$ + max height$_{CWM}$	7.25

[*] Here, lawns are defined as having > 20% relative abundance of three possible lawn-forming grass species: *Urochloa mosambicensis*, *Digitaria eriantha*, *Megathyrsus coloratum*

[†] Ordination revealed no major axes of community organization not captured by individual species abundances. Only species with an average relative abundance > 10% were included as variables. *Uroc mos = Urochloa mosambicensis*; *Both rad = Bothriochloa radicans*; *Digi eri = Digitaria eriantha*; *Them tri = Themeda triandra*; *Mega max = Megathyrsus maximus*.

[‡] Trait plots have > 80% coverage by relative abundance of SLA, used to calculate community weighted means by sample. Only SLA and maximum species height were used here because *gs* coverage was low.

Table S2. Effect size estimates for *in situ* grass biomass observations. Linear mixed effects models, with plot as a random effect, were used. Explanatory variables were: year, parent geologic type ('geology': nutrient-poor, sandy, granite-derived or nutrient-rich, clayey, basalt-derived soils; effects reported are granite compared to a basalt baseline), annual rainfall ('rain' in mm per year: July-June to capture a January-April wet season, where grass biomass was sampled in April), previous year rainfall (mm per year), number of total rainfall events (July-June), mean daily maximum temperature (°C; July-June), time since fire (in years, log-transformed), and interactions between year and annual rainfall, and geology and annual rainfall. Grass biomass is reported in units of g m^{-2}.

data subset	grass biomass ~									
	Intercept	year	geology°	year × geology	rain	previous year rain	rain events	temperature	ln(time since fire)	other variables
[all data, *N* = 10,498]	-7,253 ± 518	3.66 ± 0.28	3,973 ± 580	-2.03 ± 0.29	0.518 ± 0.011	0.183 ± 0.009	-5.02 ± 0.49	-0.717 ± 2.48	9.36 ± 1.57	
[no lawns*, *N* = 7,544]	-9,193 ± 701	4.70 ± 0.37	5,632 ± 770	-2.86 ± 0.38	0.521 ± 0.013	0.170 ± 0.010	-6.24 ± 0.62	-5.22 ± 3.12	8.94 ± 1.87	
[plots with species comp†, *N* = 9,490]	-6,393 ± 569	3.20 ± 0.30	3,407 ± 644	-1.74 ± 0.32	0.531 ± 0.011	0.172 ± 0.009	-5.81 ± 0.53	-0.035 ± 2.71	10.4 ± 1.6	+ *Uroc mos* + *Both rad* + *Digi eri* + *Them tri* + *Mega max*
[trait plots‡, *N* = 4,522]	-7,504 ± 829	3.86 ± 0.44	5,762 ± 918	-2.92 ± 0.46	0.524 ± 0.017	0.141 ± 0.014	-5.44 ± 0.82	-8.58 ± 4.11	18.4 ± 2.5	+ SLA_{CWM} + max $height_{CWM}$

* Here, lawns are defined as having > 20% relative abundance of three lawn-forming grass species: *Urochloa mosambicensis*, *Digitaria eriantha*, *Megathyrsus coloratum*.

† Ordination revealed no major axes of community organization. Only species with on average relative abundance > 10% were included as variables. *Uroc mos* = *Urochloa mosambicensis*; *Both rad* = *Bothriochloa radicans*; *Digi eri* = *Digitaria eriantha*; *Them tri* = *Themeda triandra*; *Mega max* = *Megathyrsus maximus*.

‡ Trait plots have > 80% coverage by relative abundance of SLA, used to calculate community weighted means by sample. Only SLA and maximum species height were used here because *gs* coverage was low.

° Geology effect size here should be interpreted only after including the interaction with year. At the midpoint of the observation period in 2005, basalt-derived, clayey, nutrient-rich soils had on average 98.7 ± 9.2, 104.5 ± 10.1, 86.1 ± 9.1, 100.0 ± 9.9 g m^{-2} more grass biomass than granite-derived soils for the four models listed here.

Table S3. Effect size estimates for *in situ* grass species relative abundance and community weighted trait mean observations. Responses were modelled using linear mixed effects models, with plot as a random effect, in response to year, parent geologic type ('geology': nutrient-poor, sandy, granite-derived or nutrient-rich, clayey, basalt-derived soils), annual rainfall ('rain': July-June to capture a January-April wet season, where grass biomass was sampled in April), previous year rainfall, number of total rainfall events (July-June), and mean daily temperature (July-June), including interactions between year and annual rainfall and geology and annual rainfall. Species relative abundances are modeled in units of percent. The same model is shown for each species and trait response irrespective of whether that is the 'best' model.

response variable	Intercept	year	geology°	year × geology	rain	previous year rain	rain events	temperature	ln (time since fire)
Urochloa mosambicensis	-66 ± 36	-0.030 ± 0.019	12.4 ± 41.1	-0.009 ± 0.021	-0.001 ± 0.000	0.004 ± 0.001	0.27 ± 0.03	0.40 ± 0.17	-0.032 ± 0.11
Bothriochloa radicans	-36 ± 47	0.062 ± 0.025	-325 ± 53	0.17 ± 0.03	-0.011 ± 0.000	-0.004 ± 0.001	0.11 ± 0.04	-2.54 ± 0.22	-1.46 ± 0.13
Digitaria eriantha	434 ± 33	-0.22 ± 0.02	-295 ± 38	0.15 ± 0.02	0.006 ± 0.001	-0.001 ± 0.001	-0.12 ± 0.03	0.20 ± 0.16	1.02 ± 0.10
Themeda triandra	-4.9 ± 8.2	0.003 ± 0.004	-4.7 ± 9.3	0.002 ± 0.005	0.000 ± 0.000	0.000 ± 0.000	0.02 ± 0.01	-0.034 ± 0.039	-0.047 ± 0.022
Megathyrsus maximum	-287 ± 30	0.16 ± 0.02	280 ± 34	-0.14 ± 0.02	0.000 ± 0.000	-0.003 ± 0.000	-0.12 ± 0.03	-0.47 ± 0.14	-0.065 ± 0.087
CWM: SLA	-254 ± 19	0.14 ± 0.01	105 ± 22	-0.053 ± 0.011	0.002 ± 0.000	0.001 ± 0.000	-0.072 ± 0.018	-0.54 ± 0.09	-0.44 ± 0.06
CWM: individual height	-1,655 ± 192	0.94 ± 0.10	535 ± 218	-0.27 ± 0.11	0.052 ± 0.004	0.038 ± 0.003	-0.50 ± 0.18	-4.83 ± 0.92	-4.51 ± 0.56
CWM: g_s	-614 ± 87	0.37 ± 0.05	-164 ± 100	0.073 ± 0.050	0.019 ± 0.002	0.008 ± 0.001	-0.64 ± 0.08	-2.72 ± 0.42	-1.43 ± 0.25

° Geology effect size here should be interpreted including the interaction with year. At the midpoint of the observation period in 2005, basalt soils had on average 6.24 ± 0.89, -6.21 ± 0.64, 3.52 ± 0.46, -0.18 ± 0.07, 5.88 ± 0.55 higher relative abundance (in %) of each of the 5 species listed here, than granites, controlling for all other modelled variables. SLA was 2.14 ± 0.35 (cm^2 g^{-1}), height was 4.70 ± 3.07 cm, and g_s was 16.2 ± 1.5 mmol m^{-2} s^{-1} higher on basalt than granite in 2005.

Table S4. Model selection for *in situ* fire occurrence and parkwide burned area. Model selection was performed on two different data formats. First, fire presence/absence at each grass biomass monitoring site through time, modelled using linear mixed effects models, with plot as a random effect; we constructed two base models for this analysis, one based on climate (including year, parent geologic type [‘geology’: nutrient-poor, sandy, granite-derived or nutrient-rich, clayey, basalt-derived soils], annual rainfall [‘rain’: July-June to capture a January-April wet season, where grass biomass was sampled in April], previous year rainfall, number of total rainfall events [July-June], mean daily temperature [July-June], with interactions between year and geology, and year and annual rainfall, to mirror our grass biomass model) and a second based only on year and grass biomass. Second, to extend burned area observations to include rainfall and temperature records available across three sites back to 1960, we calculated annual parkwide burned area as the proportion of monitoring sites that burned and then modelled this proportion as a function of year, rainfall, previous year rainfall, rainfall events, and temperature. Parkwide burned area, bounded between 0 and 1, was calculated at monitoring sites to make these comparable to site models, transformed assuming it is logistically distributed via $BA_{transformed} = -ln\left(\frac{1}{BA} - 1\right)$, then modeled linearly with years as replicates.

data subset	Model	ΔAIC
fire occurrence at monitoring sites, 1989-2020; *N = 15,590*	***~ year × geology + rain + previous year rain + temperature***	***0***
	~ year × geology + rain + previous year rain + temperature + rain events	2.00
	~ year + geology + rain + previous year rain + temperature	6.76
fire occurrence at monitoring sites, 1989-2020 *N = 10,396*	***~ grass***	***0***
	~ grass + year	1.70
	~ year	947.7
annual parkwide burned area, 1960-2020; *N = 60 years × 2 soil types = 120*	***~ rain + previous year rain***	***0***
	~ rain + previous year rain + temperature	1.43
	~ rain + previous year rain + geology	1.99
	~ rain + previous year rain + year	2.19

Table S5. Effect size estimates for *in situ* fire occurrence and parkwide burned area. Fire occurrence was modelled as a binomial response using linear mixed effects models, with plot as a random effect, in response to year, parent geologic type ('geology': nutrient-poor, sandy, granite-derived or nutrient-rich, clayey, basalt-derived soils, with effects reported as the effects of granite compared to a basalt baseline), annual rainfall ('rain': July-June to capture a January-April wet season, where grass biomass was sampled in April), previous year rainfall, number of total rainfall events (July-June), mean daily temperature (July-June), and year, including an interaction between geology and annual rainfall. Parkwide burned area, bounded between 0 and 1, was transformed assuming it is logistically distributed via $BA_{transformed} = -ln\left(\frac{1}{BA} - 1\right)$, then modeled linearly with years as replicates.

response variable	variable	effect size
fire occurrence, 1989-2020, [*N = 15,590*] ~	Intercept	-15.3 ± 1.0
	~ year	0.0088 ± 0.0002
	~ geology	-0.41 ± 0.07
	~ rain	0.0039 ± 0.0002
	~ previous year rain	0.0030 ± 0.0002
	~ temperature	-0.24 ± 0.03
fire occurrence, 1989-2020, [*N = 10,396*] ~	Intercept	-3.4 ± 0.1
	~ grass	0.00051 ± 0.00002
burned area, 1960-2020 [*N = 60 years x 2 soil types = 120*] ~	Intercept	-8.1 ± 1.0
	~ rain	0.0067 ± 0.0013
	~ previous year rain	0.0049 ± 0.0013

Table S6. Studies used to analyse stomatal conductance and leaf water potential traits under different CO_2 concentrations and water availabilities. Abbreviations used: SWC = soil water content (on a mass basis); VWC = volumetric water content.

Study	Ambient CO_2 (ppm)	Elevated CO_2 (ppm)	Water treatment details	Species
Adam et al. 2000 [57]	360	720	Measurements were made during a year with abundant rainfall (1993; 143.5 cm annual precipitation) and a year with below-normal rainfall (1994; 68.2 cm annual precipitation). The 30-year average annual precipitation is 85.9 cm. In the wet year, soil VWC remained ~40%. In the dry year, soil VWC declined to ~25 - 31% (differing by CO_2 treatment). Sample size: 35 (control) and 35 (drought) for both [CO_2]	*Andropogon gerardii*
Clark et al. 1999 [65]	350	700	Drought treatment was water being withheld from plants for 6 weeks. Soil VWC fell from ~0.60 $m^3\ m^{-3}$ to < 0.10 $m^3\ m^{-3}$ due to drought treatment Sample size: 12 (control) and 12 (drought) for both [CO_2]	*Paspalum dilatatum*
Fravolini et al. 2002 [69]	370	690	Plants were grown either with a 'high' water supply (watered to field capacity (500 ml) every 2 days), or a 'low' water supply where plants received 500 ml of water every 3 days. Sample size: 8 (control) and 8 (drought) for both [CO_2]	*Aristida californica, Bouteloua curtipendula, Eragrostis lehmanniana*
Hamerlynck et al. 1997 [75]	355	700	Plants were subject to three drying-wetting cycles during growing season (early, mid and late season). In the drying cycles, precipitation was reduced by 98-99% for a minimum of 14 days (until plants showed visible signs of water stress such as leaf curling). Sample size: 36 (control) and 36 (drought) for both [CO_2]	*Andropogon gerardii*
Knapp *et al.* 1993 [80]	350	700	Measurements were made during a year with abundant rainfall (1992; 3.0cm above the 30-year average annual precipitation of 23.8 cm) and a year with below-normal rainfall	*Andropogon gerardii*

			(1991; 6.7 cm below the 30-year average annual precipitation). Field measurements of average midday leaf ψ of plants outside the experimental chambers (and not subject to CO_2 treatments) were -2.38 MPa (in 1991) and -1.68 MPa (in 1992) Sample size: 12 (control; wet cycle) and 12 (drought; dry cycle) for both [CO_2]	
Pallett 2018 [95]	400	600, 800	SWC was maintained at ~17% in 'well-watered' control plants. In the 'drought' treatment, watering was reduced or stopped to achieve a ~10% SWC. Sample size: 4-8 (control) and 4-8 (drought) for all [CO_2]	*Heteropogon contortus, Hyparrhenia hirta, Paspalum urvillei, Themeda triandra, Tristachya leucothrix*
Pastore *et al*. 2020 [97]	400	580	A rainfall reduction treatment involved rainout shelters that achieved a ~45% rainfall reduction. In the reduced rainfall treatment, plants experienced soil VWC that was ~28% lower on average than those under ambient rainfall. Sample size: 48 (control) and 24 (drought) for both [CO_2]	*Andropogon gerardii*
Raubenheimer *et al.* 2022 [43]	400	800	Plants were either well-watered (daily watering) or exposed to four-week drought cycles (with no additional water provided), separated by two-day watered recovery periods. After 24 weeks, the mean soil water content was ~24% and ~9% for watered and drought treatments, respectively. Sample size: 17 (control) and 17 (drought) for both [CO_2]	*Themeda triandra*

Table S7. Parameters used for hydraulic-stomatal modelling. Values of K_{max}, Gs_{max}, P_{K50} and P_{Gs50} are for a widespread C_4 savanna grass species, *Themeda triandra*.

Parameter	Units	C_4 grass value (ambient, elevated CO_2)	Source
K_{max}	mmol m^{-2} s^{-1} MPa^{-1}	5.4, 5.4	Jacob et al 2022 [131]
Gs_{max}	mmol m^{-2} s^{-1}	130, 97	Values are estimated marginal means of *Gs* for plants grown in well-watered conditions under ambient CO_2 (121 mmol m^{-2} s^{-1}) and elevated CO_2 (88 mmol m^{-2} s^{-1}), Estimates were based on raw data from studies examining stomatal conductance and leaf water potential across different CO_2 concentrations and water availabilities (Table S6). Both values were adjusted upward by 10% because the mean estimates were not measured at LWP = 0.
P_{Gs50}	MPa	-2.8, -2.8	Jacob et al 2022 [131]
P_{K50}	MPa	-2.1, -2.1	Jacob et al 2022 [131]
c	MPa	0.5, 0.5	A common value for both equations A2 and A3 was chosen that provided a reasonable fit to the meta-analysis data

Supplementary Discussion

Fire Occurrence in Kruger National Park. The probability of fire occurrence at Kruger was higher with greater grass biomass and thus also with higher rainfall and lower temperatures (Tables S4 and S5). A recent analysis shows that this relationship arises because higher grass biomass in wetter years leads to greater fuel connectivity[184]. The probability of fire increased slightly with respect to climatological variables throughout the study period to mirror increases in grass biomass (increasing by 1.41 times overall from 12.1% to 17.0%; see Extended Data Fig. 10). Consistent with this explanation, accounting for grass biomass directly removed the effect of year, suggesting that grass biomass increases were directly responsible for slight increases in probability of fire occurrence. Despite a longer observation period, this result was not detectable in burned area increases at the park scale even when controlling for rainfall (see Tables S4 and S5), likely because effects were so small and variable. In sum, the data indicate that fire occurrence is associated with climate and that, for equivalent climate, fire activity may be increasing, likely due to increasing grass biomass, but there is only weak support for a feedback on fire mediated via the CO_2 fertilization of grass biomass.

References

[References 1-181 can be found in the main text document]

182. Simpson, K. J. et al. Bigger genomes provide environment-dependent growth benefits in grasses. *New Phytol.* **244**, 2049–2061 (2024).

183. Fick, S. E. & Hijmans, R. J. WorldClim 2: new 1-km spatial resolution climate surfaces for global land areas. *Int. J. Climatol*. **37,** 4302–4315 (2017).

184. Cardoso, A. W. et al. Quantifying the environmental limits to fire spread in grassy ecosystems. *Proc. Natl Acad. Sci. USA* **119**, e2110364119 (2022).
