# Supplementary material for: Increasing CO2 levels fertilize C4 grass production
Source: Nature. 2026 Aug 19;658(8135):415–20. doi: 10.1038/s41586-026-10935-4 (PMC13645630; doi:10.1038/s41586-026-10935-4)
Supplement: Supplementary file 2 [file 41586_2026_10935_MOESM2_ESM.pdf]

nature portfolio

Corresponding author(s): KJ Simpson

Last updated by author(s): Jul 9, 2026

# Reporting Summary

Nature Portfolio wishes to improve the reproducibility of the work that we publish. This form provides structure for consistency and transparency in reporting. For further information on Nature Portfolio policies, see our Editorial Policies and the Editorial Policy Checklist.

## Statistics

For all statistical analyses, confirm that the following items are present in the figure legend, table legend, main text, or Methods section.

| n/a | Confirmed | |
|---|---|---|
| ☐ | ☒ | The exact sample size (*n*) for each experimental group/condition, given as a discrete number and unit of measurement |
| ☐ | ☒ | A statement on whether measurements were taken from distinct samples or whether the same sample was measured repeatedly |
| ☐ | ☒ | The statistical test(s) used AND whether they are one- or two-sided<br>*Only common tests should be described solely by name; describe more complex techniques in the Methods section.* |
| ☐ | ☒ | A description of all covariates tested |
| ☐ | ☒ | A description of any assumptions or corrections, such as tests of normality and adjustment for multiple comparisons |
| ☐ | ☒ | A full description of the statistical parameters including central tendency (e.g. means) or other basic estimates (e.g. regression coefficient) AND variation (e.g. standard deviation) or associated estimates of uncertainty (e.g. confidence intervals) |
| ☐ | ☒ | For null hypothesis testing, the test statistic (e.g. *F*, *t*, *r*) with confidence intervals, effect sizes, degrees of freedom and *P* value noted<br>*Give P values as exact values whenever suitable.* |
| ☐ | ☒ | For Bayesian analysis, information on the choice of priors and Markov chain Monte Carlo settings |
| ☒ | ☐ | For hierarchical and complex designs, identification of the appropriate level for tests and full reporting of outcomes |
| ☐ | ☒ | Estimates of effect sizes (e.g. Cohen's *d*, Pearson's *r*), indicating how they were calculated |

*Our web collection on statistics for biologists contains articles on many of the points above.*

## Software and code

Policy information about availability of computer code

| | |
|---|---|
| Data collection | To simulate grass biomass trends into the future under climate change, we used the Community Land Model (CLM) version 5, which is the terrestrial component of the Community Earth System Model version 2. CLM5.0 is publicly available through the Community Terrestrial System Model (CTSM) git repository.<br>To collect data on C4 grass responses to CO2 addition from the published literature, the 'Litsearchr' R package (V.1.0) was used to create search terms.<br>Where necessary, data was manually digitized from published manuscipts using WebPlotDigitizer v.4.6. |
| Data analysis | All analyses were undertaken using the R language and platform (version 4.3.2). Key packages used include 'MCMCglmm'(v.2.36), 'coda' (v.0.19.4.1), 'lme4' (v.1.1.38), and 'Emmeans' (v.1.11.1). |

For manuscripts utilizing custom algorithms or software that are central to the research but not yet described in published literature, software must be made available to editors and reviewers. We strongly encourage code deposition in a community repository (e.g. GitHub). See the Nature Portfolio guidelines for submitting code & software for further information.

## Data

Policy information about availability of data

All manuscripts must include a data availability statement. This statement should provide the following information, where applicable:

- Accession codes, unique identifiers, or web links for publicly available datasets
- A description of any restrictions on data availability
- For clinical datasets or third party data, please ensure that the statement adheres to our policy

The data supporting the findings of this study are available from the Dryad Digital Repository at https://doi.org/10.5061/dryad.rbnzs7hq7. The meta-analysis was informed by literature searches conducted using the following databases and repositories: Web of Science (https://clarivate.com/academia-government/scientific-and-academic-research/research-discovery-and-referencing/web-of-science/), Scopus (https://www.elsevier.com/en-gb/products/scopus/search), the White Rose Research Online repository (https://eprints.whiterose.ac.uk/), and the South African National ETD Portal for theses and dissertations (https://spu-za.libguides.com/az/national-etd-portal-south-african-theses-and-dissertations).
Trait values for common grass species found in the Veld Condition Assessment plots at Kruger National Park were extracted from the TRY database (https://www.try-db.org/TryWeb/Home.php). Citations for the TRY database and the original publications are provided.

## Research involving human participants, their data, or biological material

Policy information about studies with human participants or human data. See also policy information about sex, gender (identity/presentation), and sexual orientation and race, ethnicity and racism.

| | |
|---|---|
| Reporting on sex and gender | N/A |
| Reporting on race, ethnicity, or other socially relevant groupings | N/A |
| Population characteristics | N/A |
| Recruitment | N/A |
| Ethics oversight | N/A |

Note that full information on the approval of the study protocol must also be provided in the manuscript.

# Field-specific reporting

Please select the one below that is the best fit for your research. If you are not sure, read the appropriate sections before making your selection.

☐ Life sciences ☐ Behavioural & social sciences ☒ Ecological, evolutionary & environmental sciences

For a reference copy of the document with all sections, see nature.com/documents/nr-reporting-summary-flat.pdf

# Ecological, evolutionary & environmental sciences study design

All studies must disclose on these points even when the disclosure is negative.

| | |
|---|---|
| Study description | An analysis of C4 grass responses to increasing atmospheric CO2 concentrations. This study combines three complementary approaches - 1) a meta-analysis of published C4 grass responses under CO2-addition experiments, 2) in situ field observations of grass biomass from Kruger National Park over 32 years (South Africa), and 3) vegetation simulations for savanna sites in Kruger via the Community Land Model version 5 (CLM5). |
| Research sample | For the meta-analysis, all trait data was extracted from existing published studies on CO2-addition experiments involving C4 grasses. For the field grass biomass data from Kruger National Park, data is annual measurements of grass biomass from sites across the park. For the vegetation modelling using CLM5, estimates of plant, soil and fire characteristics were produced for two savanna regions of Kruger National Park up to the year 2100 under different climate change scenarios. |
| Sampling strategy | For the meta-analysis of C4 grass responses to CO2 addition, studies were screened by title and abstract before being selected based on whether they met criteria relating to their suitability and relevance to the study of savanna dynamics (see methods for details).<br>The sampling strategy for the grass biomass field data at Kruger National Park was established by the Park's management team in order to monitor grass biomass to inform fire management starting in 1989. 533 sites were established across the park to sample its significant rainfall gradient and the two main parental soil types (granitic and basaltic).<br>For the vegetation modelling using CLM5, estimates of plant, soil and fire characteristics were produced for two C4-grass dominated savanna regions of Kruger National Park with different rainfall conditions: a drier site in the north of Kruger (average 420 mm rainfall yr-1) and a wetter site at the southern end of the park (average 620 mm rainfall yr-1). |
| Data collection | For the meta-analysis of C4 grass responses to CO2 addition, data was extracted from all relevent studies (either from the main body |

| | |
|---|---|
| Data collection | of the text, tables or supplementary information, or manually digitized from figures using WebPlotDigitizer; when data was not available, authors were contacted directly to request data). The grass biomass field data comes from Kruger National Park. Grass biomass was measured every year in February/March (the end of the growing season) in plots of 50m by 60m with a calibrated disc pasture meter. Measurements to derive plot-level grass biomass estimates were taken every two meters along four 50m transects, running at 0m, 20m, 40m and 60m along the length of the plot. |
| Timing and spatial scale | For the meta-analysis of C4 grass responses to CO2 addition, any study discovered in our search of the published literature that fulfilled the inclusion criteria (detailed information in the methods) was including regardless of when or where carried out. The grass biomass dataset coming from sites across Kruger National Park ranges from 1989 (when the dataset started) until 2021 (the most recent data available). For the vegetation modelling using CLM5, future simulations for the two regions of Kruger (in the far north and south of the park) were run from 2015 to 2100. |
| Data exclusions | For the grass biomass data from Kruger National Park, statistical analyses were performed on all data except data from the year 2000, when record rainfall resulted in widespread flooding (mostly from surface runoff) and thus a vegetation response that is uncoupled from total rainfall amount. |
| Reproducibility | Grass biomass data is from field observations in Kruger National Park and therefore not experimentally derived. For the vegetation simulations using the Community Land Model v5., we evaluated above ground biomass model predictions from the model against field observations collected at the Kruger National Park for 1989-2014 at sites within 25km of the center of each modeled grid square for local validation. |
| Randomization | Not relevant for the approaches used here. |
| Blinding | Not relevant for the approaches used here. |

Did the study involve field work? ☒ Yes ☐ No

## Field work, collection and transport

| | |
|---|---|
| Field conditions | Weather stations in Kruger National Park recorded daily rainfall and temperature throughout the 32-year study period. |
| Location | Kruger National Park, South Africa (sites throughout) |
| Access & import/export | Sampling sites in the field were established by Kruger's management team in 1989, and grass biomass data has been collected every year since. Measurements were taken in the field and required no sampling of plant material. |
| Disturbance | Disc-pasture measurements are none-destructive and cause minimal disturbance. |

# Reporting for specific materials, systems and methods

We require information from authors about some types of materials, experimental systems and methods used in many studies. Here, indicate whether each material, system or method listed is relevant to your study. If you are not sure if a list item applies to your research, read the appropriate section before selecting a response.

### Materials & experimental systems

| n/a | Involved in the study | |
|---|---|---|
| ☒ | ☐ | Antibodies |
| ☒ | ☐ | Eukaryotic cell lines |
| ☒ | ☐ | Palaeontology and archaeology |
| ☒ | ☐ | Animals and other organisms |
| ☒ | ☐ | Clinical data |
| ☒ | ☐ | Dual use research of concern |
| ☐ | ☒ | Plants |

### Methods

| n/a | Involved in the study | |
|---|---|---|
| ☒ | ☐ | ChIP-seq |
| ☒ | ☐ | Flow cytometry |
| ☒ | ☐ | MRI-based neuroimaging |

## Dual use research of concern

Policy information about dual use research of concern

### Hazards

Could the accidental, deliberate or reckless misuse of agents or technologies generated in the work, or the application of information presented in the manuscript, pose a threat to:

| No | Yes | |
|---|---|---|
| ☒ | ☐ | Public health |
| ☒ | ☐ | National security |
| ☒ | ☐ | Crops and/or livestock |
| ☒ | ☐ | Ecosystems |
| ☒ | ☐ | Any other significant area |

### Experiments of concern

Does the work involve any of these experiments of concern:

| No | Yes | |
|---|---|---|
| ☒ | ☐ | Demonstrate how to render a vaccine ineffective |
| ☒ | ☐ | Confer resistance to therapeutically useful antibiotics or antiviral agents |
| ☒ | ☐ | Enhance the virulence of a pathogen or render a nonpathogen virulent |
| ☒ | ☐ | Increase transmissibility of a pathogen |
| ☒ | ☐ | Alter the host range of a pathogen |
| ☒ | ☐ | Enable evasion of diagnostic/detection modalities |
| ☒ | ☐ | Enable the weaponization of a biological agent or toxin |
| ☒ | ☐ | Any other potentially harmful combination of experiments and agents |

## Plants

| | |
|---|---|
| Seed stocks | N/A |
| Novel plant genotypes | N/A |
| Authentication | N/A |
